# Real-Time Classification of Causes of Death Using AI: Sensitivity Analysis

**DOI:** 10.2196/40965

**Published:** 2023-11-22

**Authors:** Patrícia Pita Ferreira, Diogo Godinho Simões, Constança Pinto de Carvalho, Francisco Duarte, Eugénia Fernandes, Pedro Casaca Carvalho, José Francisco Loff, Ana Paula Soares, Maria João Albuquerque, Pedro Pinto-Leite, André Peralta-Santos

**Affiliations:** 1 Direção de Serviços de Informação e Análise Direção-Geral da Saúde Lisbon Portugal; 2 Unidade de Saúde Pública Zé Povinho Agrupamento de Centros de Saúde do Oeste Norte Administração Regional de Saúde de Lisboa e Vale do Tejo Caldas da Rainha Portugal; 3 NOVA National School of Public Health Universidade NOVA de Lisboa Lisbon Portugal; 4 Unidade de Saúde Pública Higeia Agrupamento de Centros de Saúde de Almada-Seixal Administração Regional de Saúde de Lisboa e Vale do Tejo Almada Portugal; 5 Unidade de Saúde Pública do Litoral Alentejano Unidade Local de Saúde do Litoral Alentejano Administração Regional de Saúde do Alentejo Santiago do Cacém Portugal; 6 Lisbon Portugal; 7 phiStat – Statistical Consulting Lisbon Portugal; 8 NOVA National School of Public Health Public Health Research Centre Universidade NOVA de Lisboa Lisbon Portugal; 9 Comprehensive Health Research Centre Universidade NOVA de Lisboa Lisbon Portugal

**Keywords:** artificial intelligence, AI, mortality, deep neural networks, evaluation, machine learning, deep learning, mortality statistics, underlying cause of death

## Abstract

**Background:**

In 2021, the European Union reported >270,000 excess deaths, including >16,000 in Portugal. The Portuguese Directorate-General of Health developed a deep neural network, AUTOCOD, which determines the primary causes of death by analyzing the free text of physicians’ death certificates (DCs). Although AUTOCOD’s performance has been established, it remains unclear whether its performance remains consistent over time, particularly during periods of excess mortality.

**Objective:**

This study aims to assess the sensitivity and other performance metrics of AUTOCOD in classifying underlying causes of death compared with manual coding to identify specific causes of death during periods of excess mortality.

**Methods:**

We included all DCs between 2016 and 2019. AUTOCOD’s performance was evaluated by calculating various performance metrics, such as sensitivity, specificity, positive predictive value (PPV), and *F*_1_-score, using a confusion matrix. This compared *International Statistical Classification of Diseases and Health-Related Problems, 10th Revision* (ICD-10), classifications of DCs by AUTOCOD with those by human coders at the Directorate-General of Health (gold standard). Subsequently, we compared periods without excess mortality with periods of excess, severe, and extreme excess mortality. We defined excess mortality as 2 consecutive days with a *Z* score above the 95% baseline limit, severe excess mortality as 2 consecutive days with a *Z* score >4 SDs, and extreme excess mortality as 2 consecutive days with a *Z* score >6 SDs. Finally, we repeated the analyses for the 3 most common ICD-10 chapters focusing on block-level classification.

**Results:**

We analyzed a large data set comprising 330,098 DCs classified by both human coders and AUTOCOD. AUTOCOD demonstrated high sensitivity (≥0.75) for 10 ICD-10 chapters examined, with values surpassing 0.90 for the more prevalent chapters (chapter II—“Neoplasms,” chapter IX—“Diseases of the circulatory system,” and chapter X—“Diseases of the respiratory system”), accounting for 67.69% (223,459/330,098) of all human-coded causes of death. No substantial differences were observed in these high-sensitivity values when comparing periods without excess mortality with periods of excess, severe, and extreme excess mortality. The same holds for specificity, which exceeded 0.96 for all chapters examined, and for PPV, which surpassed 0.75 in 9 chapters, including the more prevalent ones. When considering block classification within the 3 most common ICD-10 chapters, AUTOCOD maintained a high performance, demonstrating high sensitivity (≥0.75) for 13 ICD-10 blocks, high PPV for 9 blocks, and specificity of >0.98 in all blocks, with no significant differences between periods without excess mortality and those with excess mortality.

**Conclusions:**

Our findings indicate that, during periods of excess and extreme excess mortality, AUTOCOD’s performance remains unaffected by potential text quality degradation because of pressure on health services. Consequently, AUTOCOD can be dependably used for real-time cause-specific mortality surveillance even in extreme excess mortality situations.

## Introduction

### Background

In 2021, over 270,000 excess deaths were registered in the European Union, with >16,000 attributable to Portugal [1]. Although most of these excess deaths were possibly related to the COVID-19 pandemic, excess deaths are generally attributable to preventable causes, making a case for the importance of real-time cause-specific mortality surveillance and the subsequent timely and appropriate public health response and suitable health policies in periods of excess mortality [2].

The Portuguese Directorate-General of Health (DGS) is responsible for processing data from the Death Certificate Information System (SICO) and ensuring the epidemiological surveillance of mortality [3]. SICO all-cause mortality data are automatically analyzed and can be publicly accessed [4]. However, the analysis of death certificates (DCs) requires manual coding of the primary causes of death according to the *International Statistical Classification of Diseases and Health-Related Problems, 10th Revision* (ICD-10) [5]. This manual coding is a resource-intensive task that hinders real-time cause-specific mortality surveillance.

Excess mortality is defined by the World Health Organization as mortality above what would be expected. It allows for assessing the magnitude of a potential public health crisis by checking the additional deaths compared with a reference period and subsequently analyzing their causes in depth [6,7].

Excess mortality can be estimated in several ways. In Portugal, a period of excess mortality is defined as a consecutive period starting with 2 observed numbers of deaths above the baseline’s upper 95% confidence limit or with only 1 observed number of deaths above the upper 99% confidence limit of the baseline. The period ends with 2 consecutive values below this limit [8]. This methodology is aligned with the practice of the European mortality monitoring project (EuroMOMO), which allows for the detection and measurement in real time of periods of excess mortality from all causes as a result of threats to public health in Europe [9].

Most excess mortality surveillance systems such as EuroMOMO or national systems are based on all-cause mortality surveillance to ensure real-time surveillance. However, in many countries, information on cause of death is not readily available as it requires a human step to code the basic cause of death, delaying the surveillance and monitoring of cause-specific mortality. For instance, in Portugal, the manual establishment of the primary causes of death for the previous year is completed by March of the following year [10,11].

To overcome this problem, Portugal developed a deep neural network called AUTOCOD [12,13], which allows for presuggesting primary causes of mortality based on historical data of DCs (except for neonatal and perinatal mortality), achieving accuracies of 89% and 81% for ICD-10 chapters and blocks, respectively. AUTOCOD can also analyze data from autopsy reports and clinical bulletins (deaths occurring in health care facilities). Ultimately, the developed algorithm increased the productivity of coders, sped up the issuance of results and information, and ensured near–real-time mortality surveillance [12,13].

To our knowledge, no widespread dissemination of complex artificial intelligence (AI) algorithms can suggest underlying causes of death through free-text analysis of DCs in the same way as AUTOCOD [14].

### Objectives

This study aimed to determine the sensitivity and specificity of AUTOCOD for classifying the underlying cause of death compared with manual coding to ascertain the specific causes of death in periods of excess mortality.

AUTOCOD has already proven to have high sensitivity, specificity, and accuracy in periods without excess mortality. However, it was still being determined whether this performance would be maintained in periods of excess mortality, in which the recording of free text in DCs could change owing to the pressure felt in health services and the need to respond to more requests for DCs. A satisfactory performance by AUTOCOD could pave the way for its implementation as a real-time surveillance tool to monitor cause-specific mortality even during periods in which the national health system experiences severe pressure [14,15].

## Methods

### Study Population

In this study, we included all DCs registered in Portugal’s SICO starting from January 1, 2016, to August 8, 2019. We excluded DCs referring to neonatal, perinatal, and maternal mortality as the AUTOCOD algorithm is not trained for these underlying causes of death [13]. Each DC was manually classified according to the ICD-10 by human coders at the DGS (gold standard) or automatically by AUTOCOD.

### Study Design and Data Sets

The methods behind the construction of the AUTOCOD algorithm have been explained in detail in previous publications. The algorithm was initially trained and tested using a data set different from the one chosen for this study [12,13]. The manual codification of causes of death adheres to the World Health Organization Nomenclature Regulations specified in the ICD-10. In addition, it uses the ICD-10 rules for selecting the underlying cause of death as the primary cause of death by international rules [5].

The DC data set was then linked with 2 dictionaries of the ICD-10 to translate block and chapter codes into text descriptions. The DC data set was also linked to the national surveillance all-cause mortality data set [4], which defines the baseline for expected deaths according to the EuroMOMO methodology [16] and the daily count of observed deaths.

### Excess Mortality Definition

**Figure 1:**
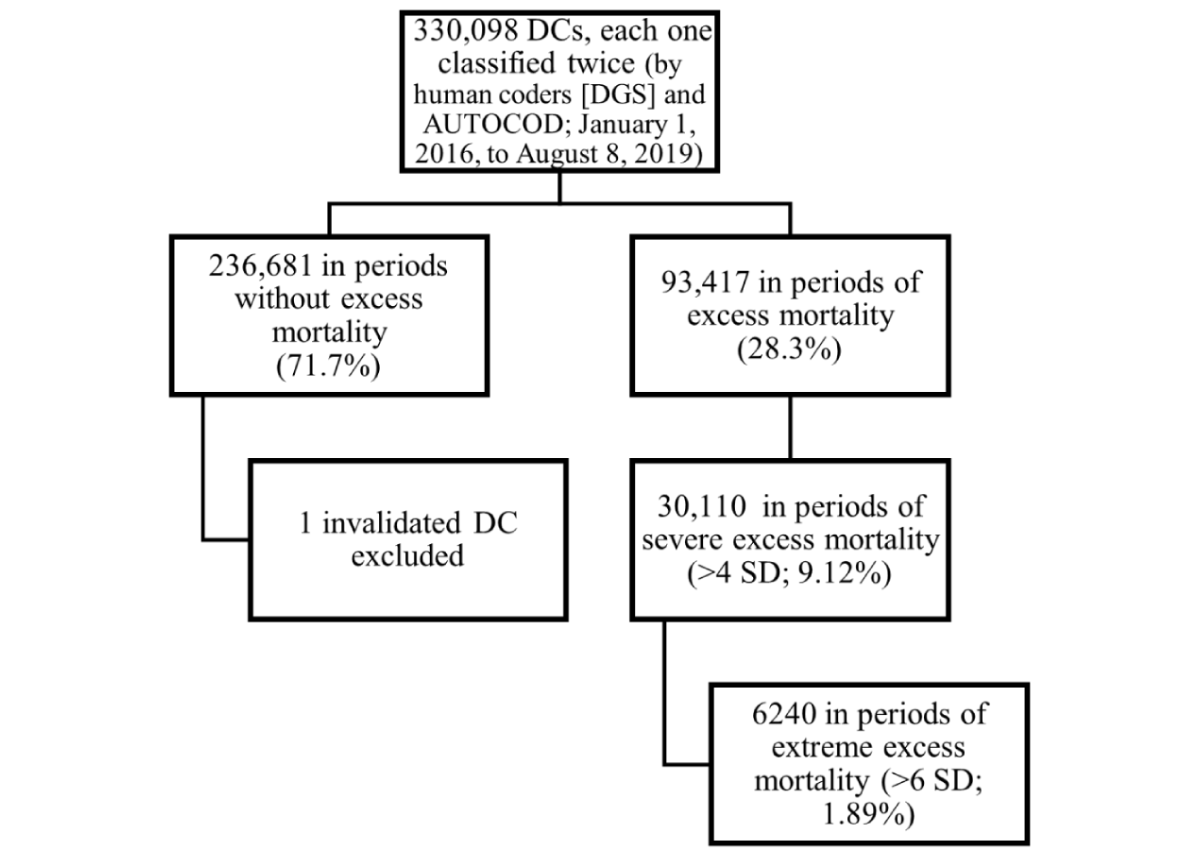
Flowchart of the study population inclusion criteria. DC: death certificate; DGS: Directorate-General of Health.

Using this data set, we defined the periods in which excess mortality was observed according to the EuroMOMO *Z* score for excess mortality and the rules of Westgard [17] (ie, we considered excess mortality when there were 2 consecutive days with a *Z* score above the limit at 95% of the baseline or just 1 day at >99%). The period of excess mortality ended with 2 consecutive days below the limit of 95% of the baseline. Flowchart of the study population inclusion criteria can be found in Figure 1.

We also defined 2 metrics for periods of severe and extreme excess mortality. These were 2 consecutive days with a *Z* score above the limit of 4 SDs and 6 SDs, respectively. The Westgard functions used to classify the different periods can be found in Multimedia Appendix 1 [17-19].

### Statistical Analysis

To obtain the multiclass confusion matrix, we used the “confusionMatrix” function of the *caret* package in RStudio (version 6.0-90; Posit, PBC) [18,19]. In a multiclass problem such as classifying ICD-10 chapters and blocks, the “confusionMatrix” will show a set of “one-versus-all” results. For example, in a 3-class problem, the sensitivity of the first class is calculated against all the samples in the second and third classes (and so on). The resulting confusion matrix summarizes the prediction results for a classification problem.

The number of correct and incorrect predictions is summarized with count values and broken down by each class. The confusion matrix shows how a classification model such as AUTOCOD is confused when it makes predictions. These numbers are then organized into a table or matrix. Each row of the matrix corresponds to a predicted class (ie, AUTOCOD). Each matrix column corresponds to an actual class (ie, human coders at the DGS).

The numbers of correct and incorrect classifications are then filled into the table. The total number of correct predictions for a class goes into the expected row for that class value and the predicted column for that class value. In the same way, the total number of incorrect predictions for a class goes into the expected row for that class value and the predicted column for that class value.

Finally, we performed a sensitivity analysis (also using the R package *caret*) to compare the classification results obtained using the AUTOCOD algorithm (index test) with the classification made by human coders (gold standard) [20]. This allowed us to obtain the number of true positives and false positives as well as additional metrics such as sensitivity (recall), specificity, accuracy, positive predictive value (PPV), and *F*_1_-score [13]. This step was performed over time, including a comparison between periods of excess and no excess mortality and between periods of extreme excess mortality and no excess mortality both by chapter and block classification levels of the ICD-10 [13]. We present this comparison as the difference in absolute values and with the Kullback-Leibler divergence (KLD), which measures the distribution of a metric and chapter or block during a specific period of excess or extreme mortality and periods of no excess mortality. In other words, the KLD measures the difference between 2 probability distributions. We used the kullback_leibler_distanc function of the R package *philentropy* [21].

The formulas used for all these performance metrics can be found in Table S1 in Multimedia Appendix 1 [17-19].

To assess the quality of AUTOCOD, we opted to present the weighted average of performance metrics such as sensitivity, precision, and *F*_1_-scores by taking the mean of all class performance metrics while considering each class’s number of actual occurrences in the data set. The “weight” refers to the proportion of each class’s actual occurrences in the data set relative to the sum of all occurrences. The full formula for this calculation of the weighted average is provided in Multimedia Appendix 1 [17-19]. This choice was made as opposed to presenting the macroaverage of performance metrics (ie, macroaverages assign equal importance to each chapter or block, thus calculating the arithmetic mean of performance metrics) [13] as the latter methodology would artificially increase the importance of the average of the rare or infrequent cause of death chapters and blocks.

In the data set, 1 DC was not adequately codified by AUTOCOD, so the ICD-10 classifications of that DC from both AUTOCOD and the DGS were excluded.

All analyses were performed using R statistical software (version 4.1.2; R Foundation for Statistical Computing) [22-25]. The analyses were checked by 2 researchers.

### Ethical Considerations

The DGS is the national entity responsible for data treatment and data protection of the SICO. The data provided were only for the purposes strictly necessary for this study within the competencies of the DGS. Data were previously anonymized. Patient consent was waived as the data were deidentified and processed for reasons of public interest in public health. This research received previous authorization from the DGS following positive advice from its data protection officer. In this way, the research complies with the best practices of the General Data Protection Regulation. This study was exempt from an ethics review board assessment following the self-assessment checklist for ethics of the Ethics Committee of the National School of Public Health [26].

## Results

### Description of the Data Set

The data set (Table 1) comprised 330,098 DCs, each classified twice, meaning that we had all DCs classified by human coders and by AUTOCOD. The 3 most common ICD-10 chapters classified by human coders were chapter IX—“Diseases of the circulatory system” (97,420/330,098, 29.51%), chapter II—“Neoplasms” (85,837/330,098, 26%), and chapter X—“Diseases of the respiratory system” (40,202/330,098, 12.18%). A more extensive and detailed descriptive analysis of this data set can be found in Multimedia Appendix 1 [17-19], including the desegregation of DCs by year, ICD-10 chapter or block, and period.

As expected, there were fewer DCs for periods of excess mortality (n=186,834; 93,417/330,098, 28.3% of the total DCs from each source) than for periods without excess mortality (n=473,362; 236,681/330,098, 71.7% of the total DCs from each source). When considering the periods of severe and extreme excess mortality either for *Z* scores of >4 SDs (n=60,220; 30,110/330,098, 9.12% from each source) or *Z* scores of >6 SDs (n=12,480; 6240/330,098, 1.89% from each source), the DCs were even fewer.

Considering only the 3 most common chapters of the data set (chapters II, IX, and X), we performed the same analysis for the classification of ICD-10 blocks (Table 2), which accounted for 67.69% (223,459/330,098) of the total DCs throughout the period. The 5 most common blocks classified in DCs were C00-C97 (malignant neoplasms), I60-I69 (cerebrovascular diseases), I30-I52 (other forms of heart disease), I20-I25 (ischemic heart disease), and J09-J18 (influenza and pneumonia).

**Table 1:** Description of the study population by excess mortality and type of death certificate coding (N=330,098)^a^.

Chapter	No excess mortality, n/N (%)			Excess mortality, n/N (%)			Severe excess mortality (>4 SDs), n/N (%)			Extreme excess mortality (>6 SDs), n/N (%)			Total, n/N (%)	
	Human	AUTOCOD	Human		AUTOCOD	Human		AUTOCOD	Human		AUTOCOD	Human		AUTOCOD
I^b^	4460/ 6154 (72.45)	4863/ 6649 (73.14)	1696/ 6156 (27.55)		1786/6649 (26.86)	546/ 6154 (8.87)		566/ 6649 (8.51)	111/ 6154 (1.8)		117/ 6649 (1.76)	6156/ 330,098 (1.86)		6649/ 330,098 (2.01)
II^c^	64,701/ 85,837 (75.38)	62,895/ 83,462 (75.36)	21,136/ 85,837 (24.62)		20,567/ 83,462 (24.64)	6088/ 85,837 (7.09)		5941/ 83,462 (7.12)	1166/ 85,837 (1.36)		1162/ 83,462 (1.39)	85,837/ 330,098 (26)		83,462/ 330,098 (25.28)
III^d^	916/ 1334 (68.67)	1152/ 1602 (71.91)	418/ 1334 (31.33)		450 /1602 (28.09)	139/ 1334 (10.42)		150/ 1602 (9.36)	22/ 1334 (1.65)		25 /1602 (1.56)	1334/ 330,098 (0.4)		1602/ 330,098 (0.49)
IV^e^	11,637/ 16,430 (70.83)	13,727/ 19,382 (70.82)	4793/ 16,430 (29.17)		5655/ 19,382 (29.18)	1594/ 16,430 (9.7)		1880 19,382 (9.7)	313/ 16,430 (1.91)		374/ 19,382 (1.93)	16,430/ 330,098 (4.98)		19,382/ 330,098 (5.87)
V^f^	8986/ 12,742 (70.52)	8512/ 12,172 (69.93)	3756/ 12,742 (29.48)		3660/ 12,172 (30.07)	1264/ 12,742 (9.92)		1221/ 12,172 (10.03)	281/ 12,742 (2.21)		261/ 12,172 (2.14)	12,742/ 330,098 (3.86)		12,172/ 330,098 (3.69)
VI^g^	8354/ 11,810 (70.74)	7757/ 10,997 (70.54)	3456/ 11,810 (29.26)		3240/ 10,997 (29.46)	1097/ 11,810 (9.29)		1024/ 10,997 (9.31)	254/ 11,810 (2.15)		228/ 10,997 (2.07)	11,810/ 330,098 (3.58)		10,997/ 330,098 (3.33)
VII^h^	1/2 (50)	—^i^	1/2 (50)		—	0/2 (0)		—	0/2 (0)		—	2/330,098 (0)		0/330,098 (0)
VIII^j^	22/30 (73)	6/9 (66.67)	8/30 (26.67)		3/9 (33.33)	5/30 (16.67)		3/9 (33.33)	0/30 (0)		0/9 (0)	30/330,098 (0.01)		9/330,098 (0)
IX^k^	69,021/ 97,420 (70.85)	68,850/ 97,252 (70.8)	28,399/ 97,420 (29.15)		28,402/ 97,252 (29.2)	9287/ 97,420 (9.53)		9296/ 97,252 (9.56)	1918/ 97,420 (1.97)		1937 /97,252 (1.99)	97,420/ 330,098 (29.51)		97,252/ 330,098 (29.46)
X^l^	26,736/ 40,202 (66.5)	28,913/ 43,057 (67.15)	13,466/ 40,202 (33.5)		14,144/ 43,057 (32.85)	4734/ 40,202 (11.78)		4934/ 43,057 (11.46)	1014/ 40,202 (2.52)		1050/ 43,057 (2.44)	40,202/ 330,098 (12.18)		43,057/ 330,098 (13.04)
XI^m^	10,999/ 14,892 (73.86)	10,382/ 13,967 (74.33)	3893/ 14,892 (26.14)		3585/ 13,967 (25.67)	1201/ 14,892 (8.06)		1108/ 13,967 (7.93)	217/ 14,892 (1.46)		195/ 13,967 (1.4)	14,892/ 330,098 (4.51)		13,967/ 330,098 (4.23)
XII^n^	430/583 (73.76)	252/348 (72.41)	153/583 (26.24)		96/348 (27.59)	38/583 (6.52)		28/348 (8.05)	5/583 (0.86)		3/348 (0.86)	583/330,098 (0.18)		348/330,098 (0.11)
XIII^o^	991/1397 (70.94)	684/960 (71.25)	406/1397 (29.06)		276/960 (28.75)	130/1397 (9.31)		96/960 (10)	28/1397 (2)		18/960 (1.88)	1397/330,098 (0.42)		960/330,098 (0.29)
XIV^p^	7426/ 10,277 (72.26)	7499/ 10,389 (72.18)	2851/ 10,277 (27.74)		2890/ 10,389 (27.82)	924/ 10,277 (8.99)		927/ 10,389 (8.92)	179/ 10,277 (1.74)		174/ 10,389 (1.67)	10,277/ 330,098 (3.11)		10,389/ 330,098 (3.15)
XV^q^	29/35 (82.86)	—	6/35 (17.14)		—	2/35 (5.71)		—	1/35 (2.86)		—	35/330,098 (0.01)		0/330,098 (0)
XVI^r^	46/58 (79.31)	3/3 (100)	12/58 (20.69)		0/3 (0)	5/58 (8.62)		0/3 (0)	0/58 (0)		0/3 (0)	58/330,098 (0.02)		3/330,098 (0)
XVII^s^	357/494 (72.27)	181/246 (73.58)	137/494 (27.73)		65/246 (26.42)	38/494 (7.69)		17/246 (6.91)	10/494 (2.02)		3/246 (1.22)	494/330,098 (0.15)		246/330,098 (0.07)
XVIII^t^	11,072/ 16,269 (68.06)	11,641/ 17,075 (68.18)	5197/ 16,269 (31.94)		5434/ 17,075 (31.82)	1802/ 16,269 (11.08)		1879/17,075 (11)	448/ 16,269 (2.75)		454/ 17,075 (2.66)	16,269/ 330,098 (4.93)		17,075/ 330,098 (5.17)
XIX^u^	0/2 (0)	—	2/2 (100)		—	2/2 (100)		—	0/2 (0)		—	2/330,098 (0)		0/330,098 (0)
XX^v^	10,497/ 14,128 (74.3)	9363/ 12,527 (74.74)	3631/ 14,128 (25.7)		3164/ 12,527 (25.26)	1214/ 14,128 (8.59)		1040/ 12,527 (8.3)	273/ 14,128 (1.93)		239/ 12,527 (1.91)	14,128/ 330,098 (4.28)		12,527/ 330,098 (3.79)
—	—	1/1 (100)	—		0/1 (0)	—		0 /1 (0)	—		0/1 (0)	0/330,098 (0)		1/330,098 (0)
Total	236,681/ 330,098 (71.7)	236,681/ 330,098 (71.7)	93,417/ 330,098 (28.3)		93,417/ 330,098 (28.3)	30,110/ 330,098 (9.12)		30,110/ 330,098 (9.12)	6240/ 330,098 (1.89)		6240/ 330,098 (1.89)	330,098/ 330,098 (100)		330,098/ 330,098 (100)

^a^Percentage values represent the proportion of death certificates for each period analyzed considering the total of each chapter except for the total column, which gives the proportion of each chapter for all the death certificates.

^b^Certain infectious and parasitic diseases.

^c^Neoplasms.

^d^Diseases of the blood and blood-forming organs and certain disorders involving the immune system.

^e^Endocrine, nutritional, and metabolic diseases.

^f^Mental and behavioral disorders.

^g^Diseases of the nervous system.

^h^Diseases of the eye and adnexa.

^i^Missing values.

^j^Diseases of the ear and mastoid process.

^k^Diseases of the circulatory system.

^l^Diseases of the respiratory system.

^m^Diseases of the digestive system.

^n^Diseases of the skin and subcutaneous tissue.

^o^Diseases of the musculoskeletal system and connective tissue.

^p^Diseases of the genitourinary system.

^q^Pregnancy, childbirth, and the puerperium.

^r^Certain conditions originating in the perinatal period.

^s^Congenital malformations, deformations, and chromosomal abnormalities.

^t^Symptoms, signs, and abnormal clinical and laboratory findings not elsewhere specified.

^u^Injury, poisoning, and certain other consequences of external causes.

^v^External causes of morbidity and mortality.

**Table 2:** Description of the study population for the 3 most common chapters (II, IX, and X) for all the periods analyzed (N=330,098)^a^.

Block	No excess mortality, n/N (%)		Excess mortality, n/N (%)			Severe excess mortality (>4 SDs), n/N (%)			Extreme excess mortality (>6 SDs), n/N (%)			Total, n/N (%)	
	Human	AUTOCOD	Human	AUTOCOD	Human		AUTOCOD	Human		AUTOCOD	Human		AUTOCOD
C00-C97^b^	63,379/ 84,031 (75.42)	61,770/ 81,919 (75.4)	20,652/ 84,031 (24.58)	20,149/ 81,919 (24.6)	5942/ 84,031 (7.07)		5695/ 81,919 (6.95)	1141/ 84,031 (1.36)		1102/ 81,919 (1.35)	84,031/ 223,459 (37.6)		81,919/ 223,771 (36.61)
D00-D09^c^	8/9 (88.89)	—^d^	1/9 (11.11)	—	0/9 (0)		—	0/9 (0)		—	9/223,459 (0)		0/223,771 (0)
D10-D36^e^	224/311 (72.03)	171/224 (76.34)	87/311 (27.97)	53/224 (23.66)	26/311 (8.36)		14/224 (6.25)	7/311 (2.25)		4/224 (1.79)	311/223,459 (0.14)		224/223,771 (0.1)
D37-D48^f^	1090/1486 (73.35)	954/1319 (72.33)	396/1486 (26.65)	365/1319 (27.67)	120/1486 (8.08)		110/1319 (8.34)	18/1486 (1.21)		24/1319 (1.82)	1486/223,459 (0.66)		1319/223,771 (0.59)
I05-I09^g^	376/490 (76.73)	301/408 (73.77)	114/490 (23.27)	107/408 (26.23)	40/490 (8.16)		42/408 (10.29)	3/490 (0.61)		4/408 (0.98)	490/223,459 (0.22)		408/223,771 (0.18)
I10-I15^h^	5291/7611 (69.52)	6210/8938 (69.48)	2320/7611 (30.48)	2728/8938 (30.52)	810/7611 (10.64)		796/8938 (8.91)	149/7611 (1.96)		149/8938 (1.67)	7611/223,459 (3.41)		8938/223,771 (3.99)
I20-I25^i^	14,858/ 21,153 (70.24)	14,803/ 20,979 (70.56)	6295/ 21,153 (20.76)	6176/ 20,979 (29.44)	2093/ 21,153 (9.89)		1925/ 20,979 (9.18)	471/ 21,153 (2.23)		441/ 20,979 (2.1)	21,153/ 223,459 (9.47)		20,979/ 223,771 (9.38)
I26-I28^j^	1615/2314 (69.79)	1627/2296 (70.86)	699/2314 (30.21)	669/2296 (29.14)	222/2314 (9.59)		197/2296 (8.58)	43/2314 (1.86)		41/2296 (1.79)	2314/223,459 (1.04)		2296/223,771 (1.03)
I30-I52^k^	18,232/ 26,016 (70.08)	18,563/ 26,565 (69.88)	7784/ 26,016 (29.92)	8002/ 26,565 (30.12)	2490/ 26,016 (9.57)		2433/ 26,565 (9.16)	524/ 26,016 (2.01)		509/ 26,565 (1.92)	26,016/ 223,459 (11.64)		26,565/ 223,771 (11.87)
I60-I69^l^	24,836/ 34,595 (71.79)	24,133/ 33,625 (71.77)	9759/ 34,595 (28.21)	9492 /33,625 (28.23)	3162/ 4,595 (9.14)		2893/ 33,625 (8.6)	621/ 34,595 (1.8)		568/ 33,625 (1.69)	34,595/ 223,459 (15.48)		33,625/ 223,771 (15.03)
I70-I79^m^	3494/4794 (72.88)	3001/4136 (72.56)	1300/4794 (27.12)	1135/4136 (27.44)	431/4794 (8.99)		351/4136 (8.49)	102/4794 (2.13)		79/4136 (1.91)	4794/223,459 (2.15)		4136/223,771 (1.85)
I80-I89^n^	303/427 (70.96)	203/296 (68.58)	124/427 (29.04)	93/296 (31.42)	36/427 (8.43)		23/296 (7.77)	5/427 (1.17)		2/296 (0.68)	427/223,459 (0.19)		296/223,771 (0.13)
I95-I99^o^	16/20 (80)	9/9 (100)	4/20 (20)	0/9 (0)	3/20 (15)		0/9 (0)	0/20 (0)		0/9 (0)	20/223,459 (0.01)		9/223,771 (0)
J00-J06^p^	28/46 (60.87)	14/18 (77.78)	18/46 (39.13)	4/18 (22.22)	7/46 (15.22)		1/18 (5.56)	1/46 (2.17)		0/18 (0)	46/223,459 (0.02)		18/223,771 (0.01)
J09-J18^q^	11,866/18,191 (65.23)	12,417/18,775 (66.14)	6325/18,191 (34.77)	6358/18,775 (33.86)	2248/18,191 (12.36)		2082/18,775 (11.09)	481/18,191 (2.64)		441/18,775 (2.35)	18,191/223,459 (8.14)		18,775/223,771 (8.39)
J20-J22^r^	1409/2102 (67.03)	1394/2067 (67.44)	693/2102 (32.97)	673/2067 (32.56)	251/2102 (11.94)		228/2067 (11.03)	61/2102 (2.9)		55/2067 (2.66)	2102/223,459 (0.94)		2067/223,771 (0.92)
J30-J39^s^	42/53 (79.25)	30/40 (75)	11/53 (20.75)	10/40 (25)	6/53 (11.32)		2/40 (5)	0/53 (0)		0/40 (0)	53/223,459 (0.02)		40/223,771 (0.02)
J40-J47^t^	5929/8953 (66.22)	6814/10,234 (66.58)	3024/8953 (33.78)	3420/10,234 (33.42)	1070/8953 (11.95)		1113/10,234 (10.88)	232/8953 (2.59)		240/10,234 (2.35)	8953/223,459 (4.01)		10,234/223,771 (4.57)
J60-J70^u^	1649/2340 (70.47)	1692/2345 (72.15)	691/2340 (29.53)	653/2345 (27.85)	211/2340 (9.02)		180/2345 (7.68)	42/2340 (1.79)		33/2345 (1.41)	2340/223,459 (1.05)		2345/223,771 (1.05)
J80-J84^v^	1168/1636 (71.39)	1102/1544 (71.37)	468/1636 (28.61)	442/1544 (28.63)	157/1636 (9.6)		131/1544 (8.48)	28/1636 (1.71)		27/1544 (1.75)	1636/223,459 (0.73)		1544/223,771 (0.69)
J85-J86^w^	155/215 (72.09)	61/83 (73.49)	60/215 (27.91)	22/83 (26.51)	16/215 (7.44)		2/83 (2.41)	1/215 (0.47)		0/83 (0)	215/223,459 (0.1)		83/223,771 (0.04)
J90-J94^x^	160/221 (72.4)	182/249 (73.09)	61/221 (27.6)	67/249 (26.91)	22/221 (9.95)		17/249 (6.83)	5/221 (2.26)		3/249 (1.2)	221/223,459 (0.1)		249/223,771 (0.11)
J95-J99^y^	4330/ 6445 (67.18)	5207/ 7702 (67.61)	2115/ 6445 (32.82)	2495/ 7702 (32.39)	746/ 6445 (11.57)		745/ 7702 (9.67)	163/ 6445 (2.53)		160/ 7702 (2.08)	6445/ 223,459 (2.88)		7702 /223,771 (3.44)
Total	160,458/ 223,459 (71.81)	160,658/ 223,771 (71.8)	63,001/ 223,459 (28.19)	63,113/ 223,771 (28.2)	20,109/ 223,459 (9)		18,980/ 223,771 (8.48)	4098/ 223,459 (1.83)		3882/ 223,771 (1.73)	223,459/ 223,459 (100)		223,771/ 223,771 (100)

^a^Percentage values represent the proportion of death certificates for each period analyzed considering the total of each block except for the total column, which gives the proportion of each block for all the death certificates.

^b^Malignant neoplasms.

^c^In situ neoplasms.

^d^Missing values.

^e^Benign neoplasms.

^f^Neoplasms of uncertain or unknown behavior.

^g^Chronic rheumatic heart diseases.

^h^Hypertensive diseases.

^i^Ischemic heart diseases.

^j^Pulmonary heart disease and diseases of pulmonary circulation.

^k^Other forms of heart disease.

^l^Cerebrovascular diseases.

^m^Diseases of the arteries, arterioles, and capillaries.

^n^Diseases of the veins, lymphatic vessels, and lymph nodes not elsewhere classified.

^o^Other and unspecified disorders of the circulatory system.

^p^Acute upper respiratory infections.

^q^Influenza and pneumonia.

^r^Other acute lower respiratory infections.

^s^Other diseases of the upper respiratory tract.

^t^Chronic lower respiratory diseases.

^u^Lung diseases owing to external agents.

^v^Other respiratory diseases principally affecting the interstitium.

^w^Suppurative and necrotic conditions of the lower respiratory tract.

^x^Other diseases of the pleura.

^y^Other diseases of the respiratory system.

### Results for ICD-10 Chapters

The *caret* package provides the confusion matrix, which evaluates AUTOCOD’s performance by calculating some performance metrics. The full performance metrics calculated for AUTOCOD can be found in Multimedia Appendix 1 [17-19].

As presented in Table S2 in Multimedia Appendix 1 [17-19], the specificity in all ICD-10 chapters was >0.97 for periods without excess mortality. The highest values of sensitivity (or recall) were for chapter II—“Neoplasms” (0.95), chapter XVIII—“Symptoms, signs, and abnormal clinical and laboratory findings not elsewhere specified” (0.93), and chapter IX—“Diseases of the circulatory system” (0.91). Considering the PPV (or precision), the highest values were for chapter XVI—“Certain conditions originating in the perinatal period” (1.00), chapter II—“Neoplasms” (0.98), and chapter IX—“Diseases of the circulatory system” (0.92). The highest *F*_1_-scores were for chapter II—“Neoplasms” (0.96), chapter IX—“Diseases of the circulatory system” (0.91), and chapter XVIII—“Symptoms, signs, and abnormal clinical and laboratory findings not elsewhere specified” (0.90).

Specificity in all ICD-10 chapters was >0.96 for the excess mortality periods. The highest values of sensitivity (or recall) were for chapter II—“Neoplasms” (0.95), chapter XVIII—“Symptoms, signs, and abnormal clinical and laboratory findings not elsewhere specified” (0.93), and chapter IX—“Diseases of the circulatory system” (0.91). Considering the PPV (or precision), the highest values were for chapter II—“Neoplasms” (0.97), chapter IX—“Diseases of the circulatory system” (0.91), and chapter XVIII—“Symptoms, signs, and abnormal clinical and laboratory findings not elsewhere specified” (0.88). The highest *F*_1_-scores were for chapter II—“Neoplasms” (0.96), chapter IX—“Diseases of the circulatory system” (0.91), and chapter XVIII—“Symptoms, signs, and abnormal clinical and laboratory findings not elsewhere specified” (0.90).

Specificity in periods with severe excess mortality (>4 SDs) was >0.96 in all ICD-10 chapters. The highest values of sensitivity (or recall) were for chapter II—“Neoplasms” (0.94), chapter XVIII—“Symptoms, signs, and abnormal clinical and laboratory findings not elsewhere specified” (0.92), and chapter IX—“Diseases of the circulatory system” (0.91). Considering the PPV (or precision), the highest values were for chapter II—“Neoplasms” (0.97), chapter IX—“Diseases of the circulatory system” (0.91), and chapter XVIII—“Symptoms, signs, and abnormal clinical and laboratory findings not elsewhere specified” (0.88). The highest *F*_1_-scores were for chapter II—“Neoplasms” (0.96), chapter IX—“Diseases of the circulatory system” (0.91), and chapter XVIII—“Symptoms, signs, and abnormal clinical and laboratory findings not elsewhere specified (0.90).

For periods with extreme excess mortality (>6 SDs), specificity in all ICD-10 chapters was >0.96. The highest values of sensitivity (or recall) were for chapter II—“Neoplasms” (0.95), chapter IX—“Diseases of the circulatory system” (0.91), and chapter XVIII—“Symptoms, signs, and abnormal clinical and laboratory findings not elsewhere specified” (0.90). Considering the PPV (or precision), the highest values were for chapter II—“Neoplasms” (0.96), chapter IX—“Diseases of the circulatory system” (0.90), and chapter XVIII—“Symptoms, signs, and abnormal clinical and laboratory findings not elsewhere specified” (0.88). The highest *F*_1_-scores were for chapter II—“Neoplasms” (0.96), chapter IX—“Diseases of the circulatory system” (0.91), and chapter XVIII—“Symptoms, signs, and abnormal clinical and laboratory findings not elsewhere specified” (0.89).

Considering the weighted average of all chapters, the results we obtained for the performance metrics of AUTOCOD are presented in Table 3. For sensitivity, PPV, and *F*_1_-score, there was no difference between periods without excess mortality and those with excess mortality (<0.01). There was a decrease of 0.01 from periods without excess mortality to periods with severe excess mortality (>4 SDs). There was a decrease of 0.04 when comparing the weighted average of periods without excess mortality and periods with extreme excess mortality (>6 SDs).

**Table 3:** Average performance metrics for different periods for the International Statistical Classification of Diseases and Health-Related Problems, 10th Revision, chapter classification of AUTOCOD.

	Sensitivity (weighted average)	Specificity (weighted average)	Positive predictive value (weighted average)	*F*_1_-score (weighted average)
No excess mortality	0.88	0.98	0.88	0.88
Excess mortality	0.88	0.98	0.88	0.88
Severe excess mortality (>4 SDs)	0.87	0.98	0.87	0.87
Extreme excess mortality (>6 SDs)	0.85	0.94	0.84	0.84

It is vital to analyze the differences between periods without excess mortality and periods of excess mortality, severe excess mortality, or extreme excess mortality and which chapters perform better.

According to Table 4, the biggest differences in the sensitivity values of AUTOCOD between periods without excess mortality and periods with excess mortality were found in chapter XVI—“Certain conditions originating in the perinatal period” (0.07), chapter XVII—“Congenital malformations, deformations, and chromosomal abnormalities” (0.05), chapter VIII—“Diseases of the ear and mastoid process” (−0.07), and chapter XII—“Diseases of the skin and subcutaneous tissue” (−0.08). For the 3 most common chapters, the differences were 0.00 (chapter II—“Neoplasms”), 0.00 (chapter IX—“Diseases of the circulatory system”), and 0.01 (chapter X—“Diseases of the respiratory system”). Regarding the differences in sensitivity values between periods without excess mortality and periods of severe excess mortality (*Z* score of >4 SDs), the biggest differences were found in chapter VIII—“Diseases of the ear and mastoid process” (−0.22), chapter XII—“Diseases of the skin and subcutaneous tissue” (−0.12), chapter XVI—“Certain conditions originating in the perinatal period” (0.07), and chapter XVII—“Congenital malformations, deformations, and chromosomal abnormalities” (0.07). For the 3 most common chapters, the differences were 0.01 (chapter II—“Neoplasms”), 0.01 (chapter IX—“Diseases of the circulatory system”), and 0.00 (chapter X—“Diseases of the respiratory system”). When comparing the difference between the sensitivity values of AUTOCOD for periods without excess mortality and periods of extreme excess mortality (*Z* score of >6 SDs), the biggest differences were found in chapter XVII—“Congenital malformations, deformations, and chromosomal abnormalities” (0.19), chapter III—“Diseases of the blood and blood-forming organs and certain disorders involving the immune system” (0.17), chapter XIII—“Diseases of the musculoskeletal system and connective tissue” (0.10), and chapter XII—“Diseases of the skin and subcutaneous tissue” (0.08). For the 3 most common chapters, the differences were 0.00 (chapter II—“Neoplasms”), 0.00 (chapter IX—“Diseases of the circulatory system”), and 0.00 (chapter X—“Diseases of the respiratory system”).

**Table 4:** Comparison among sensitivity values of AUTOCOD depending on the period (without excess mortality and with excess mortality, severe excess mortality, or extreme excess mortality) by chapter of the International Statistical Classification of Diseases and Health-Related Problems, 10th Revision.

Chapter	No excess mortality	Excess mortality	Difference (no excess mortality–excess mortality)	KLD^a^ (no excess mortality and excess mortality)	Severe excess mortality (>4 SDs)	Difference (>4 SDs–no excess mortality)	KLD (no excess mortality and >4 SDs)	Extreme excess mortality (>6 SDs)	Difference (>6 SDs–no excess mortality)	KLD (no excess mortality and >6 SDs)
I	0.67	0.67	<0.01	0.00	0.67	0.00	0.00	0.65	0.02	0.02
II	0.95	0.95	0.00	0.00	0.94	0.01	0.01	0.95	0.00	0.00
III	0.57	0.55	0.02	0.02	0.58	0.00	0.00	0.41	0.17	0.19
IV	0.81	0.81	0.00	0.00	0.81	0.00	0.00	0.82	−0.02	−0.02
V	0.77	0.78	0.00	0.00	0.78	0.00	0.00	0.77	0.01	0.01
VI	0.79	0.80	−0.01	−0.01	0.79	0.00	0.00	0.79	0.01	0.01
VII	0.00	0.00	0.00	0.00	N/A^b^	N/A	N/A	N/A	N/A	N/A
VIII	0.18	0.25	−0.07	−0.06	0.40	−0.22	−0.14	N/A	N/A	N/A
IX	0.91	0.91	0.00	0.00	0.91	0.01	0.01	0.91	0.00	0.00
X	0.90	0.89	0.01	0.01	0.90	0.00	0.00	0.89	0.00	0.00
XI	0.80	0.79	0.02	0.02	0.76	0.04	0.04	0.76	0.04	0.04
XII	0.28	0.35	−0.08	−0.07	0.40	−0.12	−0.10	0.20	0.08	0.09
XIII	0.42	0.42	0.00	0.00	0.42	0.00	0.00	0.32	0.10	0.11
XIV	0.76	0.76	0.01	0.01	0.76	0.00	0.00	0.74	0.02	0.02
XV	0.00	0.00	0.00	0.00	0.00	0.00	0.00	0.00	0.00	0.00
XVI	0.07	0.00	0.07	0.57	0.00	0.07	0.57	N/A	N/A	N/A
XVII	0.39	0.34	0.05	0.06	0.32	0.07	0.08	0.20	0.19	0.26
XVIII	0.93	0.93	0.00	0.00	0.92	0.01	0.01	0.90	0.03	0.03
XIX	N/A	0.00	N/A	N/A	0.00	N/A	N/A	N/A	N/A	N/A
XX	0.79	0.76	0.02	0.02	0.75	0.04	0.04	0.76	0.02	0.02

^a^KLD: Kullback-Leibler divergence.

^b^N/A: not applicable.

In addition, Table 4 shows the KLD between periods without excess mortality and periods of excess mortality. For 9 chapters, including 2 of the most prevalent (chapter II—“Neoplasms” and chapter IX—“Diseases of the circulatory system”), the KLD was 0, indicating that the distribution of values for periods of excess mortality was similar to that for periods of no excess mortality. For other chapters, such as chapter X—“Diseases of the respiratory system,” the KLD was close to 0. In chapter XVI—“Certain conditions originating in the perinatal period,” the KLD was particularly high, implying a large difference in the probability distributions. Regarding the KLD between periods without excess mortality and periods of extreme excess mortality (*Z* score of >4 SDs), the sensitivity had a KLD of 0 for 9 chapters, including chapter X—“Diseases of the respiratory system.” It also had a KLD close to 0 for chapter II—“Neoplasms” and chapter IX—“Diseases of the circulatory system.” When comparing the difference between the KLD for the sensitivity of AUTOCOD for periods without excess mortality and periods of extreme excess mortality (*Z* score of >6 SDs), sensitivity had a KLD of 0 in the 3 most prevalent chapters as well as chapter XV—“Pregnancy, childbirth, and the puerperium.”

**Figure 2:**
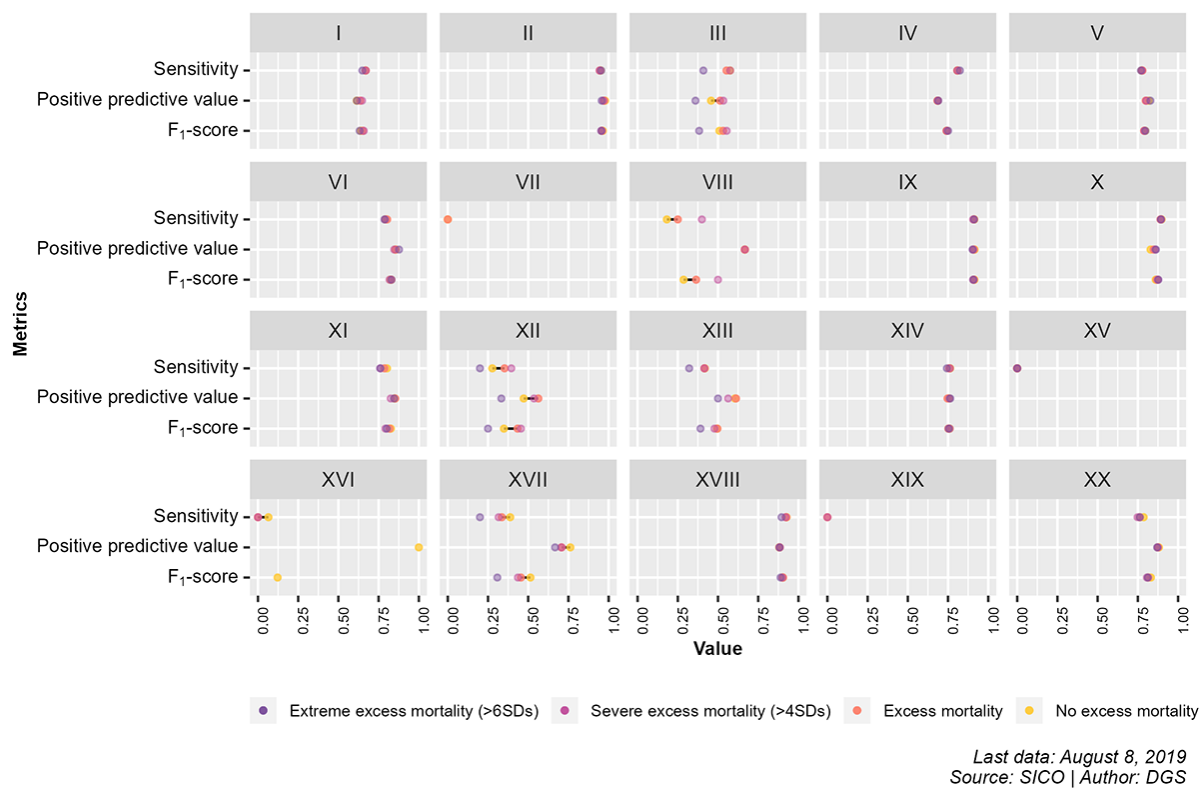
Comparison between performance metrics of AUTOCOD during periods of excess mortality, severe excess mortality, and extreme excess mortality and periods without excess mortality for International Statistical Classification of Diseases and Health-Related Problems, 10th Revision (ICD-10), chapters. DGS: Directorate-General of Health; SICO: Death Certificate Information System.

The differences in the performance measures of AUTOCOD between periods without excess mortality and periods of excess or extreme excess mortality are shown in Figure 2. The absolute values of the observations for each period analyzed and additional comparisons of AUTOCOD performance measures can be found in Multimedia Appendix 1 [17-19].

### Results for ICD-10 Blocks

This section analyzes the ICD-10 classification by blocks for only the 3 most common chapters (chapter II—“Neoplasms,” chapter IX—“Diseases of the circulatory system,” and chapter X—“Diseases of the respiratory system”).

As presented in Table S3 in Multimedia Appendix 1 [17-19], specificity in all ICD-10 blocks was >0.99 for periods without excess mortality. The highest values of sensitivity (or recall) were for blocks C00-C97—malignant neoplasms (0.98), I60-I69—cerebrovascular diseases (0.94), and J09-J18—influenza and pneumonia (0.94). Considering the PPV (or precision), the highest values were for blocks C00-C97—malignant neoplasms (0.99), I60-I69—cerebrovascular diseases (0.95), and I20-I25—ischemic heart disease (0.94). The highest *F*_1_-scores were for blocks C00-C97—malignant neoplasms (0.99), I60-I69—cerebrovascular diseases (0.95), and J09-J18—influenza and pneumonia (0.94).

Specificity in all ICD-10 blocks was >0.99 for periods of excess mortality. The highest values of sensitivity (or recall) were for blocks C00-C97—malignant neoplasms (0.98), I60-I69—cerebrovascular diseases (0.94), and J09-J18—influenza and pneumonia (0.93). Considering the PPV (or precision), the highest values were for blocks C00-C97—malignant neoplasms (0.99), I60-I69—cerebrovascular diseases (0.95), and J09-J18—influenza and pneumonia (0.94). The highest *F*_1_-scores were for blocks C00-C97—malignant neoplasms (0.98), I60-I69—cerebrovascular diseases (0.95), and J09-J18—influenza and pneumonia (0.94).

Regarding the periods of severe excess mortality, with *Z* scores of >4 SDs, the specificity in all ICD-10 blocks was >0.98. The highest values of sensitivity (or recall) were for blocks C00-C97—malignant neoplasms (0.97), I60-I69—cerebrovascular diseases (0.93), and J09-J18—influenza and pneumonia (0.93). Considering the PPV (or precision), the highest values were for blocks C00-C97—malignant neoplasms (0.99), I60-I69—cerebrovascular diseases (0.96), and I20-I25—ischemic heart disease (0.95). The highest *F*_1_-scores were for blocks C00-C97—malignant neoplasms (0.98), I60-I69—cerebrovascular diseases (0.95), and I20-I25—ischemic heart diseases (0.94).

Specificity in all ICD-10 blocks was >0.99 for periods of extreme excess mortality (*z* score of >6 SDs). The highest values of sensitivity (or recall) were for blocks C00-C97—malignant neoplasms (0.97), I60-I69—cerebrovascular diseases (0.93), and J09-J18—influenza and pneumonia (0.93). Considering the PPV (or precision), the highest values were for blocks D10-D36—benign neoplasms (1.00); I80-I89—diseases of the veins, lymphatic vessels, and lymph nodes not elsewhere classified (1.00); and C00-C97—malignant neoplasms (0.99). The highest *F*_1_-scores were for blocks C00-C97—malignant neoplasms (0.98), I60-I69—cerebrovascular diseases (0.94), and J09-J18—influenza and pneumonia (0.94).

Table 5 presents AUTOCOD’s performance metrics for the weighted average of all the blocks analyzed. For sensitivity, PPV, and *F*_1_-score, there was a decrease of 0.01 from periods without excess mortality to periods with excess mortality, severe excess mortality (>4 SDs), and extreme excess mortality (>6 SDs).

Considering the differences between periods of excess mortality and periods without excess mortality, it is important to analyze which blocks had the biggest differences.

According to Table 6, the largest differences in the sensitivity of AUTOCOD between periods without excess mortality and periods of excess mortality were in block J00-J06—acute upper respiratory infections (0.34), J30-J39—other diseases of the upper respiratory tract (0.28), and I95-I99—other and unspecified disorders of the circulatory system (0.08). Regarding the difference in sensitivity between periods without excess mortality and periods of severe excess mortality (>4 SDs), the largest differences were in block J00-J06—acute upper respiratory infections (0.41), J85-J86—suppurative and necrotic conditions of the lower respiratory tract (0.23), J30-J39—other diseases of the upper respiratory tract (0.20), and I05-I09—chronic rheumatic heart diseases (−0.22). The largest differences in the sensitivity of AUTOCOD between periods without excess mortality and periods of extreme excess mortality (>6 SDs) were in blocks J00-J06—acute upper respiratory infections (0.41), J85-J86—suppurative and necrotic conditions of the lower respiratory tract (0.31), and I05-I09—chronic rheumatic heart diseases (−0.26).

Table 6 also shows the KLD between periods without excess mortality and periods of excess mortality. For 7 blocks, including C00-C97—malignant neoplasms and I60-I69—cerebrovascular diseases, the KLD was 0. Several blocks had values of KLD very close to 0, such as I20-I25—ischemic heart diseases and J09-J18—influenza and pneumonia. When comparing the difference between the KLD for the sensitivity of AUTOCOD for periods without excess mortality and periods of extreme excess mortality (*Z* score of >4 SDs), sensitivity had a KLD of 0 in 2 blocks: D37-D48—neoplasms of uncertain or unknown behavior and J95-J99—other diseases of the respiratory system. It also showed a KLD very close to 0 in blocks such as C00-C97—malignant neoplasms and I60-I69—cerebrovascular diseases. Regarding the KLD between periods without excess mortality and periods of extreme excess mortality (*Z* score of >6 SDs), the sensitivity had a KLD of 0 for I26-I28—pulmonary heart disease and diseases of pulmonary circulation and J40-J47—chronic lower respiratory diseases and a KLD very close to 0 for C00-C97—malignant neoplasm, I20-I25—ischemic heart diseases, and J09-J18—influenza and pneumonia. Some blocks, such as J00-J06—acute upper respiratory infections and J85-J86—suppurative and necrotic conditions of the lower respiratory tract, had a particularly high KLD for increasing mortality periods.

**Figure 3:**
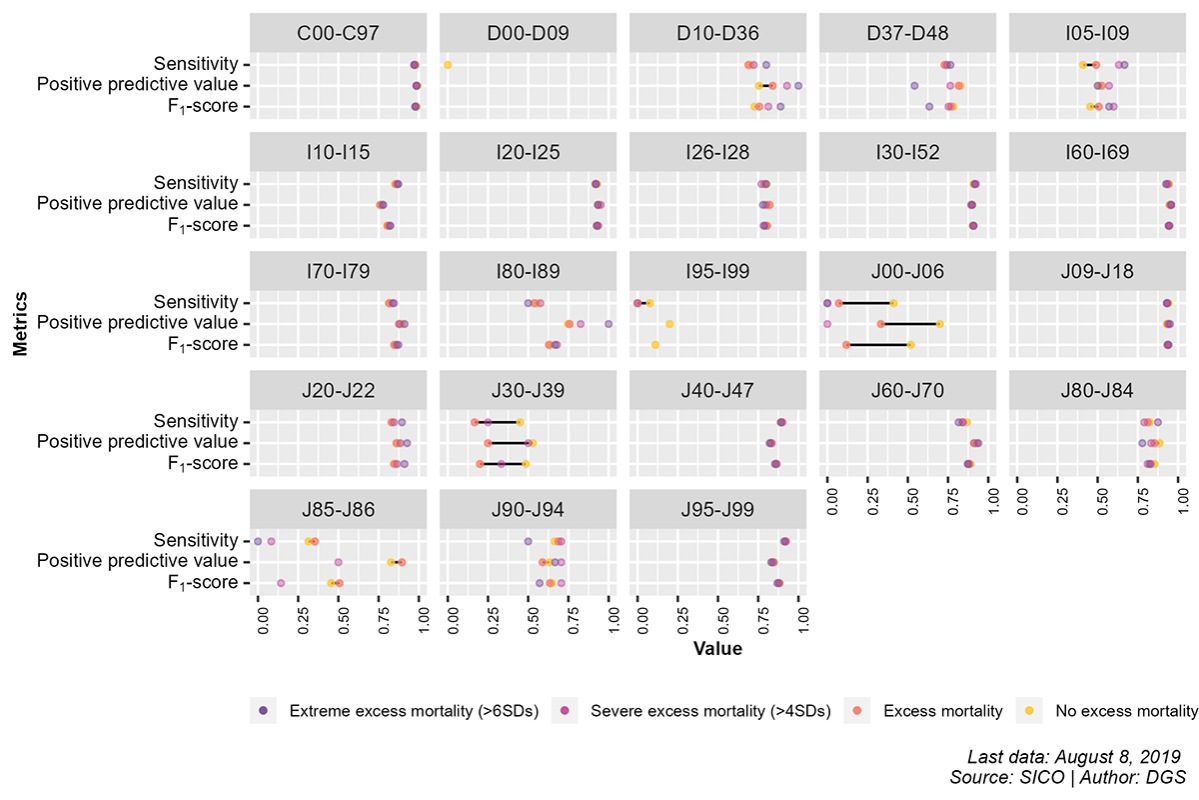
Comparison between performance metrics of AUTOCOD during periods of excess mortality and periods without excess mortality for International Statistical Classification of Diseases and Health-Related Problems, 10th Revision (ICD-10), blocks. DGS: Directorate-General of Health; SICO: Death Certificate Information System.

The differences in the performance measures of AUTOCOD among periods without excess mortality, with excess mortality, and with extreme excess mortality according to ICD-10 blocks are shown in Figure 3. Additional AUTOCOD performance comparisons between periods can be found in Multimedia Appendix 1 [17-19].

**Table 5:** Weighted averages of performance metrics for different periods for the International Statistical Classification of Diseases and Health-Related Problems, 10th Revision, block classification of AUTOCOD.

	Sensitivity (weighted average)	Specificity (weighted average)	Positive predictive value (weighted average)	*F*_1_-score (weighted average)
No excess mortality	0.94	0.99	0.94	0.94
Excess mortality	0.93	0.99	0.93	0.93
Severe excess mortality (>4 SDs)	0.93	0.99	0.93	0.93
Extreme excess mortality (>6 SDs)	0.93	0.99	0.93	0.93

**Table 6:** Comparison between sensitivity values of AUTOCOD depending on the period (total, excess mortality, or without excess mortality) by International Statistical Classification of Diseases and Health-Related Problems, 10th Revision, block.

Block	No excess mortality	Excess mortality	Difference (no excess mortality–excess mortality)	KLD^a^ (no excess mortality and excess mortality)	Severe excess mortality (>4 SDs)	Difference (>4 SDs–no excess mortality)	KLD (no excess mortality and >4 SDs)	Extreme excess mortality (>6 SDs)	Difference (>6 SDs–no excess mortality)	KLD (no excess mortality and >6 SDs)
C00-C97	0.98	0.98	<0.01	0.00	0.97	<0.01	0.01	0.97	<0.01	0.01
D00-D09	0.00	N/A^b^	N/A	N/A	N/A	N/A	N/A	N/A	N/A	N/A
D10-D36	0.70	0.69	0.01	0.01	0.72	−0.02	−0.02	0.80	−0.10	−0.09
D37-D48	0.74	0.73	0.01	0.01	0.74	0.00	0.00	0.77	−0.02	−0.02
I05-I09	0.41	0.49	−0.08	−0.07	0.63	−0.22	−0.18	0.67	−0.26	−0.20
I10-I15	0.85	0.86	−0.01	0.00	0.87	−0.02	−0.02	0.87	−0.02	−0.02
I20-I25	0.93	0.92	0.01	0.01	0.92	0.01	0.01	0.92	0.01	0.01
I26-I28	0.80	0.79	0.01	0.01	0.77	0.03	0.03	0.80	−<0.01	0.00
I30-I52	0.91	0.92	−0.01	−0.01	0.92	−0.02	−0.01	0.92	−0.01	−0.01
I60-I69	0.94	0.94	<0.01	0.00	0.93	0.01	0.01	0.93	0.02	0.02
I70-I79	0.82	0.82	<0.01	0.00	0.85	−0.03	−0.03	0.84	−0.02	−0.02
I80-I89	0.55	0.54	0.02	0.02	0.58	−0.02	−0.02	0.50	0.05	0.06
I95-I99	0.08	0.00	0.08	0.69	0.00	0.08	0.69	N/A	N/A	N/A
J00-J06	0.41	0.07	0.34	0.72	0.00	0.41	4.38	0.00	0.41	4.38
J09-J18	0.94	0.93	0.01	0.01	0.93	0.01	0.01	0.93	0.01	0.01
J20-J22	0.83	0.83	0.00	0.00	0.85	−0.01	−0.01	0.90	−0.06	−0.06
J30-J39	0.45	0.17	0.28	0.45	0.25	0.20	0.26	N/A	N/A	N/A
J40-J47	0.89	0.89	0.00	0.00	0.90	−0.01	−0.01	0.89	0.00	0.00
J60-J70	0.87	0.84	0.03	0.03	0.84	0.03	0.03	0.82	0.05	0.05
J80-J84	0.82	0.81	0.01	0.01	0.79	0.03	0.03	0.88	−0.05	−0.05
J85-J86	0.31	0.35	−0.04	−0.04	0.08	0.23	0.42	0.00	0.31	4.38
J90-J94	0.66	0.69	−0.02	−0.02	0.71	−0.04	−0.04	0.50	0.16	0.19
J95-J99	0.92	0.92	0.00	0.00	0.92	0.00	0.00	0.91	0.01	0.01

^a^KLD: Kullback-Leibler divergence.

^b^N/A: not applicable.

## Discussion

### Principal Findings

Continuous and systematic mortality data collection is crucial for monitoring the population’s health and complementing epidemiological studies. This national study is the first to demonstrate the robustness of deep neural networks in classifying primary causes of death even during periods of excess mortality, enabling cause-specific mortality surveillance, which is not widely performed worldwide. This study demonstrated a consistently good performance of AUTOCOD in different periods regardless of excess mortality rates. The results demonstrate the potential of AI algorithms to expedite disease classification and coding, making them a valuable tool for real-time surveillance, timely assessment of public health risks, and planification of responses. Proving that these algorithms can operate effectively despite external factors in different environments reinforces the case for their implementation.

AUTOCOD showed high sensitivity (≥0.75) in 10 chapters, with values of >0.90 for the 3 most common ones (chapter II—“Neoplasms,” chapter IX—“Diseases of the circulatory system,” and chapter X—“Diseases of the respiratory system,” which together account for 223,459/330,098, 67.69% of all human-codified causes of death). The weighted average of sensitivity in the ICD-10 chapter analysis showed no difference between periods without excess mortality and periods of excess mortality, a difference of 0.01 between periods without excess mortality and periods of severe excess mortality (>4 SDs), and a difference of 0.04 between periods without excess mortality and periods of extreme excess mortality (>6 SDs). Regarding the ICD-10 block analysis, it showed a difference of 0.01 for the weighted average of sensitivity between periods without excess mortality and periods of excess mortality between periods without excess mortality and periods of severe (at the >4 SD threshold) and between periods without excess mortality and periods of extreme excess mortality (at the >6 SD threshold).

In the different periods considered for the ICD-10 chapter analysis, AUTOCOD showed a consistently good performance, demonstrating a sensitivity (or recall), a PPV (or precision), and an *F*_1_-score as high as 0.88 for periods without excess mortality and periods of excess mortality and as low as 0.84 in periods of extreme excess mortality (>6 SDs). When we considered only the most common chapters (chapter II—“Neoplasms,” chapter IX—“Diseases of the circulatory system,” and chapter X—“Diseases of the respiratory system”), sensitivity ranged from 0.94 to 0.95 in chapter II, 0.91 in chapter IX, and 0.89 to 0.90 in chapter X in the different periods analyzed. The same happened with the PPV, which ranged from 0.96 to 0.98 in chapter II, 0.90 to 0.92 in chapter IX, and 0.83 to 0.86 in chapter X. Regarding the *F*_1_-score, the performance of AUTOCOD was 0.96 in chapter II, 0.91 in chapter IX, and 0.86 to 0.88 in chapter X. When we considered only the most common blocks—C00-C97 (malignant neoplasms), I60-I69 (cerebrovascular diseases), I30-I52 (other forms of heart disease), I20-I25 (ischemic heart diseases), and J09-J18 (influenza and pneumonia)—the sensitivity ranged from 0.91 to 0.98, the PPV ranged from 0.89 to 0.99, and the *F*_1_-score ranged from 0.90 to 0.99.

AUTOCOD presented high specificity and negative predictive values in all the analyses performed. This was expected as the number of true negatives was consistently much higher than that of true positives. This is not a characteristic of AUTOCOD itself but rather a result of our handling of the sample and our interpretation of the question as a classification problem with a one-versus-all solution. This method is widely used for multiple-output class classification problems. In our case, the individual ICD-10 chapters or blocks were handled as if they were in a binary model, thus assessing each class individually against all the other classes in the model.

It should be noted that chapter XVII (“Symptoms, signs, and abnormal clinical and laboratory findings not elsewhere specified”) consistently presented high performance metrics in AUTOCOD. This does not translate to a correct certification of the cause of death, but it could imply that, when human coders have difficulties classifying the cause of death, so does the AUTOCOD.

These results are aligned with those of previous studies using AUTOCOD [12,13] and, in general, with the literature on deep neural networks applied to the automatic classification of DCs [14,27,28]. Falissard et al [14] developed a deep neural network for automated coding of the underlying cause of death with a test accuracy of 0.978 (95% CI 0.977-0.979) and an *F*-measure value of 0.952 (95% CI 0.946-0.957) [27]. The proposed approach by Della Mea et al [28] for automated coding of causes of death had an accuracy of 0.990 (95% CI 0.990-0.991) and a macroaveraged accuracy and *F*_1_-score of 0.974 and 0.968, respectively. Similarly to our study, Della Mea et al [28] found that accuracy was low for chapters with rare causes of death and, therefore, rare causes of death could be ignored.

However, to the best of our knowledge, this is the first time that a deep neural network that classifies basic causes of death has been evaluated while comparing its performance across different time frames according to their excess mortality rates.

Automatic classification of DCs relies on natural language processing (NLP) techniques and algorithms. NLP can translate free text written by the physician who certified the death into classification codes based on the ICD-10. However, this process depends on the text quality of the analyzed DCs. By text quality, we mean how successfully we can automatically classify, retrieve, or extract information from them [29]. Thus, text quality does not involve a single aspect but combines numerous criteria, including spelling, grammar, organization, informative nature, and page layout [30]. Extracting these attributes can become problematic in low-quality texts (poor grammar, many abbreviations, and short sentences). This is a known problem in medical and clinical texts such as patient records or DCs [30]. The performance of systems that rely on attributes of text quality, such as NLP, affects the overall performance of the algorithms—a text of bad quality may result in poor-quality prediction results. To overcome this limitation, after the development AUTOCOD, a processing layer has been added to the neural network that has the ability to always read words in text fields as the closest word the model knows (eg, for the word *Alzheimer*, it currently identifies >25 ways of misspelling it). Therefore, this processing layer can help minimize text field errors or abbreviations in periods of excess mortality [31-33].

Our results suggest that, even in periods of excess, severe, and extreme excess mortality when the volume of deaths and the pressure on health services might increase, with a consequent impact on physicians that certify deaths and a potential impact on the quality of the text in the DC, AUTOCOD’s performance remains unhindered. It is important to consider analyzing the linguistic properties of the DC, such as variations in text size and the number of fields filled in by physicians, in future work.

### Limitations

An important limitation of this study is that the human coders had access to the automatic classification of the DC by AUTOCOD, meaning that the gold standard we used in this research might be biased by the same algorithm we were trying to evaluate. However, this implementation only entered production on July 26, 2019, meaning that manual classification was unbiased for most of the data sets used in this study.

In addition, there is the matter of ICD-10 code ambiguity. This is a known limitation of the ICD-10 for human coders and automatic algorithms of classification that the sometimes discrete differences between codes for similar causes of death can explain. This might explain the difference in sensitivity between, for example, respiratory blocks such as J00-J06 (acute upper respiratory infections) and J09-J18 (influenza and pneumonia), with the latter presenting a less ambiguous cause of death when compared with the former both for human classification and automatic classification. These unspecified codes are not necessarily an error rate but an indicator of the completeness of clinical information of DCs in which sufficient clinical information is not known or available to assign a more specific code. In the case of human coders, it is common that they look for more clinical information in electronic health records. However, AUTOCOD is restricted to the information included in the DC. This stresses the importance of a well-filled and detailed DC by the physician that certifies the death even in periods of excess mortality.

Routinely, racial and ethnic or socioeconomic groups are not collected in the DC. Although other proxies of social vulnerability can be used, such as the municipality of residence, the focus of this research was not the study of differences in subgroups, making this an important next step of investigation.

The human coders that we set as our ground truth were not mistake free. Current research puts the reliability of human coders at approximately 70% to 89% (reliability is a measure for calculating agreement between coders and the consistency of each coder individually) [34]. These performance scores can be in part explained by the use of different codes for similar diseases. Moreover, the DGS has had a range of human coders that varies in number, typically from 4 to 6, and in experience in classifying causes of death. This may also affect the reliability and accuracy of the ground-truth labels we used in this study. Only 1 human coder classifies each DC, and the DGS regularly conducts an in-house auditing process in which 2 human coders check for internal reliability by classifying a small sample of DCs.

Another possible limitation, known in the field of AI algorithms, is the generalization of our results to other countries [35]. This question of model transferability requires further study. However, we feel confident that our results can be generalized to other algorithms that rely on NLP for automatic classification without a profound impact on the model’s performance even in periods of excess mortality.

### Strengths

In Portugal, Law 15/2012 of April 3, 2012, established the SICO, a mortality information system based on the electronic registration of DCs [36]. Since then, SICO has become a widespread tool used by physicians nationally. Therefore, it is a well-established source of data and information related to mortality and an international example of the timeliness of mortality statistics [3].

AUTOCOD was built based on the already disseminated existence of DCs in electronic format and has since been validated as an essential tool for the automatic assignment of ICD-10 codes for causes of death [13]. However, this validation never considered differences in periods that might affect the quality of the DC and, consequently, the performance of AUTOCOD. The method we used for evaluating the performance of AUTOCOD during periods of excess mortality, severe excess mortality, and extreme excess mortality is a known method for comparison of the performance of a given index test with a given ground truth or gold standard, making a case for the importance of evaluating algorithms and models in different periods and in the ever-changing environment that might affect the overall performance of the models.

Although the current use of AUTOCOD is limited to supporting human coders, the research findings suggest a compelling case for enhancing the algorithms used for the automated classification of causes of death. In a completed DC, AUTOCOD can be used to accurately classify basic causes of death in real time even in periods of excess mortality, attesting that deep neural networks are robust to eventual changes in the underlying quality of the text. Furthermore, by defining a baseline from the past (and Portugal has digital DC data going back to 2014), we can detect in real time, with high sensitivity, changes in mortality and periods of excess mortality without the need to wait for human classification of cause of death, especially for the more common and less ambiguous causes of death. Finally, with this algorithm, we can use our data to predict excess deaths that rely on seasonality, such as influenza and pneumonia.

### Implications of Our Work

Our work makes a case for using AUTOCOD for real-time mortality surveillance by ICD-10 codes. It can be further validated by other countries wishing to train their neural networks for medical and clinical text classification. Our research also makes a case for auditing, evaluating, and consistently monitoring AI algorithms to identify potential barriers, strengths, and opportunities [37].

As the AUTOCOD algorithm is robust, it can be used to classify the underlying causes of death in periods of excess mortality with no need to wait for manual coding, which allows for adequate real-time cause-specific mortality surveillance, timely assessment of risks to public health, and definition of priorities and planification of responses in both periods with and without excess mortality. This cause-specific mortality surveillance in real time is not carried out widely worldwide and might benefit from further investigation and real-world intervention. This investigation is a step forward in Portugal for the widespread use of the classification of specific causes of death by the AUTOCOD, with renewed confidence in its results regardless of the presence of excess mortality, and for the implementation of targeted public health interventions and practices.

Further investigations should be carried out, such as a comparison of AUTOCOD with other automated coding systems and a new evaluation of the behavior of AUTOCOD during periods of excess mortality caused by the COVID-19 pandemic, including retraining the algorithm with the new codes for COVID-19 that were not present in the ICD-10 when AUTOCOD was built [14,16,28]. To strengthen coding practices, conducting a reliability study among coders at the DGS would also be important.

### Conclusions

This study makes the case for deep neural networks as powerful tools for automatically classifying primary causes of death according to the ICD-10 even during periods of excess mortality. Our work could potentially further the use of deep neural networks to facilitate automatic clinical codification, such as of diseases, medical procedures, or DCs. In addition, it may serve as a staple for the real-time monitoring and surveillance of public health threats and problems, allowing for timely action. More broadly, this study highlights the importance of AI algorithms as an advisory tool for public health policies and measures.

## Appendix

Multimedia Appendix 1 Description of the data set and AUTOCOD’s performance in different periods of excess mortality for both International Statistical Classification of Diseases and Health-Related Problems, 10th Revision, chapters and blocks.

## Abbreviations

AI: artificial intelligence
DC: death certificate
DGS: Directorate-General of Health
EuroMOMO: European mortality monitoring project
ICD-10: International Statistical Classification of Diseases and Health-Related Problems, 10th Revision
KLD: Kullback-Leibler divergence
NLP: natural language processing
PPV: positive predictive value
SICO: Death Certificate Information System

## Data Availability

Data from the Death Certificate Information System and related analyses are available for research purposes under the conditions foreseen in Law 15/2012.
