# Supplementary material for: Real-Time Classification of Causes of Death Using AI: Sensitivity Analysis
Source: JMIR AI. 2023 Nov 22;2:e40965. doi: 10.2196/40965 (PMC11041420; doi:10.2196/40965)
Supplement: Multimedia Appendix 1 [file ai_v2i1e40965_app1.pdf]

Supplementary Material

Real-Time Classification of Causes of Death Using AI: Sensitivity Analysis

Authors:

Patrícia Pita Ferreira[1,2,3], MD; Diogo Godinho Simões[1,4], MD; Constança Pinto de Carvalho[1,5], MD; Francisco Duarte[6]; Eugénia Fernandes[1], PhD; Pedro Casaca[1], MD; José Loff[7], MSc; Ana Paula Soares[1], MPH; Maria João Albuquerque[1], BSc; Pedro Pinto-Leite[1], MD; André Peralta-Santos[1,8,9], MD, MPH, PhD

[1] Direção de Serviços de Informação e Análise, Direção-Geral da Saúde, Lisbon, Portugal

[2] Unidade de Saúde Pública Zé Povinho, Agrupamento de Centros de Saúde do Oeste Norte, Administração Regional de Saúde de Lisboa e Vale do Tejo, Caldas da Rainha, Portugal

[3] NOVA National School of Public Health, Universidade NOVA de Lisboa, Lisbon, Portugal

[4] Unidade de Saúde Pública Higeia, Agrupamento de Centros de Saúde de Almada-Seixal, Administração Regional de Saúde de Lisboa e Vale do Tejo, Almada, Portugal

[5] Unidade de Saúde Pública do Litoral Alentejano, Unidade Local de Saúde do Litoral Alentejano, Administração Regional de Saúde do Alentejo, Santiago do Cacém, Portugal

[6] Lisbon, Portugal

[7] phiStat – Statistical Consulting, Lisbon, Portugal

[8] NOVA National School of Public Health, Public Health Research Centre, Universidade NOVA de Lisboa, Lisbon, Portugal

[9] Comprehensive Health Research Centre, Universidade NOVA de Lisboa, Lisbon, Portugal

Address for correspondence: ppita.ferreira@gmail.com

Contents

Functions 3

Function to implement Westegard [17] rules for the definition of excess mortality periods 3

Function to implement Westegard rules for the definition of severe excess mortality periods (>4 SDs) 4

Function to implement Westegard rules for the definition of extreme excess mortality periods (>6 SDs) 5

Formula to calculate the weighted-average of performance metrics 6

Performance metrics for classification algorithms 7

ICD-10 chapter analysis 8

Performance metrics – global overview 8

All periods 9

Periods of excess mortality 17

Periods without excess mortality 23

Periods with severe excess mortality (>4 SDs) 29

Periods with extreme excess mortality (>6 SDs) 32

Difference between excess mortality periods and periods without excess mortality 35

ICD-10 block analysis 41

Performance metrics – global overview 41

All periods 43

Periods of excess mortality 50

Periods without excess mortality 53

Periods with severe excess mortality (>4 SDs) 56

Periods with extreme excess mortality (>6 SDs) 59

Difference between excess mortality periods and periods without excess mortality 61

Supplementary Material

Functions

Function to implement Westegard [17] rules for the definition of excess mortality periods

```
f.westgard <- function(z) {
  a95 <- z > qnorm(1 - 0.05/2)
  a99 <- z > qnorm(1 - 0.01/2)
  turn_on <- a99 | (a95 & c(tail(a95, -1), NA)) | (a95 & c(FALSE, head(a95, -1)))
  turn_off <- (!a95 & !c(tail(a95, -1), NA)) | (!a95 & !c(NA, head(a95, -1)))

  state <- logical(length(turn_on))
  state[1] <- turn_on[1]
  for (i in 2:(length(state))) {
    if (is.na(turn_on[i]) | is.na(turn_off[i])) {
      state[i] <- NA
    } else if (state[i - 1] & turn_off[i]) {
      state[i] <- FALSE
    } else if ( !state[i - 1] & turn_on[i]) {
     state[i] <- TRUE
    } else {
     state[i] <- state[i - 1]
    }
  }
  return(state)
}
```

Whereas z is the z-score.

Function to implement Westegard rules for the definition of severe excess mortality periods (>4 SDs)

```
f.westgard <- function(z) {
  plus4 <- z > 4
  turn_on <- (plus4 & c(tail(plus4, -1), NA)) | (plus4 & c(FALSE, head(plus4, -1)))
  turn_off <- (!plus4 & !c(tail(plus4, -1), NA)) | (!plus4 & !c(NA, head(plus4, -1)))

  state <- logical(length(turn_on))
  state[1] <- turn_on[1]
  for (i in 2:(length(state))) {
   if (is.na(turn_on[i]) | is.na(turn_off[i])) {
     state[i] <- NA
   } else if (state[i - 1] & turn_off[i]) {
     state[i] <- FALSE
   } else if ( !state[i - 1] & turn_on[i]) {
    state[i] <- TRUE
   } else {
    state[i] <- state[i - 1]
   }
  }
  return(state)
 }
```

Whereas z is the z-score.

Function to implement Westegard rules for the definition of extreme excess mortality periods (>6 SDs)

```
f.westgard <- function(z) {
 plus6 <- z > 6
 turn_on <- (plus6 & c(tail(plus6, -1), NA)) | (plus6 & c(FALSE, head(plus6, -1)))
 turn_off <- (!plus6 & !c(tail(plus6, -1), NA)) | (!plus6 & !c(NA, head(plus6, -1)))

 state <- logical(length(turn_on))
 state[1] <- turn_on[1]
 for (i in 2:(length(state))) {
   if (is.na(turn_on[i]) | is.na(turn_off[i])) {
     state[i] <- NA
   } else if (state[i - 1] & turn_off[i]) {
     state[i] <- FALSE
   } else if ( !state[i - 1] & turn_on[i]) {
    state[i] <- TRUE
   } else {
    state[i] <- state[i - 1]
   }
 }
 return(state)
}
```

Whereas z is the z-score.

Formula to calculate the weighted-average of performance metrics

$$\sum_{i=1}^{N} \boldsymbol{PF}_{\boldsymbol{class\ i}} \times \boldsymbol{W}_{\boldsymbol{class\ i}}$$

Where **PF** stands for the performance metric for each of the classes evaluated; **class** stands either for the ICD-10 chapters or ICD-10 blocks;

And **W** stands for the number of true labels of each class, in our case, it stands for the **detection prevalence** calculated by the package “caret” [18, 19]

Performance metrics for classification algorithms

Supplementary Table 1 - Performance metrics calculated by the "confusionMatrix" function of the "caret" pacakage

		Human Coders (GOLD-STANDARD)	
		Event	No Event
AUTOCOD	Event	True Positives (TP)	False Positives (FP)
	No Event	False Negatives (FN)	True Negatives (TN)
		Sensitivity / Rate of TP $\frac{TP}{TP+FN}$	Specificity $\frac{TN}{TN+FP}$
		Prevalence $\frac{TP+FN}{TP+FP+FN+TN}$	
		Positive Predictive Value $\frac{sensitivity \times prevalence}{((sensitivity \times prevalence) + (1 - specificity) \times (1 - prevalence))}$	
		Negative Predictive Value $\frac{specificity \times (1 - prevalence)}{((1 - sensitivity) \times prevalence) + (specificity) \times (1 - prevalence))}$	
		Balanced Accuracy $(sensitivity + specificity)/2$	
		Detection Rate $\frac{TP}{TP+FN+FP+TN}$	Detection Prevalence $\frac{TP+FP}{TP+FN+FP+TN}$
		Precision $\frac{TP}{TP+FP}$	Recall $\frac{TP}{TP+FN}$
		F1-Score $\frac{(1+\beta^2) \times precision \times recall}{(\beta^2 \times precision) + recall}$	

ICD-10 chapter analysis

Performance metrics – global overview

Supplementary Table 2 - Performance Metrics of AUTOCOD, by chapter, for all the periods analyzed

	I				II				III				IV				V			
	No Excess	Excess Mort.	Severe >4SD	Extreme >6SD	No Excess	Excess Mort.	Severe >4SD	Extreme >6SD	No Excess	Excess Mort.	Severe >4SD	Extreme >6SD	No Excess	Excess Mort.	Severe >4SD	Extreme >6SD	No Excess	Excess Mort.	Severe >4SD	Extreme >6SD
Sen	0.67	0.67	0.67	0.65	0.95	0.95	0.94	0.95	0.57	0.55	0.58	0.41	0.81	0.81	0.81	0.82	0.77	0.78	0.78	0.77
Spe	0.99	0.99	0.99	0.99	0.99	0.99	0.99	0.99	1.00	1.00	1.00	1.00	0.98	0.98	0.98	0.98	0.99	0.99	0.99	0.99
PPV	0.61	0.64	0.65	0.62	0.98	0.97	0.97	0.96	0.46	0.51	0.53	0.36	0.69	0.69	0.69	0.69	0.82	0.80	0.80	0.83
NPV	0.99	0.99	0.99	0.99	0.98	0.98	0.99	0.99	1.00	1.00	1.00	1.00	0.99	0.99	0.99	0.99	0.99	0.99	0.99	0.99
F1	0.64	0.65	0.66	0.63	0.96	0.96	0.96	0.96	0.51	0.53	0.55	0.38	0.74	0.74	0.74	0.75	0.80	0.79	0.79	0.80
Pre	0.02	0.02	0.02	0.02	0.27	0.23	0.20	0.19	0.00	0.00	0.01	0.00	0.05	0.05	0.05	0.05	0.04	0.04	0.04	0.05
DR	0.01	0.01	0.01	0.01	0.26	0.21	0.19	0.18	0.00	0.00	0.00	0.00	0.04	0.04	0.04	0.04	0.03	0.03	0.03	0.04
DP	0.02	0.02	0.02	0.02	0.27	0.22	0.20	0.19	0.01	0.01	0.01	0.00	0.06	0.06	0.06	0.06	0.04	0.04	0.04	0.04
BA	0.83	0.83	0.83	0.82	0.97	0.97	0.97	0.97	0.79	0.78	0.79	0.70	0.89	0.90	0.89	0.90	0.88	0.89	0.88	0.88

	VI				VII				VIII				IX				X			
	No Excess	Excess Mort.	Severe >4SD	Extreme >6SD	No Excess	Excess Mort.	Severe >4SD	Extreme >6SD	No Excess	Excess Mort.	Severe >4SD	Extreme >6SD	No Excess	Excess Mort.	Severe >4SD	Extreme >6SD	No Excess	Excess Mort.	Severe >4SD	Extreme >6SD
Sen	0.79	0.80	0.79	0.79	0.00	0.00	NA	NA	0.18	0.25	0.40	NA	0.91	0.91	0.91	0.91	0.90	0.89	0.90	0.89
Spe	1.00	1.00	1.00	1.00	1.00	1.00	1.00	1.00	1.00	1.00	1.00	1.00	0.97	0.96	0.96	0.96	0.98	0.97	0.97	0.97
PPV	0.86	0.86	0.85	0.88	NaN	NaN	NA	NA	0.67	0.67	0.67	NA	0.92	0.91	0.91	0.90	0.83	0.85	0.86	0.86
NPV	0.99	0.99	0.99	0.99	1.00	1.00	NA	NA	1.00	1.00	1.00	NA	0.96	0.96	0.96	0.96	0.99	0.98	0.98	0.98
F1	0.82	0.83	0.82	0.83	NA	NA	NA	NA	0.29	0.36	0.50	NA	0.91	0.91	0.91	0.91	0.86	0.87	0.88	0.88
Pre	0.04	0.04	0.04	0.04	0.00	0.00	0.00	0.00	0.00	0.00	0.00	0.00	0.29	0.30	0.31	0.31	0.11	0.14	0.16	0.16
DR	0.03	0.03	0.03	0.03	0.00	0.00	0.00	0.00	0.00	0.00	0.00	0.00	0.27	0.28	0.28	0.28	0.10	0.13	0.14	0.15
DP	0.03	0.04	0.03	0.04	0.00	0.00	0.00	0.00	0.00	0.00	0.00	0.00	0.29	0.30	0.31	0.31	0.12	0.15	0.16	0.17
BA	0.90	0.90	0.89	0.89	0.50	0.50	NA	NA	0.59	0.63	0.70	NA	0.94	0.94	0.93	0.94	0.94	0.93	0.93	0.93

	XI				XII				XIII				XIV				XV			
	No Excess	Excess Mort.	Severe >4SD	Extreme >6SD	No Excess	Excess Mort.	Severe >4SD	Extreme >6SD	No Excess	Excess Mort.	Severe >4SD	Extreme >6SD	No Excess	Excess Mort.	Severe >4SD	Extreme >6SD	No Excess	Excess Mort.	Severe >4SD	Extreme >6SD
Sen	0.80	0.79	0.76	0.76	0.28	0.35	0.40	0.20	0.42	0.42	0.42	0.32	0.76	0.76	0.76	0.74	0.00	0.00	0.00	0.00
Spe	0.99	0.99	0.99	1.00	1.00	1.00	1.00	1.00	1.00	1.00	1.00	1.00	0.99	0.99	0.99	0.99	1.00	1.00	1.00	1.00
PPV	0.85	0.85	0.83	0.85	0.47	0.56	0.54	0.33	0.61	0.61	0.56	0.50	0.76	0.75	0.76	0.76	NaN	NaN	NA	NA
NPV	0.99	0.99	0.99	0.99	1.00	1.00	1.00	1.00	1.00	1.00	1.00	1.00	0.99	0.99	0.99	0.99	1.00	1.00	1.00	1.00
F1	0.83	0.82	0.79	0.80	0.35	0.43	0.46	0.25	0.49	0.50	0.48	0.39	0.76	0.75	0.76	0.75	NA	NA	NA	NA
Pre	0.05	0.04	0.04	0.04	0.00	0.00	0.00	0.00	0.00	0.00	0.00	0.00	0.03	0.03	0.03	0.03	0.00	0.00	0.00	0.00
DR	0.04	0.03	0.03	0.03	0.00	0.00	0.00	0.00	0.00	0.00	0.00	0.00	0.02	0.02	0.02	0.02	0.00	0.00	0.00	0.00
DP	0.04	0.04	0.04	0.03	0.00	0.00	0.00	0.00	0.00	0.00	0.00	0.00	0.03	0.03	0.03	0.03	0.00	0.00	0.00	0.00
BA	0.90	0.89	0.88	0.88	0.64	0.68	0.70	0.60	0.71	0.71	0.71	0.66	0.88	0.87	0.88	0.87	0.50	0.50	0.50	0.50

	XVI				XVII				XVIII				XIX				XX			
	No Excess	Excess Mort.	Severe >4SD	Extreme >6SD	No Excess	Excess Mort.	Severe >4SD	Extreme >6SD	No Excess	Excess Mort.	Severe >4SD	Extreme >6SD	No Excess	Excess Mort.	Severe >4SD	Extreme >6SD	No Excess	Excess Mort.	Severe >4SD	Extreme >6SD
Sen	0.07	0.00	0.00	NA	0.39	0.34	0.32	0.20	0.93	0.93	0.92	0.90	NA	0.00	0.00	NA	0.79	0.76	0.75	0.00
Spe	1.00	1.00	1.00	1.00	1.00	1.00	1.00	1.00	0.99	0.99	0.99	0.99	NA	1.00	1.00	1.00	1.00	1.00	1.00	0.00
PPV	1.00	NaN	NA	NA	0.76	0.71	0.71	0.67	0.88	0.88	0.88	0.88	NA	NaN	NA	NA	0.88	0.88	0.87	0.00
NPV	1.00	1.00	1.00	NA	1.00	1.00	1.00	1.00	1.00	1.00	1.00	0.99	NA	1.00	1.00	NA	0.99	0.99	0.99	0.00
F1	0.12	NA	NA	NA	0.51	0.46	0.44	0.31	0.90	0.90	0.90	0.89	NA	NA	NA	NA	0.83	0.82	0.81	0.00
Pre	0.00	0.00	0.00	0.00	0.00	0.00	0.00	0.00	0.05	0.06	0.06	0.07	NA	0.00	0.00	0.00	0.04	0.04	0.04	0.00
DR	0.00	0.00	0.00	0.00	0.00	0.00	0.00	0.00	0.04	0.05	0.06	0.06	NA	0.00	0.00	0.00	0.04	0.03	0.03	0.00
DP	0.00	0.00	0.00	0.00	0.00	0.00	0.00	0.00	0.05	0.06	0.06	0.07	NA	0.00	0.00	0.00	0.04	0.03	0.04	0.00
BA	0.53	0.50	0.50	NA	0.69	0.67	0.66	0.60	0.96	0.96	0.96	0.94	NA	0.50	0.50	NA	0.89	0.88	0.87	0.00

Caption: Sen – Sensitivity; Spe – Specificity; PPV – Positive Predictive Value; NPB – Negative Predictive Value; F1 – F1-score; Pre – Prevalence; DR – Detection Rate; DP – Detection Prevalence; BA – Balanced Accuracy; I - Certain infectious and parasitic diseases; II - Neoplasms; III - Diseases of the blood and blood-forming organs and certain disorders involving the immune system; IV - Endocrine, nutritional and metabolic diseases; V - Mental and behavioural disorders; VI - Diseases of the nervous system; VII - Diseases of the eye and adnexa; VIII - Diseases of the ear and mastoid process; IX - Diseases of the circulatory system; X - Diseases of the respiratory system; XI - Diseases of the digestive system; XII - Diseases of the skin and subcutaneous tissue; XIII - Diseases of the musculoskeletal system and connective tissue; XIV - Diseases of the genitourinary system; XV - Pregnancy, childbirth and the puerperium; XVI - Certain conditions originating in the perinatal period; XVII - Congenital malformations, deformations and chromosomal abnormalities; XVIII - Symptoms, signs and abnormal clinical and laboratory findings, not elsewhere specified; XIX - Injury, poisoning and certain other consequences of external causes; XX - External causes of morbidity and mortality; NA – Missing values; NaN – not possible to calculate

Supplementary Table 3 - Average for performance metrics for different periods, for ICD-10's chapter classification of AUTOCOD

		Sensitivity	Specificity	Pos Pred Value	Neg Pred Value	F1 score	Prevalence	Detection Rate	Detection Prevalence	Balanced Accuracy
**Weighted-averaged**	No Excess Mort.	0.88	0.98	0.88	0.98	0.88	0.18	0.17	0.18	0.93
	Excess Mort.	0.88	0.98	0.88	0.98	0.88	0.18	0.16	0.18	0.93
	Severe >4 SD	0.87	0.98	0.87	0.98	0.87	0.18	0.16	0.18	0.93
	Extreme >6 SD	0.85	0.94	0.84	0.94	0.84	0.17	0.16	0.17	0.89

All periods

Supplementary Table 4 – Distribution of death certificates in the dataset, according to source of classification

Source	N	%
**AUTOCOD**	330 098	50.00
**DGS**	330 098	50.00
**Total**	**660 196**	**100.00**

Supplementary Table 5 - Distribution of death certificates in the dataset, according to ICD-10 chapter (descendent order)

Chapter	Chapter Description	N	%
**IX**	Diseases of the circulatory system	97 420	29.51
**II**	Neoplasms	85 837	26.00
**X**	Diseases of the respiratory system	40 202	12.18
**IV**	Endocrine, nutritional and metabolic diseases	16 430	4.98
**XVIII**	Symptoms, signs and abnormal clinical and laboratory findings, not elsewhere specified	16 269	4.93
**XI**	Diseases of the digestive system	14 892	4.51
**XX**	External causes of morbidity and mortality	14 128	4.28
**V**	Mental and behavioural disorders	12 742	3.86
**VI**	Diseases of the nervous system	11 810	3.58
**XIV**	Diseases of the genitourinary system	10 277	3.11
**I**	Certain infectious and parasitic diseases	6 156	1.86
**XIII**	Diseases of the musculoskeletal system and connective tissue	1 397	0.42
**III**	Diseases of the blood and blood-forming organs and certain disorders involving the immune system	1 334	0.40
**XII**	Diseases of the skin and subcutaneous tissue	583	0.18
**XVII**	Congenital malformations, deformations and chromosomal abnormalities	494	0.15
**XVI**	Certain conditions originating in the perinatal period	58	0.02
**XV**	Pregnancy, childbirth and the puerperium	35	0.01
**VIII**	Diseases of the ear and mastoid process	30	0.01
**VII**	Diseases of the eye and adnexa	2	0.00
**XIX**	Injury, poisoning and certain other consequences of external causes	2	0.00
**Total**		**330 098**	**100.00**

Supplementary Table 6 - Distribution of death certificates according to year of death

Year of Death	N	%
**2016**	109 785	33.26
**2017**	105 534	31.97
**2018**	107 467	32.56
**2019**	7 312	2.22
**Total**	**330 098**	**100.0**

Supplementary Table 7 - Distribution of death certificates, according to year of death and ICD-10 chapter

Year of death	Chapter	Chapter Description	N	%
**2016**	IX	Diseases of the circulatory system	32 765	9.93
**2018**	IX	Diseases of the circulatory system	31 262	9.47
**2017**	IX	Diseases of the circulatory system	31 111	9.42
**2018**	II	Neoplasms	28 293	8.57
**2016**	II	Neoplasms	27 984	8.48
**2017**	II	Neoplasms	27 869	8.44
**2016**	X	Diseases of the respiratory system	13 470	4.08
**2018**	X	Diseases of the respiratory system	13 114	3.97
**2017**	X	Diseases of the respiratory system	12 653	3.83
**2016**	XVIII	Symptoms, signs and abnormal clinical and laboratory findings, not elsewhere specified	5 916	1.79
**2016**	IV	Endocrine, nutritional and metabolic diseases	5 584	1.69
**2018**	IV	Endocrine, nutritional and metabolic diseases	5 310	1.61
**2017**	IV	Endocrine, nutritional and metabolic diseases	5 184	1.57
**2017**	XVIII	Symptoms, signs and abnormal clinical and laboratory findings, not elsewhere specified	5 060	1.53
**2016**	XI	Diseases of the digestive system	4 979	1.51
**2018**	XVIII	Symptoms, signs and abnormal clinical and laboratory findings, not elsewhere specified	4 943	1.50
**2017**	XI	Diseases of the digestive system	4 871	1.48
**2016**	XX	External causes of morbidity and mortality	4 832	1.46
**2018**	V	Mental and behavioural disorders	4 737	1.44
**2018**	XI	Diseases of the digestive system	4 721	1.43
**2017**	XX	External causes of morbidity and mortality	4 585	1.39
**2018**	XX	External causes of morbidity and mortality	4 431	1.34
**2018**	VI	Diseases of the nervous system	3 969	1.20
**2017**	V	Mental and behavioural disorders	3 947	1.20
**2016**	VI	Diseases of the nervous system	3 841	1.16
**2017**	VI	Diseases of the nervous system	3 748	1.14
**2016**	V	Mental and behavioural disorders	3 688	1.12
**2016**	XIV	Diseases of the genitourinary system	3 437	1.04
**2018**	XIV	Diseases of the genitourinary system	3 334	1.01
**2017**	XIV	Diseases of the genitourinary system	3 306	1.00
**2019**	IX	Diseases of the circulatory system	2 282	0.69
**2017**	I	Certain infectious and parasitic diseases	2 012	0.61
**2018**	I	Certain infectious and parasitic diseases	2 007	0.61
**2016**	I	Certain infectious and parasitic diseases	2 003	0.61
**2019**	II	Neoplasms	1 691	0.51
**2019**	X	Diseases of the respiratory system	965	0.29
**2018**	XIII	Diseases of the musculoskeletal system and connective tissue	479	0.15
**2016**	XIII	Diseases of the musculoskeletal system and connective tissue	458	0.14
**2017**	III	Diseases of the blood and blood-forming organs and certain disorders involving the immune system	452	0.14
**2016**	III	Diseases of the blood and blood-forming organs and certain disorders involving the immune system	438	0.13
**2017**	XIII	Diseases of the musculoskeletal system and connective tissue	426	0.13
**2018**	III	Diseases of the blood and blood-forming organs and certain disorders involving the immune system	417	0.13
**2019**	V	Mental and behavioural disorders	370	0.11

Supplementary Table 8 - Distribution of death certificates, according to year of death and ICD-10 chapter (cont.)

Year of death	Chapter	Chapter Description	N	%
**2019**	IV	Endocrine, nutritional and metabolic diseases	352	0.11
**2019**	XVIII	Symptoms, signs and abnormal clinical and laboratory findings, not elsewhere specified	350	0.11
**2019**	XI	Diseases of the digestive system	321	0.10
**2019**	XX	External causes of morbidity and mortality	280	0.08
**2019**	VI	Diseases of the nervous system	252	0.08
**2018**	XII	Diseases of the skin and subcutaneous tissue	214	0.06
**2016**	XII	Diseases of the skin and subcutaneous tissue	209	0.06
**2019**	XIV	Diseases of the genitourinary system	200	0.06
**2018**	XVII	Congenital malformations, deformations and chromosomal abnormalities	185	0.06
**2017**	XVII	Congenital malformations, deformations and chromosomal abnormalities	152	0.05
**2016**	XVII	Congenital malformations, deformations and chromosomal abnormalities	144	0.04
**2019**	I	Certain infectious and parasitic diseases	134	0.04
**2017**	XII	Diseases of the skin and subcutaneous tissue	125	0.04
**2019**	XII	Diseases of the skin and subcutaneous tissue	35	0.01
**2019**	XIII	Diseases of the musculoskeletal system and connective tissue	34	0.01
**2018**	XVI	Certain conditions originating in the perinatal period	27	0.01
**2019**	III	Diseases of the blood and blood-forming organs and certain disorders involving the immune system	27	0.01
**2016**	XVI	Certain conditions originating in the perinatal period	21	0.01
**2019**	XVII	Congenital malformations, deformations and chromosomal abnormalities	13	0.00
**2017**	VIII	Diseases of the ear and mastoid process	12	0.00
**2018**	XV	Pregnancy, childbirth and the puerperium	12	0.00
**2017**	XV	Pregnancy, childbirth and the puerperium	11	0.00
**2018**	VIII	Diseases of the ear and mastoid process	11	0.00
**2017**	XVI	Certain conditions originating in the perinatal period	10	0.00
**2016**	VIII	Diseases of the ear and mastoid process	7	0.00
**2016**	XV	Pregnancy, childbirth and the puerperium	7	0.00
**2019**	XV	Pregnancy, childbirth and the puerperium	5	0.00
**2016**	VII	Diseases of the eye and adnexa	2	0.00
**2018**	XIX	Injury, poisoning and certain other consequences of external causes	1	0.00
**2019**	XIX	Injury, poisoning and certain other consequences of external causes	1	0.00
**Total**			**330 098**	**100.00**

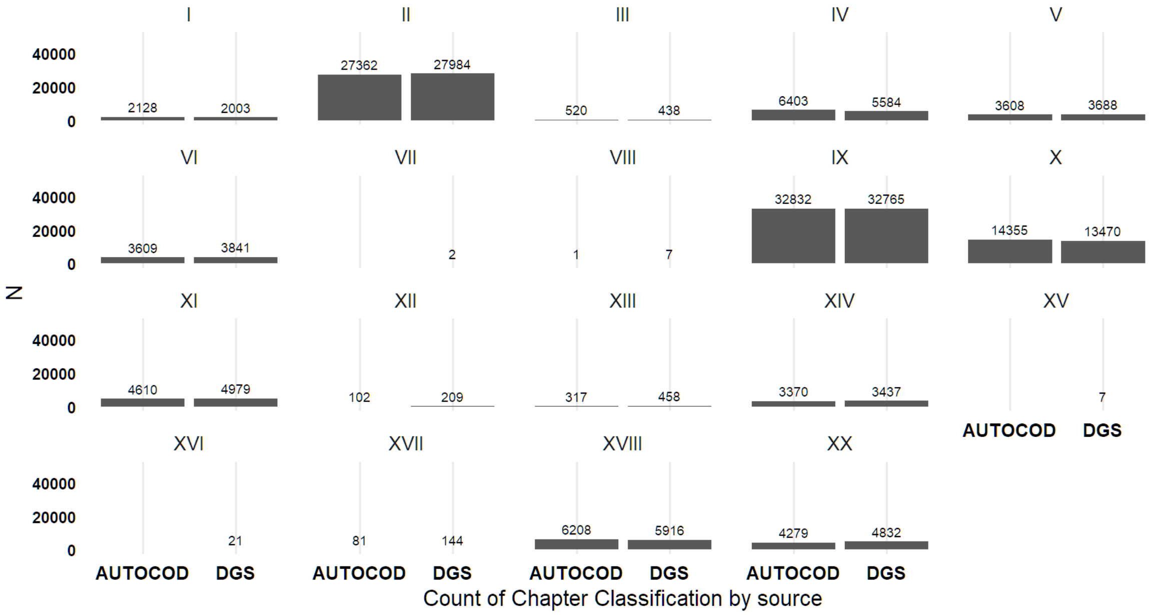

Supplementary Figure 1 - Count of death certificates classified either by human coders (DGS) or by AUTOCOD, for the year 2016

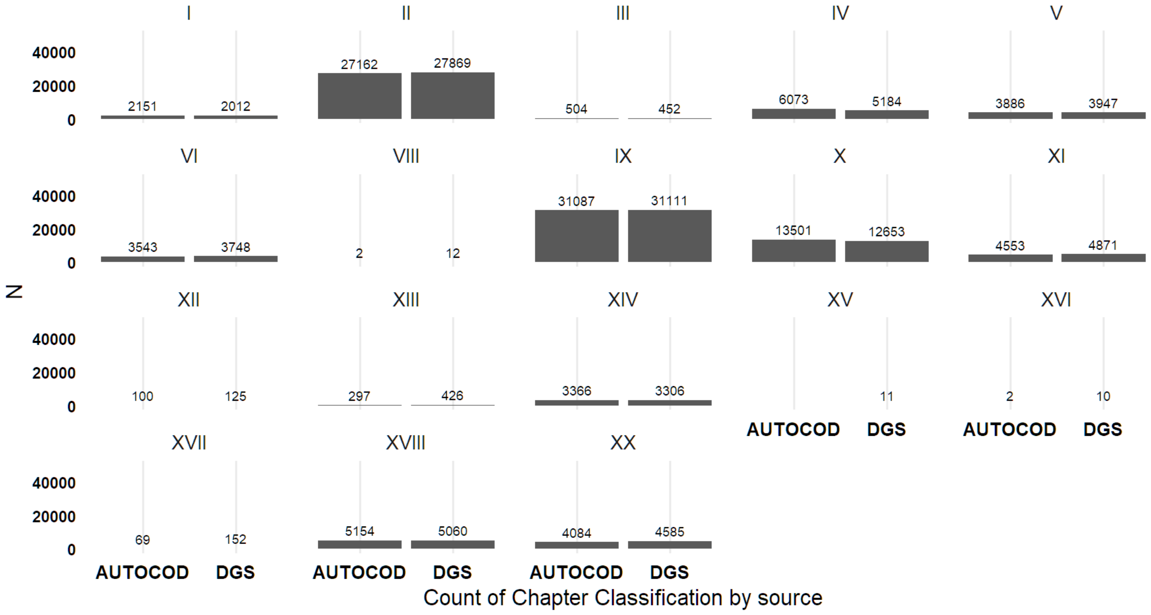

Supplementary Figure 2 - Count of death certificates classified either by human coders (DGS) or by AUTOCOD, for the year 2017

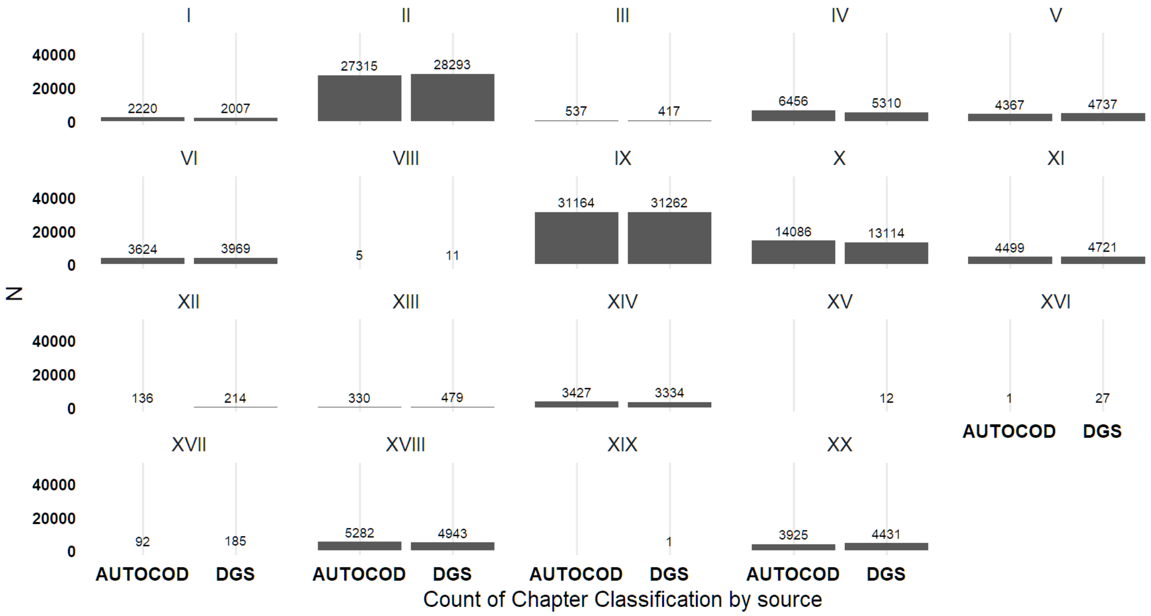

Supplementary Figure 3 - Count of death certificates classified either by human coders (DGS) or by AUTOCOD, for the year 2018

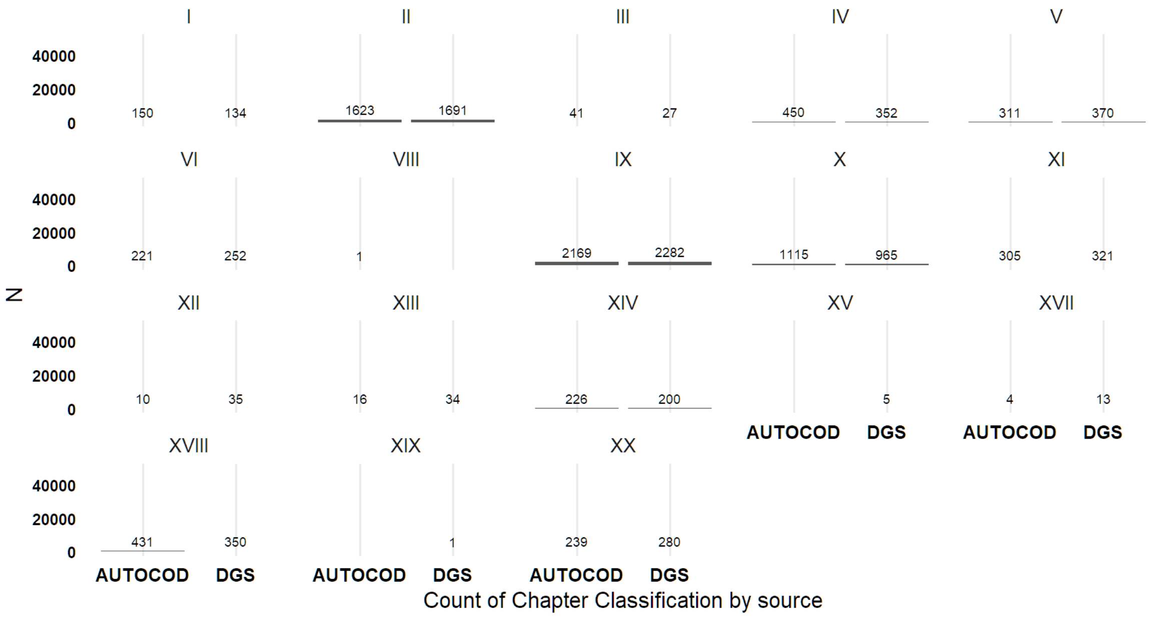

Supplementary Figure 4 - Count of death certificates classified either by human coders (DGS) or by AUTOCOD, for the year 2019

Supplementary Table 9 - Confusion Matrix for all the period analysed

Human Coders (gold-standard)

AUTOCOD	I	II	III	IV	V	VI	VII	VIII	IX	X	XI	XII	XIII	XIV	XV	XVI	XVII	XVIII	XIX	XX
I	4,119	271	48	192	116	115	0	2	406	235	414	189	78	229	0	6	17	70	0	142
II	188	81,476	49	118	105	101	0	2	424	219	378	11	33	112	2	2	18	40	0	184
III	31	234	757	47	29	33	0	2	171	44	102	6	15	53	2	7	8	4	0	57
IV	233	436	58	13,279	325	291	0	6	2,413	595	412	54	131	656	5	8	41	103	0	336
V	88	113	15	213	9,880	332	0	1	535	617	58	12	21	120	0	0	3	49	0	115
VI	88	198	24	132	110	9,408	1	2	387	265	82	6	26	52	0	4	26	33	0	153
VII	0	0	0	0	0	0	0	0	0	0	0	0	0	0	0	0	0	0	0	0
VIII	0	0	0	1	0	1	0	6	0	0	0	0	0	0	0	0	0	0	0	1
IX	254	838	138	1,183	788	469	0	4	88,883	1,565	741	58	157	674	19	3	97	564	0	817
X	490	908	100	521	854	618	0	2	1,751	35,962	312	26	242	335	4	9	45	215	1	662
XI	384	521	58	132	52	46	0	0	445	100	11,884	6	19	100	1	3	16	19	0	181
XII	35	15	1	24	10	3	0	0	31	2	18	173	5	10	0	0	0	1	0	20
XIII	35	43	13	20	12	24	0	1	69	27	26	2	583	16	1	2	4	6	0	76
XIV	99	399	31	345	244	164	0	0	718	158	248	23	23	7,823	0	1	15	15	0	83
XV	0	0	0	0	0	0	0	0	0	0	0	0	0	0	0	0	0	0	0	0
XVI	0	0	0	0	0	0	0	0	0	0	0	0	0	0	0	3	0	0	0	0
XVII	3	4	1	2	2	9	0	0	16	4	7	1	0	6	0	0	184	1	0	6
XVIII	61	169	13	158	135	89	1	2	667	271	85	10	14	46	0	1	9	15,064	0	280
XIX	0	0	0	0	0	0	0	0	0	0	0	0	0	0	0	0	0	0	0	0
XX	48	212	28	63	80	107	0	0	504	138	125	6	50	45	1	9	10	85	1	11,015

Caption: I - Certain infectious and parasitic diseases; II - Neoplasms; III - Diseases of the blood and blood-forming organs and certain disorders involving the immune system; IV - Endocrine, nutritional and metabolic diseases; V - Mental and behavioural disorders; VI - Diseases of the nervous system; VII - Diseases of the eye and adnexa; VIII - Diseases of the ear and mastoid process; IX - Diseases of the circulatory system; X - Diseases of the respiratory system; XI - Diseases of the digestive system; XII - Diseases of the skin and subcutaneous tissue; XIII - Diseases of the musculoskeletal system and connective tissue; XIV - Diseases of the genitourinary system; XV - Pregnancy, childbirth and the puerperium; XVI - Certain conditions originating in the perinatal period; XVII - Congenital malformations, deformations and chromosomal abnormalities; XVIII - Symptoms, signs and abnormal clinical and laboratory findings, not elsewhere specified; XIX - Injury, poisoning and certain other consequences of external causes; XX - External causes of morbidity and mortality

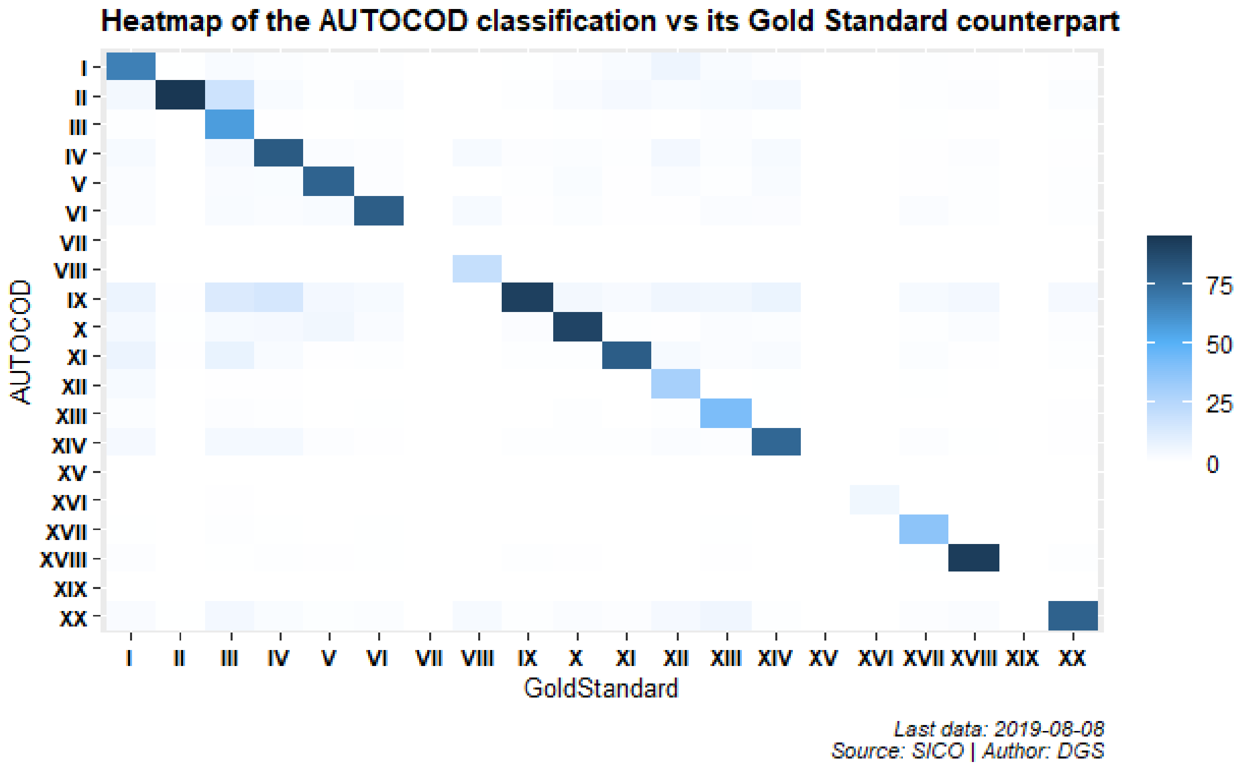

Supplementary Figure 5 - Heatmap of the AUTOCOD classification vs its Gold Standard counterpart. Values (%) on the diagonal represent correct classifications

Caption: I - Certain infectious and parasitic diseases; II - Neoplasms; III - Diseases of the blood and blood-forming organs and certain disorders involving the immune system; IV - Endocrine, nutritional and metabolic diseases; V - Mental and behavioural disorders; VI - Diseases of the nervous system; VII - Diseases of the eye and adnexa; VIII - Diseases of the ear and mastoid process; IX - Diseases of the circulatory system; X - Diseases of the respiratory system; XI - Diseases of the digestive system; XII - Diseases of the skin and subcutaneous tissue; XIII - Diseases of the musculoskeletal system and connective tissue; XIV - Diseases of the genitourinary system; XV - Pregnancy, childbirth and the puerperium; XVI - Certain conditions originating in the perinatal period; XVII - Congenital malformations, deformations and chromosomal abnormalities; XVIII - Symptoms, signs and abnormal clinical and laboratory findings, not elsewhere specified; XIX - Injury, poisoning and certain other consequences of external causes; XX - External causes of morbidity and mortality

Supplementary Table 10 - Overall performance metrics of AUTOCOD during all periods

**Accuracy**	0.88
**Kappa**	0.85
**AccuracyLower**	0.88
**AccuracyUpper**	0.88
**AccuracyNull**	0.30
**AccuracyPValue**	0.00

Supplementary Table 11 - Performance Metrics of AUTOCOD, by chapter, for all the periods analysed

Chapter	Sensitivity	Specificity	Pos Pred Value	Neg Pred Value	F1	Prevalence	Detection Rate	Detection Prevalence	Balanced Accuracy
**I**	0.67	0.99	0.62	0.99	0.64	0.02	0.01	0.02	0.83
**II**	0.95	0.99	0.98	0.98	0.96	0.26	0.25	0.25	0.97
**III**	0.57	1.00	0.47	1.00	0.52	0.00	0.00	0.01	0.78
**IV**	0.81	0.98	0.69	0.99	0.74	0.05	0.04	0.06	0.89
**V**	0.78	0.99	0.81	0.99	0.79	0.04	0.03	0.04	0.88
**VI**	0.80	1.00	0.86	0.99	0.83	0.04	0.03	0.03	0.90
**VII**	0.00	1.00	NaN	1.00	NA	0.00	0.00	0.00	0.50
**VIII**	0.20	1.00	0.67	1.00	0.31	0.00	0.00	0.00	0.60
**IX**	0.91	0.96	0.91	0.96	0.91	0.30	0.27	0.30	0.94
**X**	0.90	0.98	0.84	0.99	0.86	0.12	0.11	0.13	0.94
**XI**	0.80	0.99	0.85	0.99	0.82	0.05	0.04	0.04	0.90
**XII**	0.30	1.00	0.50	1.00	0.37	0.00	0.00	0.00	0.65
**XIII**	0.42	1.00	0.61	1.00	0.50	0.00	0.00	0.00	0.71
**XIV**	0.76	0.99	0.75	0.99	0.76	0.03	0.02	0.03	0.88
**XV**	0.00	1.00	NaN	1.00	NA	0.00	0.00	0.00	0.50
**XVI**	0.05	1.00	1.00	1.00	0.10	0.00	0.00	0.00	0.53
**XVII**	0.37	1.00	0.75	1.00	0.50	0.00	0.00	0.00	0.69
**XVIII**	0.93	0.99	0.88	1.00	0.90	0.05	0.05	0.05	0.96
**XIX**	0.00	1.00	NaN	1.00	NA	0.00	0.00	0.00	0.50
**XX**	0.78	1.00	0.88	0.99	0.83	0.04	0.03	0.04	0.89

Caption: I - Certain infectious and parasitic diseases; II - Neoplasms; III - Diseases of the blood and blood-forming organs and certain disorders involving the immune system; IV - Endocrine, nutritional and metabolic diseases; V - Mental and behavioural disorders; VI - Diseases of the nervous system; VII - Diseases of the eye and adnexa; VIII - Diseases of the ear and mastoid process; IX - Diseases of the circulatory system; X - Diseases of the respiratory system; XI - Diseases of the digestive system; XII - Diseases of the skin and subcutaneous tissue; XIII - Diseases of the musculoskeletal system and connective tissue; XIV - Diseases of the genitourinary system; XV - Pregnancy, childbirth and the puerperium; XVI - Certain conditions originating in the perinatal period; XVII - Congenital malformations, deformations and chromosomal abnormalities; XVIII - Symptoms, signs and abnormal clinical and laboratory findings, not elsewhere specified; XIX - Injury, poisoning and certain other consequences of external causes; XX - External causes of morbidity and mortality

Periods of excess mortality

Supplementary Table 12 - Distribution of death certificates in the dataset, according to source of classification, for the periods of excess mortality

Source	N	%
**AUTOCOD**	93 417	50.00
**DGS**	93 417	50.00
**Total**	**186 834**	**100.00**

Supplementary Table 13 - Distribution of death certificates in the dataset, according to ICD-10 chapter (descendent order), for the periods of excess mortality

Chapter	Chapter Description	N	%
**IX**	Diseases of the circulatory system	28 399	30.40
**II**	Neoplasms	21 136	22.63
**X**	Diseases of the respiratory system	13 466	14.41
**XVIII**	Symptoms, signs and abnormal clinical and laboratory findings, not elsewhere specified	5 197	5.56
**IV**	Endocrine, nutritional and metabolic diseases	4 793	5.13
**XI**	Diseases of the digestive system	3 893	4.17
**V**	Mental and behavioural disorders	3 756	4.02
**XX**	External causes of morbidity and mortality	3 631	3.89
**VI**	Diseases of the nervous system	3 456	3.70
**XIV**	Diseases of the genitourinary system	2 851	3.05
**I**	Certain infectious and parasitic diseases	1 696	1.82
**III**	Diseases of the blood and blood-forming organs and certain disorders involving the immune system	418	0.45
**XIII**	Diseases of the musculoskeletal system and connective tissue	406	0.43
**XII**	Diseases of the skin and subcutaneous tissue	153	0.16
**XVII**	Congenital malformations, deformations and chromosomal abnormalities	137	0.15
**XVI**	Certain conditions originating in the perinatal period	12	0.01
**VIII**	Diseases of the ear and mastoid process	8	0.01
**XV**	Pregnancy, childbirth and the puerperium	6	0.01
**XIX**	Injury, poisoning and certain other consequences of external causes	2	0.00
**VII**	Diseases of the eye and adnexa	1	0.00
**Total**		**93 417**	**100.00**

Supplementary Table 14 - Distribution of death certificates according to year of death, for the periods of excess mortality

Year of Death	N	%
**2016**	29 381	31.45
**2017**	30 263	32.40
**2018**	29 624	31.71
**2019**	4 149	4.44
**Total**	**93 417**	**100.00**

Supplementary Table 15 - Distribution of death certificates, according to year of death and ICD-10 chapter, for the periods of excess mortality

Year of Death	Chapter	Chapter Description	N	%
**2017**	IX	Diseases of the circulatory system	9 294	9.95
**2018**	IX	Diseases of the circulatory system	8 975	9.61
**2016**	IX	Diseases of the circulatory system	8 786	9.41
**2016**	II	Neoplasms	7 048	7.54
**2017**	II	Neoplasms	6 611	7.08
**2018**	II	Neoplasms	6 569	7.03
**2018**	X	Diseases of the respiratory system	4 574	4.90
**2017**	X	Diseases of the respiratory system	4 358	4.67
**2016**	X	Diseases of the respiratory system	3 960	4.24
**2016**	XVIII	Symptoms, signs and abnormal clinical and laboratory findings, not elsewhere specified	1 730	1.85
**2017**	XVIII	Symptoms, signs and abnormal clinical and laboratory findings, not elsewhere specified	1 725	1.85
**2018**	IV	Endocrine, nutritional and metabolic diseases	1 573	1.68
**2017**	IV	Endocrine, nutritional and metabolic diseases	1 562	1.67
**2018**	XVIII	Symptoms, signs and abnormal clinical and laboratory findings, not elsewhere specified	1 540	1.65
**2016**	IV	Endocrine, nutritional and metabolic diseases	1 479	1.58
**2019**	IX	Diseases of the circulatory system	1 344	1.44
**2016**	XI	Diseases of the digestive system	1 310	1.40
**2018**	V	Mental and behavioural disorders	1 244	1.33
**2017**	XI	Diseases of the digestive system	1 241	1.33
**2017**	V	Mental and behavioural disorders	1 221	1.31
**2017**	XX	External causes of morbidity and mortality	1 192	1.28
**2016**	XX	External causes of morbidity and mortality	1 175	1.26
**2017**	VI	Diseases of the nervous system	1 171	1.25
**2018**	XI	Diseases of the digestive system	1 161	1.24
**2018**	XX	External causes of morbidity and mortality	1 119	1.20
**2018**	VI	Diseases of the nervous system	1 073	1.15
**2016**	V	Mental and behavioural disorders	1 071	1.15
**2016**	VI	Diseases of the nervous system	1 065	1.14
**2017**	XIV	Diseases of the genitourinary system	938	1.00
**2018**	XIV	Diseases of the genitourinary system	925	0.99
**2019**	II	Neoplasms	908	0.97
**2016**	XIV	Diseases of the genitourinary system	873	0.93
**2017**	I	Certain infectious and parasitic diseases	574	0.61
**2019**	X	Diseases of the respiratory system	574	0.61
**2016**	I	Certain infectious and parasitic diseases	528	0.57
**2018**	I	Certain infectious and parasitic diseases	520	0.56
**2019**	V	Mental and behavioural disorders	220	0.24
**2019**	XVIII	Symptoms, signs and abnormal clinical and laboratory findings, not elsewhere specified	202	0.22
**2019**	XI	Diseases of the digestive system	181	0.19
**2019**	IV	Endocrine, nutritional and metabolic diseases	179	0.19
**2017**	III	Diseases of the blood and blood-forming organs and certain disorders involving the immune system	150	0.16
**2019**	VI	Diseases of the nervous system	147	0.16

Supplementary Table 16 - Distribution of death certificates, according to year of death and ICD-10 chapter, for the periods of excess mortality (cont.)

Year of Death	Chapter	Chapter Description	N	%
**2019**	XX	External causes of morbidity and mortality	145	0.16
**2017**	XIII	Diseases of the musculoskeletal system and connective tissue	139	0.15
**2016**	III	Diseases of the blood and blood-forming organs and certain disorders involving the immune system	130	0.14
**2018**	XIII	Diseases of the musculoskeletal system and connective tissue	128	0.14
**2018**	III	Diseases of the blood and blood-forming organs and certain disorders involving the immune system	126	0.13
**2016**	XIII	Diseases of the musculoskeletal system and connective tissue	120	0.13
**2019**	XIV	Diseases of the genitourinary system	115	0.12
**2019**	I	Certain infectious and parasitic diseases	74	0.08
**2016**	XII	Diseases of the skin and subcutaneous tissue	52	0.06
**2017**	XVII	Congenital malformations, deformations and chromosomal abnormalities	49	0.05
**2018**	XII	Diseases of the skin and subcutaneous tissue	48	0.05
**2016**	XVII	Congenital malformations, deformations and chromosomal abnormalities	45	0.05
**2018**	XVII	Congenital malformations, deformations and chromosomal abnormalities	37	0.04
**2017**	XII	Diseases of the skin and subcutaneous tissue	34	0.04
**2019**	XII	Diseases of the skin and subcutaneous tissue	19	0.02
**2019**	XIII	Diseases of the musculoskeletal system and connective tissue	19	0.02
**2019**	III	Diseases of the blood and blood-forming organs and certain disorders involving the immune system	12	0.01
**2016**	XVI	Certain conditions originating in the perinatal period	6	0.01
**2018**	XVI	Certain conditions originating in the perinatal period	6	0.01
**2019**	XVII	Congenital malformations, deformations and chromosomal abnormalities	6	0.01
**2018**	VIII	Diseases of the ear and mastoid process	5	0.01
**2019**	XV	Pregnancy, childbirth and the puerperium	3	0.00
**2017**	VIII	Diseases of the ear and mastoid process	2	0.00
**2017**	XV	Pregnancy, childbirth and the puerperium	2	0.00
**2016**	VII	Diseases of the eye and adnexa	1	0.00
**2016**	VIII	Diseases of the ear and mastoid process	1	0.00
**2016**	XV	Pregnancy, childbirth and the puerperium	1	0.00
**2018**	XIX	Injury, poisoning and certain other consequences of external causes	1	0.00
**2019**	XIX	Injury, poisoning and certain other consequences of external causes	1	0.00
**Total**			**93 417**	**100.00**

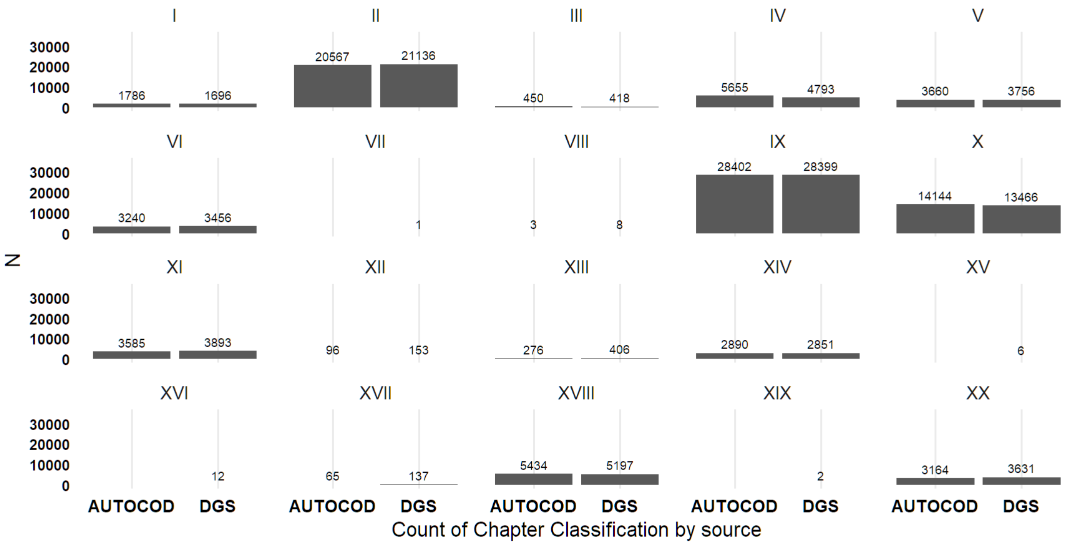

Supplementary Figure 6 – Count of death certificates classified either by human coders (DGS) or by AUTOCOD, for periods of excess mortality

Supplementary Table 17 - Confusion Matrix for the excess mortality periods

Human Coders (GOLD-STANDARD)

AUTOCOD	I	II	III	IV	V	VI	VII	VIII	IX	X	XI	XII	XIII	XIV	XV	XVI	XVII	XVIII	XIX	XX
I	1135	76	8	51	27	23	0	0	93	87	113	41	18	57	0	2	3	23	0	29
II	38	19990	20	36	19	32	0	1	143	64	114	2	9	32	0	0	7	9	0	51
III	4	61	231	12	8	8	0	0	43	12	25	1	3	13	0	4	2	2	0	21
IV	70	120	23	3880	94	82	0	2	698	231	99	21	33	171	0	0	10	31	0	90
V	39	23	6	69	2924	107	0	1	157	226	14	2	8	34	0	0	2	13	0	35
VI	26	47	4	35	31	2774	1	0	112	99	21	3	5	19	0	0	7	13	0	43
VII	0	0	0	0	0	0	0	0	0	0	0	0	0	0	0	0	0	0	0	0
VIII	0	0	0	0	0	0	0	2	0	0	0	0	0	0	0	0	0	0	0	1
IX	81	222	45	361	245	139	0	0	25882	517	205	16	43	207	4	0	32	183	0	220
X	147	271	31	157	253	176	0	1	546	11993	96	4	85	94	2	4	12	72	1	199
XI	80	120	23	27	15	9	0	0	125	29	3058	0	4	29	0	1	8	9	0	48
XII	8	3	0	7	5	0	0	0	4	1	2	54	2	4	0	0	0	0	0	6
XIII	6	6	2	3	5	8	0	1	24	9	10	1	169	5	0	0	0	3	0	24
XIV	22	106	10	96	62	39	0	0	216	57	88	3	7	2158	0	0	3	6	0	17
XV	0	0	0	0	0	0	0	0	0	0	0	0	0	0	0	0	0	0	0	0
XVI	0	0	0	0	0	0	0	0	0	0	0	0	0	0	0	0	0	0	0	0
XVII	1	1	1	0	2	3	0	0	3	2	2	0	0	3	0	0	46	0	0	1
XVIII	26	45	7	45	44	29	0	0	216	98	18	3	2	14	0	1	2	4806	0	78
XIX	0	0	0	0	0	0	0	0	0	0	0	0	0	0	0	0	0	0	0	0
XX	13	45	7	14	22	27	0	0	137	41	28	2	18	11	0	0	3	27	1	2768

Caption: I - Certain infectious and parasitic diseases; II - Neoplasms; III - Diseases of the blood and blood-forming organs and certain disorders involving the immune system; IV - Endocrine, nutritional and metabolic diseases; V - Mental and behavioural disorders; VI - Diseases of the nervous system; VII - Diseases of the eye and adnexa; VIII - Diseases of the ear and mastoid process; IX - Diseases of the circulatory system; X - Diseases of the respiratory system; XI - Diseases of the digestive system; XII - Diseases of the skin and subcutaneous tissue; XIII - Diseases of the musculoskeletal system and connective tissue; XIV - Diseases of the genitourinary system; XV - Pregnancy, childbirth and the puerperium; XVI - Certain conditions originating in the perinatal period; XVII - Congenital malformations, deformations and chromosomal abnormalities; XVIII - Symptoms, signs and abnormal clinical and laboratory findings, not elsewhere specified; XIX - Injury, poisoning and certain other consequences of external causes; XX - External causes of morbidity and mortality

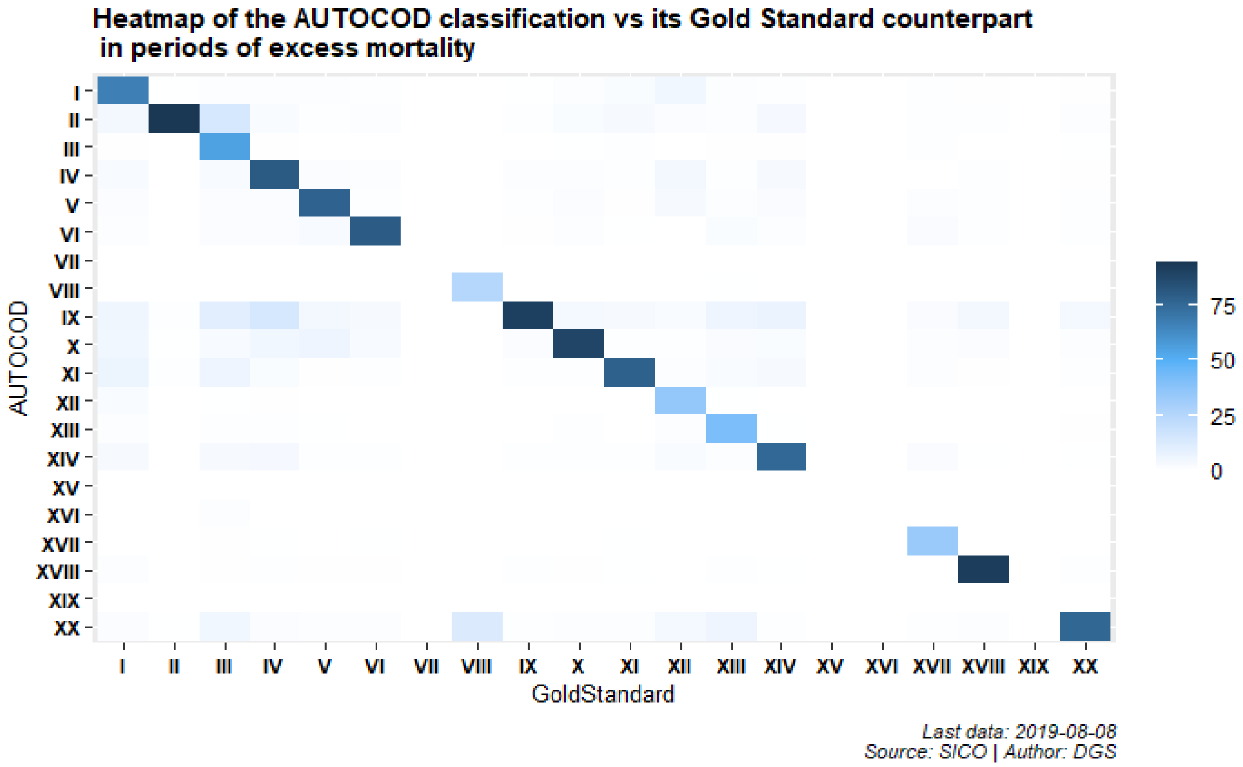

Supplementary Figure 7 - Heatmap of the AUTOCOD classification vs its Gold Standard counterpart in periods of excess mortality. Values on the diagonal represent correct classifications.

Caption: I - Certain infectious and parasitic diseases; II - Neoplasms; III - Diseases of the blood and blood-forming organs and certain disorders involving the immune system; IV - Endocrine, nutritional and metabolic diseases; V - Mental and behavioural disorders; VI - Diseases of the nervous system; VII - Diseases of the eye and adnexa; VIII - Diseases of the ear and mastoid process; IX - Diseases of the circulatory system; X - Diseases of the respiratory system; XI - Diseases of the digestive system; XII - Diseases of the skin and subcutaneous tissue; XIII - Diseases of the musculoskeletal system and connective tissue; XIV - Diseases of the genitourinary system; XV - Pregnancy, childbirth and the puerperium; XVI - Certain conditions originating in the perinatal period; XVII - Congenital malformations, deformations and chromosomal abnormalities; XVIII - Symptoms, signs and abnormal clinical and laboratory findings, not elsewhere specified; XIX - Injury, poisoning and certain other consequences of external causes; XX - External causes of morbidity and mortality

Supplementary Table 18 - Overall performance metrics of AUTOCOD during excess mortality periods

**Accuracy**	0.88
**Kappa**	0.85
**AccuracyLower**	0.87
**AccuracyUpper**	0.88
**AccuracyNull**	0.30
**AccuracyPValue**	0.00

Supplementary Table 19 - Performance Metrics of AUTOCOD, by class, for the excess mortality periods

Chapter	Sensitivity	Specificity	Pos Pred Value	Neg Pred Value	F1	Prevalence	Detection Rate	Detection Prevalence	Balanced Accuracy
**I**	0.67	0.99	0.64	0.99	0.65	0.02	0.01	0.02	0.83
**II**	0.95	0.99	0.97	0.98	0.96	0.23	0.21	0.22	0.97
**III**	0.55	1.00	0.51	1.00	0.53	0.00	0.00	0.01	0.78
**IV**	0.81	0.98	0.69	0.99	0.74	0.05	0.04	0.06	0.90
**V**	0.78	0.99	0.80	0.99	0.79	0.04	0.03	0.04	0.89
**VI**	0.80	1.00	0.86	0.99	0.83	0.04	0.03	0.04	0.90
**VII**	0.00	1.00	NaN	1.00	NA	0.00	0.00	0.00	0.50
**VIII**	0.25	1.00	0.67	1.00	0.36	0.00	0.00	0.00	0.63
**IX**	0.91	0.96	0.91	0.96	0.91	0.30	0.28	0.30	0.94
**X**	0.89	0.97	0.85	0.98	0.87	0.14	0.13	0.15	0.93
**XI**	0.79	0.99	0.85	0.99	0.82	0.04	0.03	0.04	0.89
**XII**	0.35	1	0.56	1	0.43	0	0	0	0.68
**XIII**	0.42	1	0.61	1	0.5	0	0	0	0.71
**XIV**	0.76	0.99	0.75	0.99	0.75	0.03	0.02	0.03	0.87
**XV**	0	1	NaN	1	NA	0	0	0	0.5
**XVI**	0	1	NaN	1	NA	0	0	0	0.5
**XVII**	0.34	1	0.71	1	0.46	0	0	0	0.67
**XVIII**	0.93	0.99	0.88	1	0.9	0.06	0.05	0.06	0.96
**XIX**	0	1	NaN	1	NA	0	0	0	0.5
**XX**	0.76	1	0.88	0.99	0.82	0.04	0.03	0.03	0.88

Caption: I - Certain infectious and parasitic diseases; II - Neoplasms; III - Diseases of the blood and blood-forming organs and certain disorders involving the immune system; IV - Endocrine, nutritional and metabolic diseases; V - Mental and behavioural disorders; VI - Diseases of the nervous system; VII - Diseases of the eye and adnexa; VIII - Diseases of the ear and mastoid process; IX - Diseases of the circulatory system; X - Diseases of the respiratory system; XI - Diseases of the digestive system; XII - Diseases of the skin and subcutaneous tissue; XIII - Diseases of the musculoskeletal system and connective tissue; XIV - Diseases of the genitourinary system; XV - Pregnancy, childbirth and the puerperium; XVI - Certain conditions originating in the perinatal period; XVII - Congenital malformations, deformations and chromosomal abnormalities; XVIII - Symptoms, signs and abnormal clinical and laboratory findings, not elsewhere specified; XIX - Injury, poisoning and certain other consequences of external causes; XX - External causes of morbidity and mortality

Periods without excess mortality

Supplementary Table 20 - Distribution of death certificates in the dataset, according to source of classification, for the periods without excess mortality

Source	N	%
**AUTOCOD**	236 681	50.00
**DGS**	236 681	50.00
**Total**	**473 362**	**100.00**

Supplementary Table 21 - Distribution of death certificates in the dataset, according to ICD-10 chapter (descendent order), for the periods without excess mortality

Chapter	Chapter Description	N	%
**IX**	Diseases of the circulatory system	69 021	29.16
**II**	Neoplasms	64 701	27.34
**X**	Diseases of the respiratory system	26 736	11.30
**IV**	Endocrine, nutritional and metabolic diseases	11 637	4.92
**XVIII**	Symptoms, signs and abnormal clinical and laboratory findings, not elsewhere specified	11 072	4.68
**XI**	Diseases of the digestive system	10 999	4.65
**XX**	External causes of morbidity and mortality	10 497	4.44
**V**	Mental and behavioural disorders	8 986	3.80
**VI**	Diseases of the nervous system	8 354	3.53
**XIV**	Diseases of the genitourinary system	7 426	3.14
**I**	Certain infectious and parasitic diseases	4 460	1.88
**XIII**	Diseases of the musculoskeletal system and connective tissue	991	0.42
**III**	Diseases of the blood and blood-forming organs and certain disorders involving the immune system	916	0.39
**XII**	Diseases of the skin and subcutaneous tissue	430	0.18
**XVII**	Congenital malformations, deformations and chromosomal abnormalities	357	0.15
**XVI**	Certain conditions originating in the perinatal period	46	0.02
**XV**	Pregnancy, childbirth and the puerperium	29	0.01
**VIII**	Diseases of the ear and mastoid process	22	0.01
**VII**	Diseases of the eye and adnexa	1	0.00
	**Total**	**236 681**	**100.00**

Supplementary Table 22 - Distribution of death certificates according to year of death, for the periods without excess mortality

Year of Death	N	%
**2016**	80 404	33.97
**2017**	75 271	31.80
**2018**	77 843	32.89
**2019**	3 163	1.34
**Total**	**236 681**	**100.00**

Supplementary Table 23 - Distribution of death certificates, according to year of death and ICD-10 chapter, for the periods without excess mortality

Year of Death	Chapter	Chapter Description	N	%
**2016**	IX	Diseases of the circulatory system	23 979	10.13
**2018**	IX	Diseases of the circulatory system	22 287	9.42
**2017**	IX	Diseases of the circulatory system	21 817	9.22
**2018**	II	Neoplasms	21 724	9.18
**2017**	II	Neoplasms	21 258	8.98
**2016**	II	Neoplasms	20 936	8.85
**2016**	X	Diseases of the respiratory system	9 510	4.02
**2018**	X	Diseases of the respiratory system	8 540	3.61
**2017**	X	Diseases of the respiratory system	8 295	3.50
**2016**	XVIII	Symptoms, signs and abnormal clinical and laboratory findings, not elsewhere specified	4 186	1.77
**2016**	IV	Endocrine, nutritional and metabolic diseases	4 105	1.73
**2018**	IV	Endocrine, nutritional and metabolic diseases	3 737	1.58
**2016**	XI	Diseases of the digestive system	3 669	1.55
**2016**	XX	External causes of morbidity and mortality	3 657	1.55
**2017**	XI	Diseases of the digestive system	3 630	1.53
**2017**	IV	Endocrine, nutritional and metabolic diseases	3 622	1.53
**2018**	XI	Diseases of the digestive system	3 560	1.50
**2018**	V	Mental and behavioural disorders	3 493	1.48
**2018**	XVIII	Symptoms, signs and abnormal clinical and laboratory findings, not elsewhere specified	3 403	1.44
**2017**	XX	External causes of morbidity and mortality	3 393	1.43
**2017**	XVIII	Symptoms, signs and abnormal clinical and laboratory findings, not elsewhere specified	3 335	1.41
**2018**	XX	External causes of morbidity and mortality	3 312	1.40
**2018**	VI	Diseases of the nervous system	2 896	1.22
**2016**	VI	Diseases of the nervous system	2 776	1.17
**2017**	V	Mental and behavioural disorders	2 726	1.15
**2016**	V	Mental and behavioural disorders	2 617	1.11
**2017**	VI	Diseases of the nervous system	2 577	1.09
**2016**	XIV	Diseases of the genitourinary system	2 564	1.08
**2018**	XIV	Diseases of the genitourinary system	2 409	1.02
**2017**	XIV	Diseases of the genitourinary system	2 368	1.00
**2018**	I	Certain infectious and parasitic diseases	1 487	0.63
**2016**	I	Certain infectious and parasitic diseases	1 475	0.62
**2017**	I	Certain infectious and parasitic diseases	1 438	0.61
**2019**	IX	Diseases of the circulatory system	938	0.40
**2019**	II	Neoplasms	783	0.33
**2019**	X	Diseases of the respiratory system	391	0.17
**2018**	XIII	Diseases of the musculoskeletal system and connective tissue	351	0.15
**2016**	XIII	Diseases of the musculoskeletal system and connective tissue	338	0.14
**2016**	III	Diseases of the blood and blood-forming organs and certain disorders involving the immune system	308	0.13
**2017**	III	Diseases of the blood and blood-forming organs and certain disorders involving the immune system	302	0.13
**2018**	III	Diseases of the blood and blood-forming organs and certain disorders involving the immune system	291	0.12
**2017**	XIII	Diseases of the musculoskeletal system and connective tissue	287	0.12

Supplementary Table 24 - Distribution of death certificates, according to year of death and ICD-10 chapter, for the periods without excess mortality (cont.)

Year of Death	Chapter	Chapter Description	N	%
**2019**	IV	Endocrine, nutritional and metabolic diseases	173	0.07
**2018**	XII	Diseases of the skin and subcutaneous tissue	166	0.07
**2016**	XII	Diseases of the skin and subcutaneous tissue	157	0.07
**2019**	V	Mental and behavioural disorders	150	0.06
**2018**	XVII	Congenital malformations, deformations and chromosomal abnormalities	148	0.06
**2019**	XVIII	Symptoms, signs and abnormal clinical and laboratory findings, not elsewhere specified	148	0.06
**2019**	XI	Diseases of the digestive system	140	0.06
**2019**	XX	External causes of morbidity and mortality	135	0.06
**2019**	VI	Diseases of the nervous system	105	0.04
**2017**	XVII	Congenital malformations, deformations and chromosomal abnormalities	103	0.04
**2016**	XVII	Congenital malformations, deformations and chromosomal abnormalities	99	0.04
**2017**	XII	Diseases of the skin and subcutaneous tissue	91	0.04
**2019**	XIV	Diseases of the genitourinary system	85	0.04
**2019**	I	Certain infectious and parasitic diseases	60	0.03
**2018**	XVI	Certain conditions originating in the perinatal period	21	0.01
**2019**	XII	Diseases of the skin and subcutaneous tissue	16	0.01
**2016**	XVI	Certain conditions originating in the perinatal period	15	0.01
**2019**	III	Diseases of the blood and blood-forming organs and certain disorders involving the immune system	15	0.01
**2019**	XIII	Diseases of the musculoskeletal system and connective tissue	15	0.01
**2018**	XV	Pregnancy, childbirth and the puerperium	12	0.01
**2017**	VIII	Diseases of the ear and mastoid process	10	0.00
**2017**	XVI	Certain conditions originating in the perinatal period	10	0.00
**2017**	XV	Pregnancy, childbirth and the puerperium	9	0.00
**2019**	XVII	Congenital malformations, deformations and chromosomal abnormalities	7	0.00
**2016**	VIII	Diseases of the ear and mastoid process	6	0.00
**2016**	XV	Pregnancy, childbirth and the puerperium	6	0.00
**2018**	VIII	Diseases of the ear and mastoid process	6	0.00
**2019**	XV	Pregnancy, childbirth and the puerperium	2	0.00
**2016**	VII	Diseases of the eye and adnexa	1	0.00
**Total**			**236 681**	**100.00**

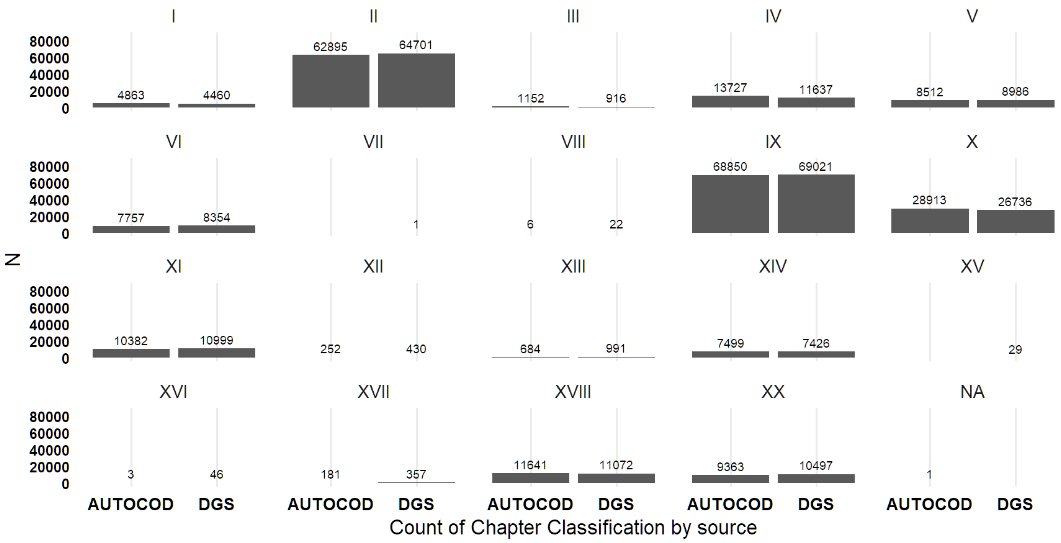

Supplementary Figure 8 - Count of death certificates classified either by human coders (DGS) or by AUTOCOD, for periods without excess mortality

Supplementary Table 25 - Confusion Matrix for the periods without excess mortality

Human Coders (GOLD-STANDARD) (columns) × **AUTOCOD** (rows)

AUTOCOD	I	II	III	IV	V	VI	VII	VIII	IX	X	XI	XII	XIII	XIV	XV	XVI	XVII	XVIII	XX
I	2984	195	40	141	89	92	0	2	313	148	301	148	60	172	0	4	14	47	113
II	150	61486	29	82	86	69	0	1	281	155	264	9	24	80	2	2	11	31	133
III	27	173	526	35	21	25	0	2	128	32	77	5	12	40	2	3	6	2	36
IV	163	316	35	9399	231	209	0	4	1715	364	313	33	98	485	5	8	31	72	246
V	49	90	9	144	6956	225	0	0	378	391	44	10	13	86	0	0	1	36	80
VI	62	151	20	97	79	6634	0	2	275	166	61	3	21	33	0	4	19	20	110
VII	0	0	0	0	0	0	0	0	0	0	0	0	0	0	0	0	0	0	0
VIII	0	0	0	1	0	1	0	4	0	0	0	0	0	0	0	0	0	0	0
IX	173	616	93	822	543	330	0	4	63001	1048	536	42	114	467	15	3	65	381	597
X	343	637	69	364	601	442	0	1	1205	23969	216	22	157	241	2	5	33	143	463
XI	304	401	35	105	37	37	0	0	320	71	8826	6	15	71	1	2	8	10	133
XII	27	12	1	17	5	3	0	0	27	1	16	119	3	6	0	0	0	1	14
XIII	29	37	11	17	7	16	0	0	45	18	16	1	414	11	1	2	4	3	52
XIV	77	293	21	249	182	125	0	0	502	101	160	20	16	5665	0	1	12	9	66
XV	0	0	0	0	0	0	0	0	0	0	0	0	0	0	0	0	0	0	0
XVI	0	0	0	0	0	0	0	0	0	0	0	0	0	0	0	3	0	0	0
XVII	2	3	0	2	0	6	0	0	13	2	5	1	0	3	0	0	138	1	5
XVIII	35	124	6	113	91	60	1	2	451	173	67	7	12	32	0	0	7	10258	202
XX	35	167	21	49	58	80	0	0	367	97	97	4	32	34	1	9	7	58	8247

Caption: I - Certain infectious and parasitic diseases; II - Neoplasms; III - Diseases of the blood and blood-forming organs and certain disorders involving the immune system; IV - Endocrine, nutritional and metabolic diseases; V - Mental and behavioural disorders; VI - Diseases of the nervous system; VII - Diseases of the eye and adnexa; VIII - Diseases of the ear and mastoid process; IX - Diseases of the circulatory system; X - Diseases of the respiratory system; XI - Diseases of the digestive system; XII - Diseases of the skin and subcutaneous tissue; XIII - Diseases of the musculoskeletal system and connective tissue; XIV - Diseases of the genitourinary system; XV - Pregnancy, childbirth and the puerperium; XVI - Certain conditions originating in the perinatal period; XVII - Congenital malformations, deformations and chromosomal abnormalities; XVIII - Symptoms, signs and abnormal clinical and laboratory findings, not elsewhere specified; XIX - Injury, poisoning and certain other consequences of external causes; XX - External causes of morbidity and mortality

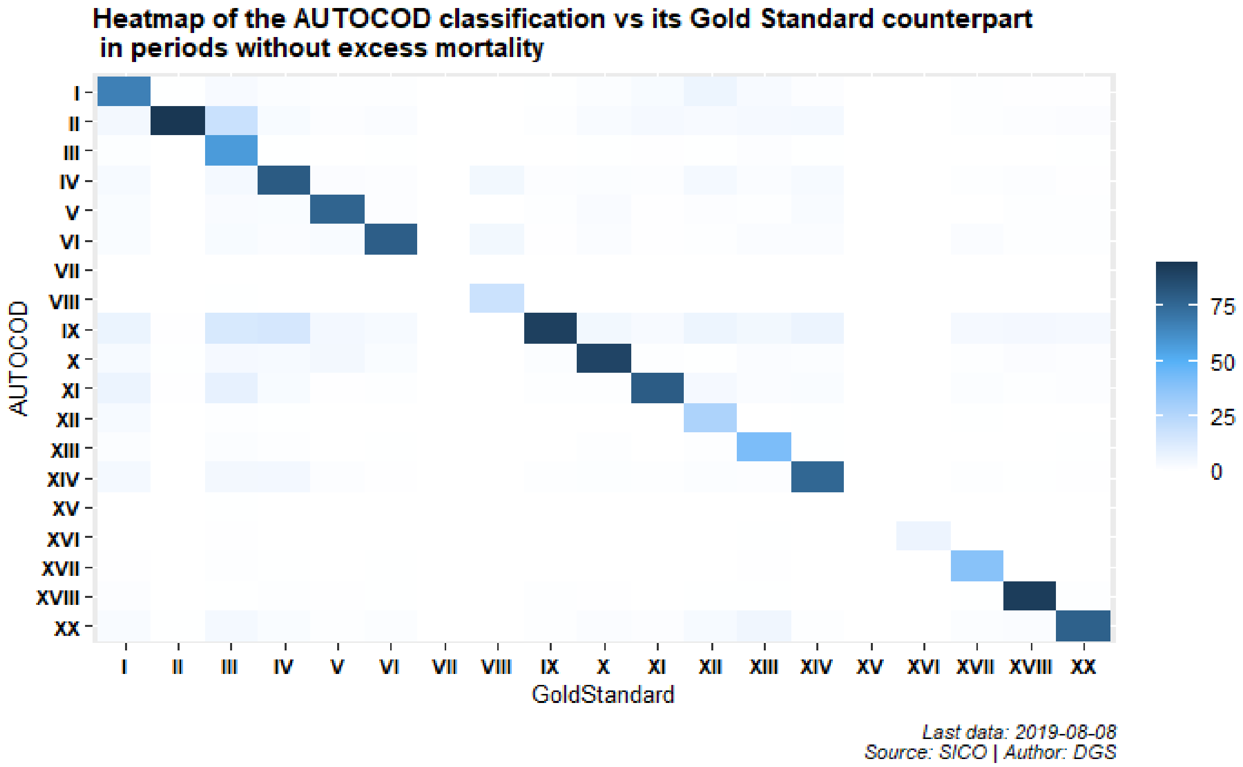

Supplementary Figure 9 - Heatmap of the AUTOCOD classification vs its Gold Standard counterpart in periods without excess mortality. Values on the diagonal represent correct classifications.

Caption: I - Certain infectious and parasitic diseases; II - Neoplasms; III - Diseases of the blood and blood-forming organs and certain disorders involving the immune system; IV - Endocrine, nutritional and metabolic diseases; V - Mental and behavioural disorders; VI - Diseases of the nervous system; VII - Diseases of the eye and adnexa; VIII - Diseases of the ear and mastoid process; IX - Diseases of the circulatory system; X - Diseases of the respiratory system; XI - Diseases of the digestive system; XII - Diseases of the skin and subcutaneous tissue; XIII - Diseases of the musculoskeletal system and connective tissue; XIV - Diseases of the genitourinary system; XV - Pregnancy, childbirth and the puerperium; XVI - Certain conditions originating in the perinatal period; XVII - Congenital malformations, deformations and chromosomal abnormalities; XVIII - Symptoms, signs and abnormal clinical and laboratory findings, not elsewhere specified; XIX - Injury, poisoning and certain other consequences of external causes; XX - External causes of morbidity and mortality

Supplementary Table 26 - Overall performance metrics of AUTOCOD during periods without excess mortality

**Accuracy**	0.88
**Kappa**	0.86
**AccuracyLower**	0.88
**AccuracyUpper**	0.88
**AccuracyNull**	0.29
**AccuracyPValue**	0.00

Supplementary Table 27 - Performance Metrics of AUTOCOD, by class, for the periods without excess mortality

Chapter	Sensitivity	Specificity	Pos Pred Value	Neg Pred Value	F1	Prevalence	Detection Rate	Detection Prevalence	Balanced Accuracy
**I**	0.67	0.99	0.61	0.99	0.64	0.02	0.01	0.02	0.83
**II**	0.95	0.99	0.98	0.98	0.96	0.27	0.26	0.27	0.97
**III**	0.57	1.00	0.46	1.00	0.51	0.00	0.00	0.01	0.79
**IV**	0.81	0.98	0.69	0.99	0.74	0.05	0.04	0.06	0.89
**V**	0.77	0.99	0.82	0.99	0.80	0.04	0.03	0.04	0.88
**VI**	0.79	1.00	0.86	0.99	0.82	0.04	0.03	0.03	0.90
**VII**	0.00	1.00	NaN	1.00	NA	0.00	0.00	0.00	0.50
**VIII**	0.18	1.00	0.67	1.00	0.29	0.00	0.00	0.00	0.59
**IX**	0.91	0.97	0.92	0.96	0.91	0.29	0.27	0.29	0.94
**X**	0.90	0.98	0.83	0.99	0.86	0.11	0.10	0.12	0.94
**XI**	0.80	0.99	0.85	0.99	0.83	0.05	0.04	0.04	0.90
**XII**	0.28	1.00	0.47	1.00	0.35	0.00	0.00	0.00	0.64
**XIII**	0.42	1.00	0.61	1.00	0.49	0.00	0.00	0.00	0.71
**XIV**	0.76	0.99	0.76	0.99	0.76	0.03	0.02	0.03	0.88
**XV**	0.00	1.00	NaN	1.00	NA	0.00	0.00	0.00	0.50
**XVI**	0.07	1.00	1.00	1.00	0.12	0.00	0.00	0.00	0.53
**XVII**	0.39	1.00	0.76	1.00	0.51	0.00	0.00	0.00	0.69
**XVIII**	0.93	0.99	0.88	1.00	0.90	0.05	0.04	0.05	0.96
**XX**	0.79	1.00	0.88	0.99	0.83	0.04	0.04	0.04	0.89

Caption: I - Certain infectious and parasitic diseases; II - Neoplasms; III - Diseases of the blood and blood-forming organs and certain disorders involving the immune system; IV - Endocrine, nutritional and metabolic diseases; V - Mental and behavioural disorders; VI - Diseases of the nervous system; VII - Diseases of the eye and adnexa; VIII - Diseases of the ear and mastoid process; IX - Diseases of the circulatory system; X - Diseases of the respiratory system; XI - Diseases of the digestive system; XII - Diseases of the skin and subcutaneous tissue; XIII - Diseases of the musculoskeletal system and connective tissue; XIV - Diseases of the genitourinary system; XV - Pregnancy, childbirth and the puerperium; XVI - Certain conditions originating in the perinatal period; XVII - Congenital malformations, deformations and chromosomal abnormalities; XVIII - Symptoms, signs and abnormal clinical and laboratory findings, not elsewhere specified; XIX - Injury, poisoning and certain other consequences of external causes; XX - External causes of morbidity and mortality

Periods with severe excess mortality (>4 SDs)

Supplementary Table 28 - Confusion Matrix for the periods with severe excess mortality (>4 SDs)

Human Coders (GOLD-STANDARD)

AUTOCOD	I	II	III	IV	V	VI	VII	VIII	IX	X	XI	XII	XIII	XIV	XV	XVI	XVII	XVIII	XIX	XX
I	366	18	1	17	7	10	0	0	35	27	36	8	6	14	0	0	0	7	0	14
II	10	5746	8	14	6	12	0	1	56	17	37	1	3	11	0	0	1	3	0	15
III	1	10	80	3	3	1	0	0	18	4	7	1	0	9	0	3	1	1	0	8
IV	24	35	5	1287	36	38	0	0	218	87	38	4	11	52	0	0	2	11	0	32
V	16	8	1	21	981	37	0	0	52	67	4	0	3	14	0	0	0	6	0	11
VI	7	12	0	11	16	867	0	0	38	39	6	1	2	5	0	0	1	3	0	16
VII	0	0	0	0	0	0	0	0	0	0	0	0	0	0	0	0	0	0	0	0
VIII	0	0	0	0	0	0	0	2	0	0	0	0	0	0	0	0	0	0	0	1
IX	19	77	14	132	92	52	0	0	8416	167	78	3	14	67	2	0	10	71	0	82
X	52	76	13	56	76	49	0	1	188	4237	32	2	24	27	0	0	4	27	1	69
XI	28	44	9	10	5	6	0	0	45	14	914	0	4	9	0	1	4	2	0	13
XII	3	1	0	0	3	0	0	0	1	1	0	15	1	2	0	0	0	0	0	1
XIII	1	5	1	1	1	2	0	1	9	5	5	1	54	2	0	0	0	1	0	7
XIV	9	24	1	24	16	7	0	0	81	21	27	0	4	704	0	0	2	3	0	4
XV	0	0	0	0	0	0	0	0	0	0	0	0	0	0	0	0	0	0	0	0
XVI	0	0	0	0	0	0	0	0	0	0	0	0	0	0	0	0	0	0	0	0
XVII	0	0	0	0	1	1	0	0	1	1	1	0	0	0	0	0	12	0	0	0
XVIII	6	20	3	16	16	9	0	0	80	30	4	0	0	4	0	1	0	1657	0	33
XIX	0	0	0	0	0	0	0	0	0	0	0	0	0	0	0	0	0	0	0	0
XX	4	12	3	2	5	6	0	0	49	17	12	2	4	4	0	0	1	10	1	908

Caption: I - Certain infectious and parasitic diseases; II - Neoplasms; III - Diseases of the blood and blood-forming organs and certain disorders involving the immune system; IV - Endocrine, nutritional and metabolic diseases; V - Mental and behavioural disorders; VI - Diseases of the nervous system; VII - Diseases of the eye and adnexa; VIII - Diseases of the ear and mastoid process; IX - Diseases of the circulatory system; X - Diseases of the respiratory system; XI - Diseases of the digestive system; XII - Diseases of the skin and subcutaneous tissue; XIII - Diseases of the musculoskeletal system and connective tissue; XIV - Diseases of the genitourinary system; XV - Pregnancy, childbirth and the puerperium; XVI - Certain conditions originating in the perinatal period; XVII - Congenital malformations, deformations and chromosomal abnormalities; XVIII - Symptoms, signs and abnormal clinical and laboratory findings, not elsewhere specified; XIX - Injury, poisoning and certain other consequences of external causes; XX - External causes of morbidity and mortality

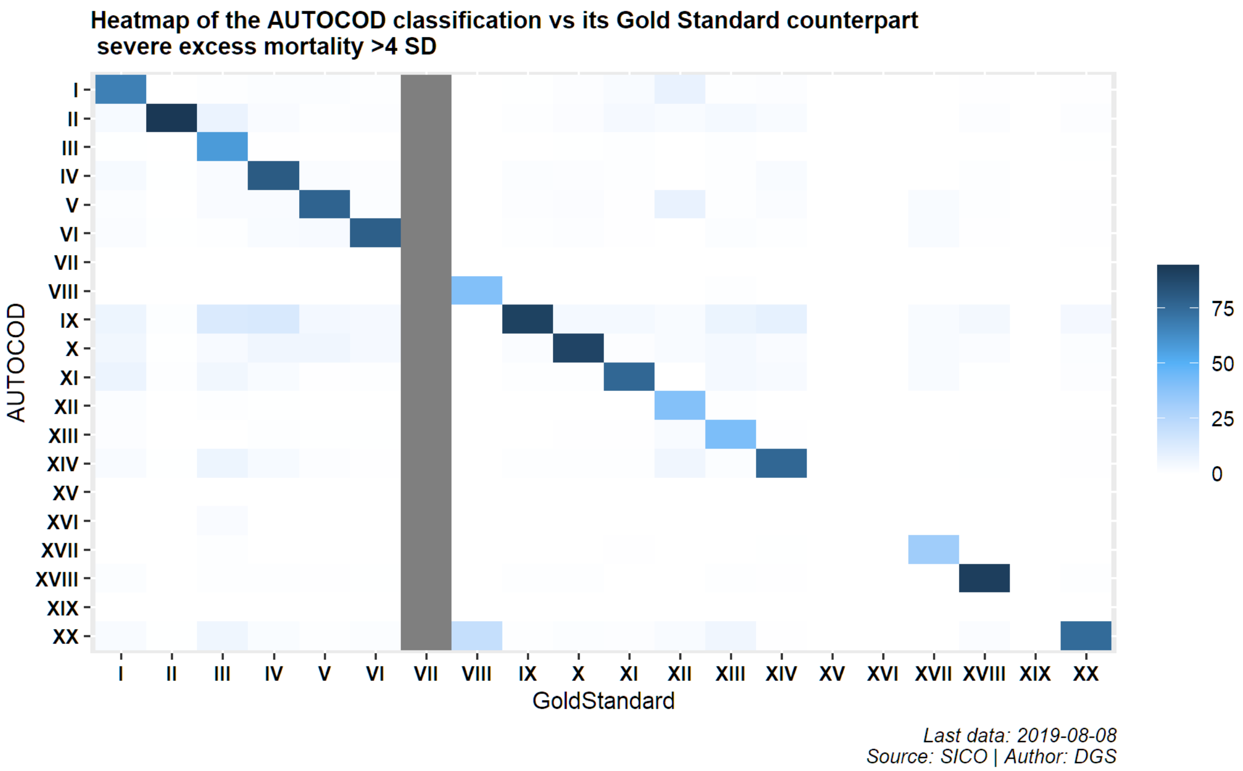

Supplementary Figure 10 - Heatmap of the AUTOCOD classification vs its Gold Standard counterpart in periods with severe excess mortality (> 4SDs). Values on the diagonal represent correct classifications.

Caption: I - Certain infectious and parasitic diseases; II - Neoplasms; III - Diseases of the blood and blood-forming organs and certain disorders involving the immune system; IV - Endocrine, nutritional and metabolic diseases; V - Mental and behavioural disorders; VI - Diseases of the nervous system; VII - Diseases of the eye and adnexa; VIII - Diseases of the ear and mastoid process; IX - Diseases of the circulatory system; X - Diseases of the respiratory system; XI - Diseases of the digestive system; XII - Diseases of the skin and subcutaneous tissue; XIII - Diseases of the musculoskeletal system and connective tissue; XIV - Diseases of the genitourinary system; XV - Pregnancy, childbirth and the puerperium; XVI - Certain conditions originating in the perinatal period; XVII - Congenital malformations, deformations and chromosomal abnormalities; XVIII - Symptoms, signs and abnormal clinical and laboratory findings, not elsewhere specified; XIX - Injury, poisoning and certain other consequences of external causes; XX - External causes of morbidity and mortality

Supplementary Table 29 - Overall performance metrics of AUTOCOD during periods with severe excess mortality (>4 SDs)

**Accuracy**	0.87
**Kappa**	0.84
**AccuracyLower**	0.87
**AccuracyUpper**	0.88
**AccuracyNull**	0.31
**AccuracyPValue**	0.00

Table 1 - Performance Metrics of AUTOCOD, by class, during periods with severe excess mortality (>4 SDs)

Chapter	Sensitivity	Specificity	Pos Pred Value	Neg Pred Value	F1	Prevalence	Detection Rate	Detection Prevalence	Balanced Accuracy
**I**	0.67	0.99	0.65	0.99	0.66	0.02	0.01	0.02	0.83
**II**	0.94	0.99	0.97	0.99	0.96	0.20	0.19	0.20	0.97
**III**	0.58	1.00	0.53	1.00	0.55	0.01	0.00	0.01	0.79
**IV**	0.81	0.98	0.69	0.99	0.74	0.05	0.04	0.06	0.89
**V**	0.78	0.99	0.80	0.99	0.79	0.04	0.03	0.04	0.88
**VI**	0.79	1.00	0.85	0.99	0.82	0.04	0.03	0.03	0.89
**VII**	NA	1.00	NA	NA	NA	0.00	0.00	0.00	NA
**VIII**	0.40	1.00	0.67	1.00	0.50	0.00	0.00	0.00	0.70
**IX**	0.91	0.96	0.91	0.96	0.91	0.31	0.28	0.31	0.93
**X**	0.90	0.97	0.86	0.98	0.88	0.16	0.14	0.16	0.93
**XI**	0.76	0.99	0.83	0.99	0.79	0.04	0.03	0.04	0.88
**XII**	0.40	1.00	0.54	1.00	0.46	0.00	0.00	0.00	0.70
**XIII**	0.42	1.00	0.56	1.00	0.48	0.00	0.00	0.00	0.71
**XIV**	0.76	0.99	0.76	0.99	0.76	0.03	0.02	0.03	0.88
**XV**	0.00	1.00	NA	1.00	NA	0.00	0.00	0.00	0.50
**XVI**	0.00	1.00	NA	1.00	NA	0.00	0.00	0.00	0.50
**XVII**	0.32	1.00	0.71	1.00	0.44	0.00	0.00	0.00	0.66
**XVIII**	0.92	0.99	0.88	1.00	0.90	0.06	0.06	0.06	0.96
**XIX**	0.00	1.00	NA	1.00	NA	0.00	0.00	0.00	0.50
**XX**	0.75	1.00	0.87	0.99	0.81	0.04	0.03	0.04	0.87

Caption: I - Certain infectious and parasitic diseases; II - Neoplasms; III - Diseases of the blood and blood-forming organs and certain disorders involving the immune system; IV - Endocrine, nutritional and metabolic diseases; V - Mental and behavioural disorders; VI - Diseases of the nervous system; VII - Diseases of the eye and adnexa; VIII - Diseases of the ear and mastoid process; IX - Diseases of the circulatory system; X - Diseases of the respiratory system; XI - Diseases of the digestive system; XII - Diseases of the skin and subcutaneous tissue; XIII - Diseases of the musculoskeletal system and connective tissue; XIV - Diseases of the genitourinary system; XV - Pregnancy, childbirth and the puerperium; XVI - Certain conditions originating in the perinatal period; XVII - Congenital malformations, deformations and chromosomal abnormalities; XVIII - Symptoms, signs and abnormal clinical and laboratory findings, not elsewhere specified; XIX - Injury, poisoning and certain other consequences of external causes; XX - External causes of morbidity and mortality

Periods with extreme excess mortality (>6 SDs)

Supplementary Table 30 - Confusion Matrix for the periods with extreme excess mortality (>6 SDs)

AUTOCOD \ Human Coders (GOLD-STANDARD)	I	II	III	IV	V	VI	VII	VIII	IX	X	XI	XII	XIII	XIV	XV	XVI	XVII	XVIII	XIX	XX
I	72	1	0	5	1	3	0	0	10	7	7	0	2	4	0	0	0	2	0	3
II	0	1112	4	1	2	2	0	0	11	7	14	1	1	2	0	0	1	0	0	4
III	0	2	9	1	0	0	0	0	4	1	1	1	0	5	0	0	0	0	0	1
IV	5	6	1	258	9	10	0	0	36	22	4	0	1	10	0	0	1	4	0	7
V	4	2	0	3	216	8	0	0	13	9	0	0	0	1	0	0	0	1	0	4
VI	1	1	0	2	2	200	0	0	5	13	0	0	1	0	0	0	0	1	0	2
VII	0	0	0	0	0	0	0	0	0	0	0	0	0	0	0	0	0	0	0	0
VIII	0	0	0	0	0	0	0	0	0	0	0	0	0	0	0	0	0	0	0	0
IX	5	13	3	23	18	15	0	0	1750	30	10	1	4	11	1	0	5	27	0	21
X	12	14	4	9	20	11	0	0	40	905	5	0	6	6	0	0	1	6	0	11
XI	6	7	1	1	2	0	0	0	8	0	165	0	0	2	0	0	0	0	0	3
XII	0	0	0	0	0	0	0	0	0	0	0	1	0	2	0	0	0	0	0	0
XIII	0	0	0	0	0	1	0	0	1	3	1	0	9	1	0	0	0	0	0	2
XIV	3	1	0	5	3	1	0	0	14	4	6	0	2	133	0	0	0	1	0	1
XV	0	0	0	0	0	0	0	0	0	0	0	0	0	0	0	0	0	0	0	0
XVI	0	0	0	0	0	0	0	0	0	0	0	0	0	0	0	0	0	0	0	0
XVII	0	0	0	0	0	0	0	0	0	0	1	0	0	0	0	0	2	0	0	0
XVIII	1	5	0	5	7	1	0	0	18	9	1	0	0	0	0	0	0	401	0	6
XIX	0	0	0	0	0	0	0	0	0	0	0	0	0	0	0	0	0	0	0	0
XX	2	2	0	0	1	2	0	0	8	4	2	1	2	2	0	0	0	5	0	208

Caption: I - Certain infectious and parasitic diseases; II - Neoplasms; III - Diseases of the blood and blood-forming organs and certain disorders involving the immune system; IV - Endocrine, nutritional and metabolic diseases; V - Mental and behavioural disorders; VI - Diseases of the nervous system; VII - Diseases of the eye and adnexa; VIII - Diseases of the ear and mastoid process; IX - Diseases of the circulatory system; X - Diseases of the respiratory system; XI - Diseases of the digestive system; XII - Diseases of the skin and subcutaneous tissue; XIII - Diseases of the musculoskeletal system and connective tissue; XIV - Diseases of the genitourinary system; XV - Pregnancy, childbirth and the puerperium; XVI - Certain conditions originating in the perinatal period; XVII - Congenital malformations, deformations and chromosomal abnormalities; XVIII - Symptoms, signs and abnormal clinical and laboratory findings, not elsewhere specified; XIX - Injury, poisoning and certain other consequences of external causes; XX - External causes of morbidity and mortality

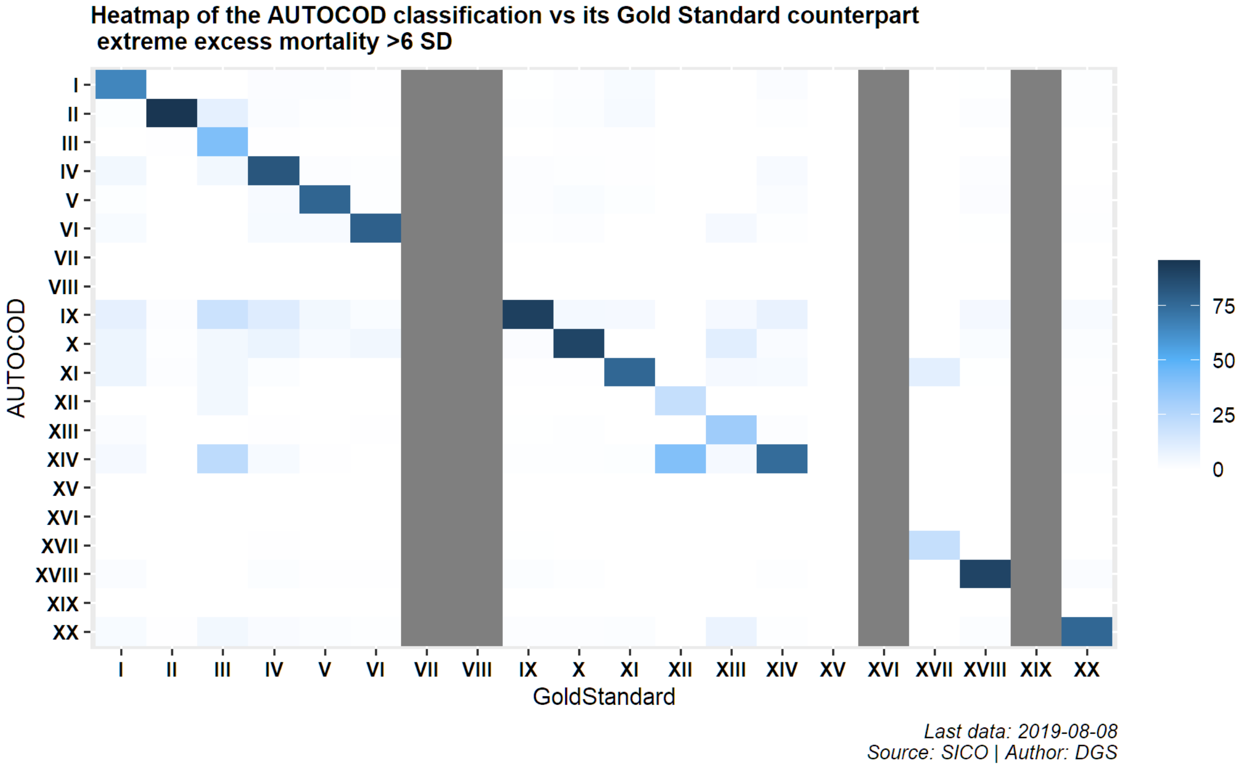

Supplementary Figure 11 - Heatmap of the AUTOCOD classification vs its Gold Standard counterpart in periods with extreme excess mortality (>6 SDs). Values on the diagonal represent correct classifications.

Caption: I - Certain infectious and parasitic diseases; II - Neoplasms; III - Diseases of the blood and blood-forming organs and certain disorders involving the immune system; IV - Endocrine, nutritional and metabolic diseases; V - Mental and behavioural disorders; VI - Diseases of the nervous system; VII - Diseases of the eye and adnexa; VIII - Diseases of the ear and mastoid process; IX - Diseases of the circulatory system; X - Diseases of the respiratory system; XI - Diseases of the digestive system; XII - Diseases of the skin and subcutaneous tissue; XIII - Diseases of the musculoskeletal system and connective tissue; XIV - Diseases of the genitourinary system; XV - Pregnancy, childbirth and the puerperium; XVI - Certain conditions originating in the perinatal period; XVII - Congenital malformations, deformations and chromosomal abnormalities; XVIII - Symptoms, signs and abnormal clinical and laboratory findings, not elsewhere specified; XIX - Injury, poisoning and certain other consequences of external causes; XX - External causes of morbidity and mortality

Supplementary Table 31 - Overall performance metrics of AUTOCOD during periods with extreme excess mortality (>6 SDs)

**Accuracy**	0.87
**Kappa**	0.85
**AccuracyLower**	0.86
**AccuracyUpper**	0.88
**AccuracyNull**	0.31
**AccuracyPValue**	0.00

Supplementary Table 32 - Performance Metrics of AUTOCOD, by class, during periods with extreme excess mortality (>6 SDs)

Chapter	Sensitivity	Specificity	Pos Pred Value	Neg Pred Value	F1	Prevalence	Detection Rate	Detection Prevalence	Balanced Accuracy
**I**	0.65	0.99	0.62	0.99	0.63	0.02	0.01	0.02	0.82
**II**	0.95	0.99	0.96	0.99	0.96	0.19	0.18	0.19	0.97
**III**	0.41	1.00	0.36	1.00	0.38	0.00	0.00	0.00	0.70
**IV**	0.82	0.98	0.69	0.99	0.75	0.05	0.04	0.06	0.90
**V**	0.77	0.99	0.83	0.99	0.80	0.05	0.04	0.04	0.88
**VI**	0.79	1.00	0.88	0.99	0.83	0.04	0.03	0.04	0.89
**VII**	NA	1.00	NA	NA	NA	0.00	0.00	0.00	NA
**VIII**	NA	1.00	NA	NA	NA	0.00	0.00	0.00	NA
**IX**	0.91	0.96	0.96	0.96	0.91	0.31	0.28	0.31	0.94
**X**	0.89	0.97	0.86	0.98	0.88	0.16	0.15	0.17	0.93
**XI**	0.76	1.00	0.85	0.99	0.80	0.04	0.03	0.03	0.88
**XII**	0.20	1.00	0.33	1.00	0.25	0.00	0.00	0.00	0.60
**XIII**	0.32	1.00	0.50	1.00	0.39	0.00	0.00	0.00	0.66
**XIV**	0.74	0.99	0.76	0.99	0.75	0.03	0.02	0.03	0.87
**XV**	0.00	1.00	NA	1.00	NA	0.00	0.00	0.00	0.50
**XVI**	NA	1.00	NA	NA	NA	0.00	0.00	0.00	NA
**XVII**	0.20	1.00	0.67	1.00	0.31	0.00	0.00	0.00	0.60
**XVIII**	0.90	0.99	0.88	0.99	0.89	0.07	0.06	0.07	0.94
**XIX**	NA	1.00	NA	NA	NA	0.00	0.00	0.00	NA
**XX**	0.76	1.00	0.87	0.99	0.81	0.04	0.03	0.04	0.88

Caption: I - Certain infectious and parasitic diseases; II - Neoplasms; III - Diseases of the blood and blood-forming organs and certain disorders involving the immune system; IV - Endocrine, nutritional and metabolic diseases; V - Mental and behavioural disorders; VI - Diseases of the nervous system; VII - Diseases of the eye and adnexa; VIII - Diseases of the ear and mastoid process; IX - Diseases of the circulatory system; X - Diseases of the respiratory system; XI - Diseases of the digestive system; XII - Diseases of the skin and subcutaneous tissue; XIII - Diseases of the musculoskeletal system and connective tissue; XIV - Diseases of the genitourinary system; XV - Pregnancy, childbirth and the puerperium; XVI - Certain conditions originating in the perinatal period; XVII - Congenital malformations, deformations and chromosomal abnormalities; XVIII - Symptoms, signs and abnormal clinical and laboratory findings, not elsewhere specified; XIX - Injury, poisoning and certain other consequences of external causes; XX - External causes of morbidity and mortality

Difference between excess mortality periods and periods without excess mortality

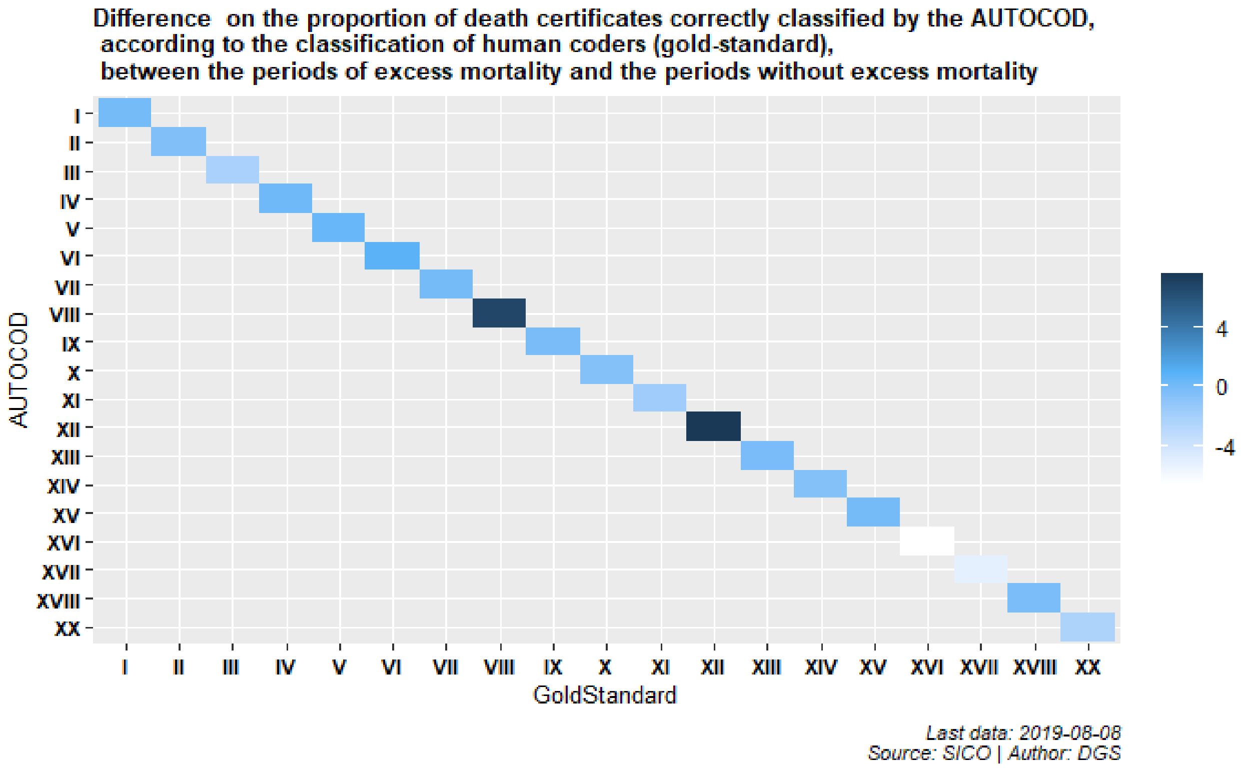

Supplementary Figure 12 - Difference on the proportion of death certificates correctly classified by the AUTOCOD, according to the classification of human coders (gold-standard), between the periods of excess mortality and the periods without excess mortality

Caption: I - Certain infectious and parasitic diseases; II - Neoplasms; III - Diseases of the blood and blood-forming organs and certain disorders involving the immune system; IV - Endocrine, nutritional and metabolic diseases; V - Mental and behavioural disorders; VI - Diseases of the nervous system; VII - Diseases of the eye and adnexa; VIII - Diseases of the ear and mastoid process; IX - Diseases of the circulatory system; X - Diseases of the respiratory system; XI - Diseases of the digestive system; XII - Diseases of the skin and subcutaneous tissue; XIII - Diseases of the musculoskeletal system and connective tissue; XIV - Diseases of the genitourinary system; XV - Pregnancy, childbirth and the puerperium; XVI - Certain conditions originating in the perinatal period; XVII - Congenital malformations, deformations and chromosomal abnormalities; XVIII - Symptoms, signs and abnormal clinical and laboratory findings, not elsewhere specified; XIX - Injury, poisoning and certain other consequences of external causes; XX - External causes of morbidity and mortality

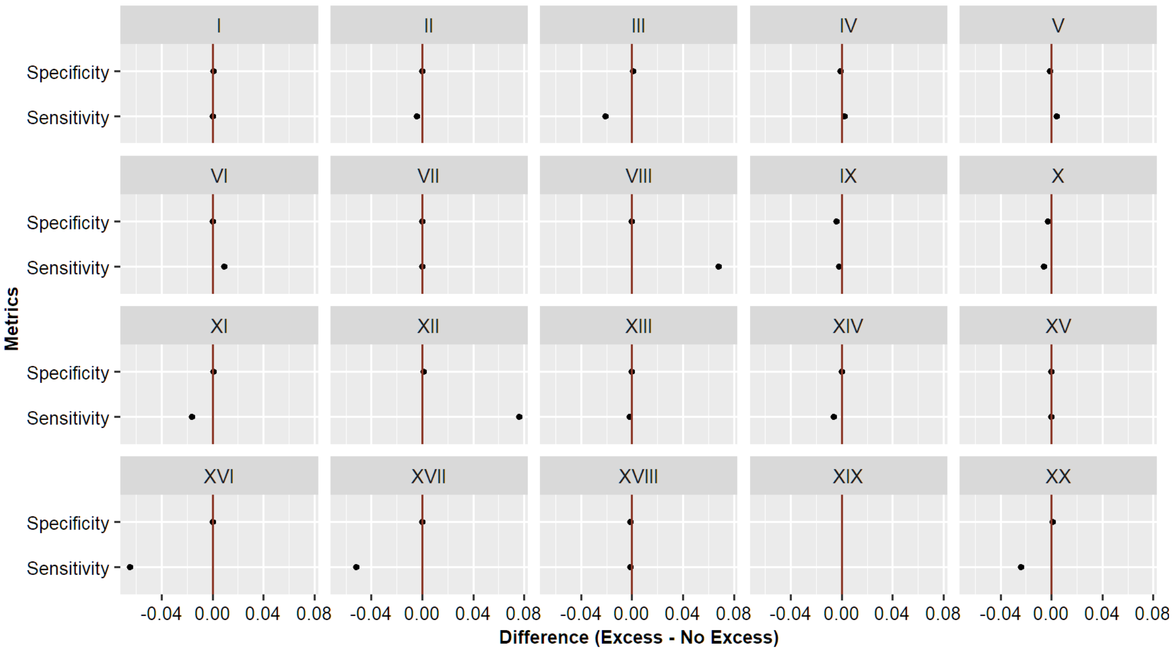

Supplementary Figure 13 - Difference on performance metrics of AUTOCOD, by chapter, between periods of excess mortality and periods without excess mortality

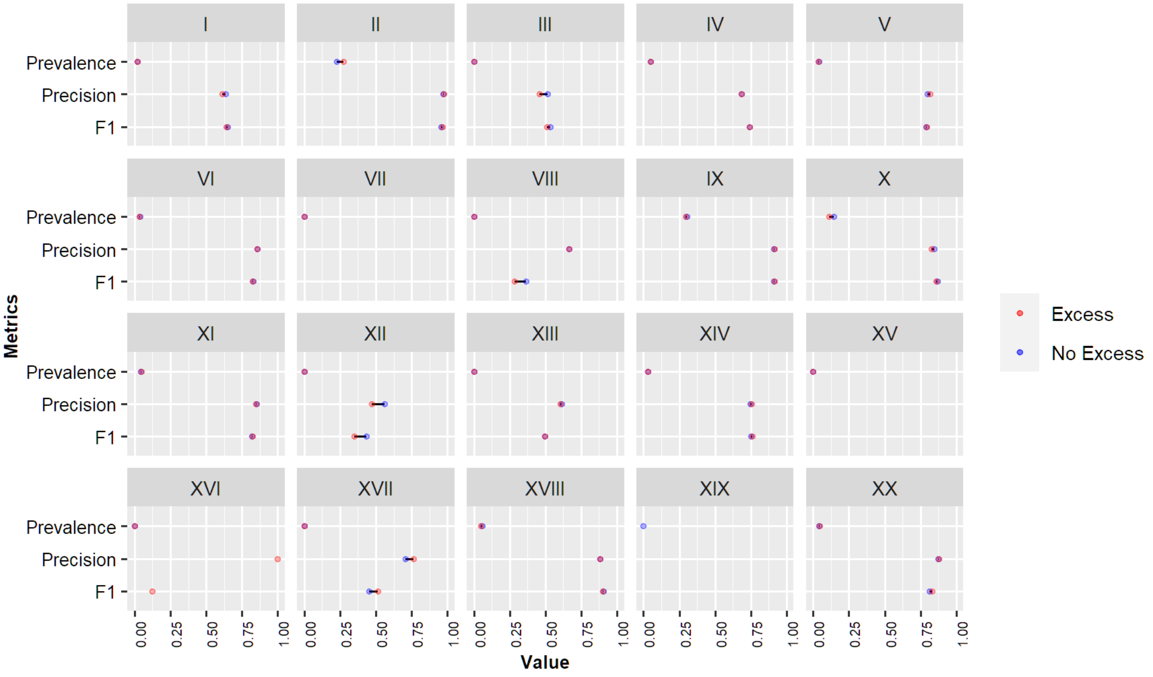

Supplementary Figure 14 - Comparison between other performance metrics of AUTOCOD during periods of excess mortality and periods without excess mortality

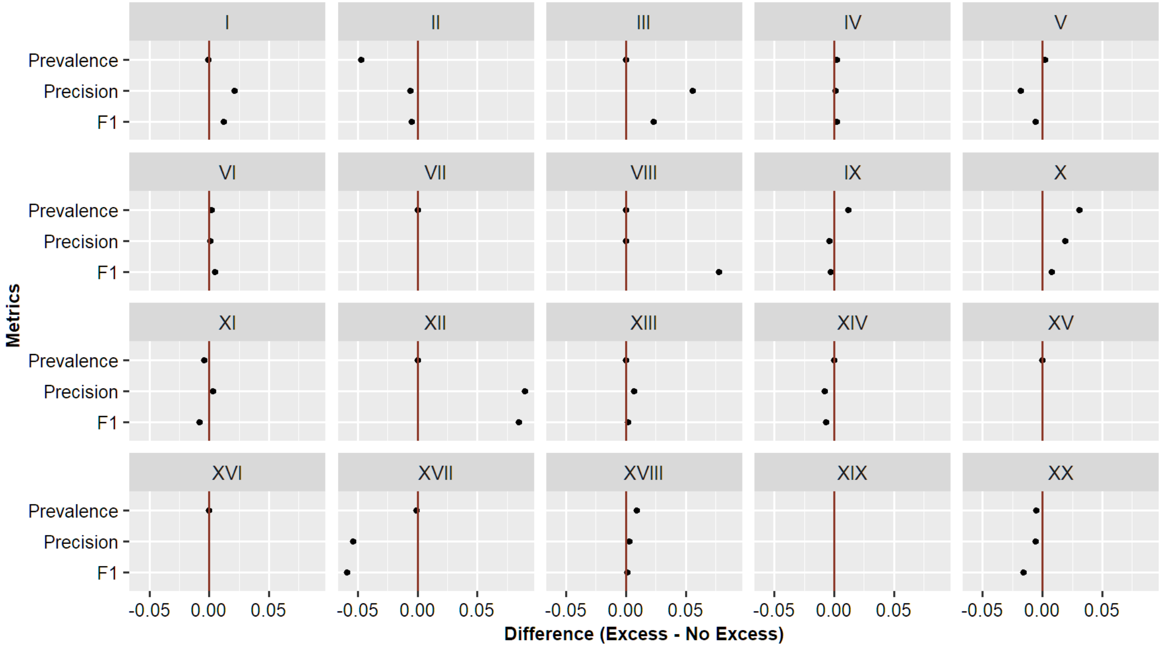

Supplementary Figure 15 - Difference on other performance metrics of AUTOCOD, by chapter, between periods of excess mortality and periods without excess mortality

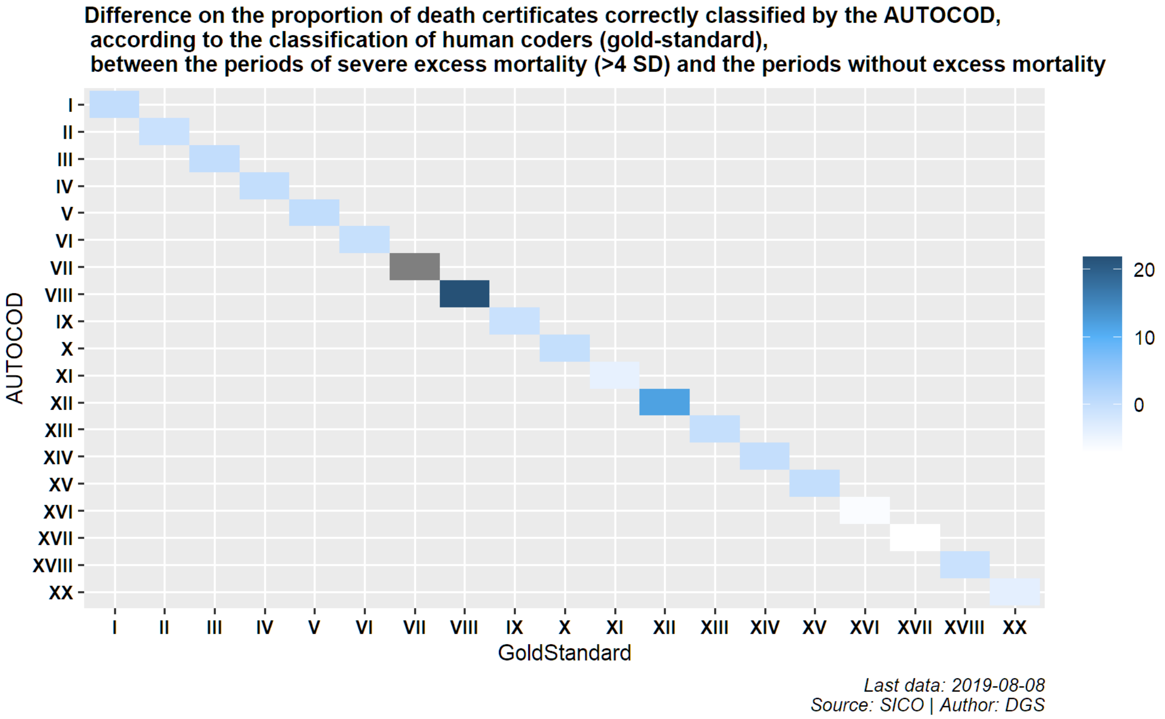

Supplementary Figure 16 -Difference on the proportion of death certificates correctly classified by the AUTOCOD, according to the classification of human coders (gold-standard), between the periods of severe excess mortality (>4 SDs) and the periods without excess mortality

Caption: I - Certain infectious and parasitic diseases; II - Neoplasms; III - Diseases of the blood and blood-forming organs and certain disorders involving the immune system; IV - Endocrine, nutritional and metabolic diseases; V - Mental and behavioural disorders; VI - Diseases of the nervous system; VII - Diseases of the eye and adnexa; VIII - Diseases of the ear and mastoid process; IX - Diseases of the circulatory system; X - Diseases of the respiratory system; XI - Diseases of the digestive system; XII - Diseases of the skin and subcutaneous tissue; XIII - Diseases of the musculoskeletal system and connective tissue; XIV - Diseases of the genitourinary system; XV - Pregnancy, childbirth and the puerperium; XVI - Certain conditions originating in the perinatal period; XVII - Congenital malformations, deformations and chromosomal abnormalities; XVIII - Symptoms, signs and abnormal clinical and laboratory findings, not elsewhere specified; XIX - Injury, poisoning and certain other consequences of external causes; XX - External causes of morbidity and mortality

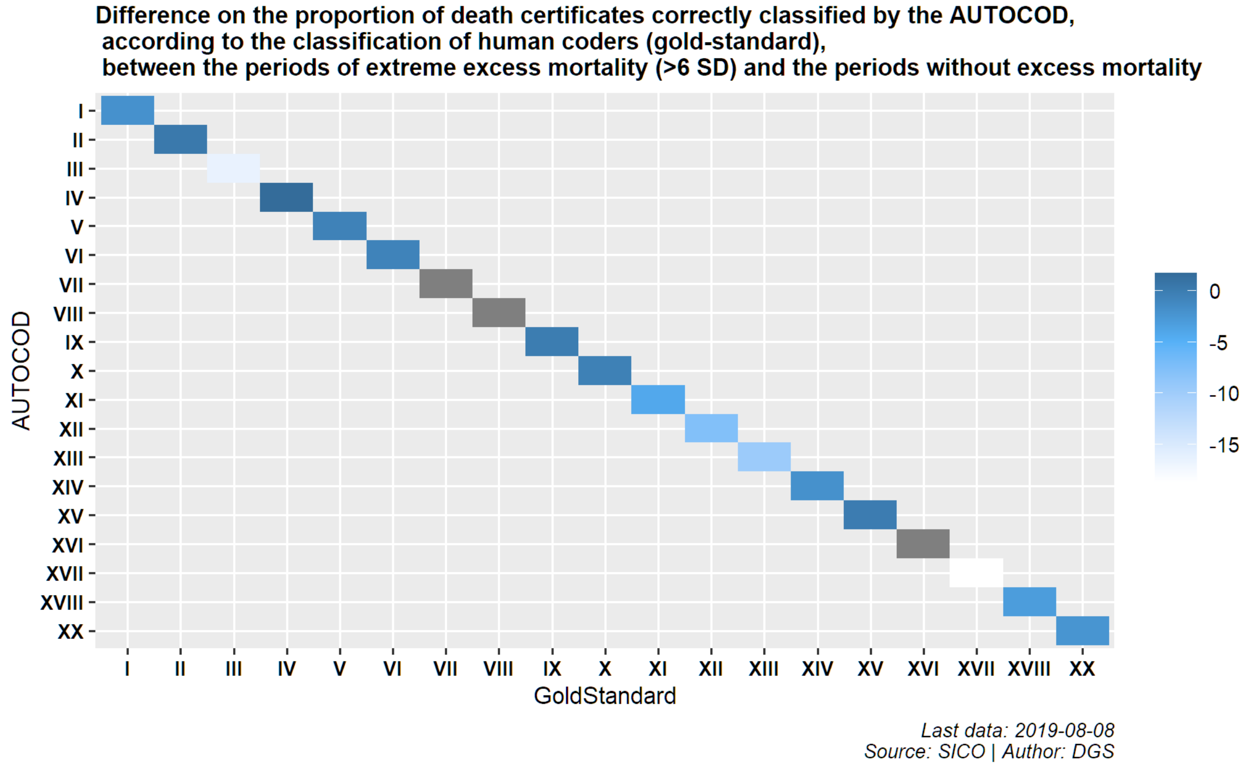

Supplementary Figure 17 - Difference on the proportion of death certificates correctly classified by the AUTOCOD, according to the classification of human coders (gold-standard), between the periods of extreme excess mortality (> 6 SDs) and the periods without excess mortality.

Caption: I - Certain infectious and parasitic diseases; II - Neoplasms; III - Diseases of the blood and blood-forming organs and certain disorders involving the immune system; IV - Endocrine, nutritional and metabolic diseases; V - Mental and behavioural disorders; VI - Diseases of the nervous system; VII - Diseases of the eye and adnexa; VIII - Diseases of the ear and mastoid process; IX - Diseases of the circulatory system; X - Diseases of the respiratory system; XI - Diseases of the digestive system; XII - Diseases of the skin and subcutaneous tissue; XIII - Diseases of the musculoskeletal system and connective tissue; XIV - Diseases of the genitourinary system; XV - Pregnancy, childbirth and the puerperium; XVI - Certain conditions originating in the perinatal period; XVII - Congenital malformations, deformations and chromosomal abnormalities; XVIII - Symptoms, signs and abnormal clinical and laboratory findings, not elsewhere specified; XIX - Injury, poisoning and certain other consequences of external causes; XX - External causes of morbidity and mortality

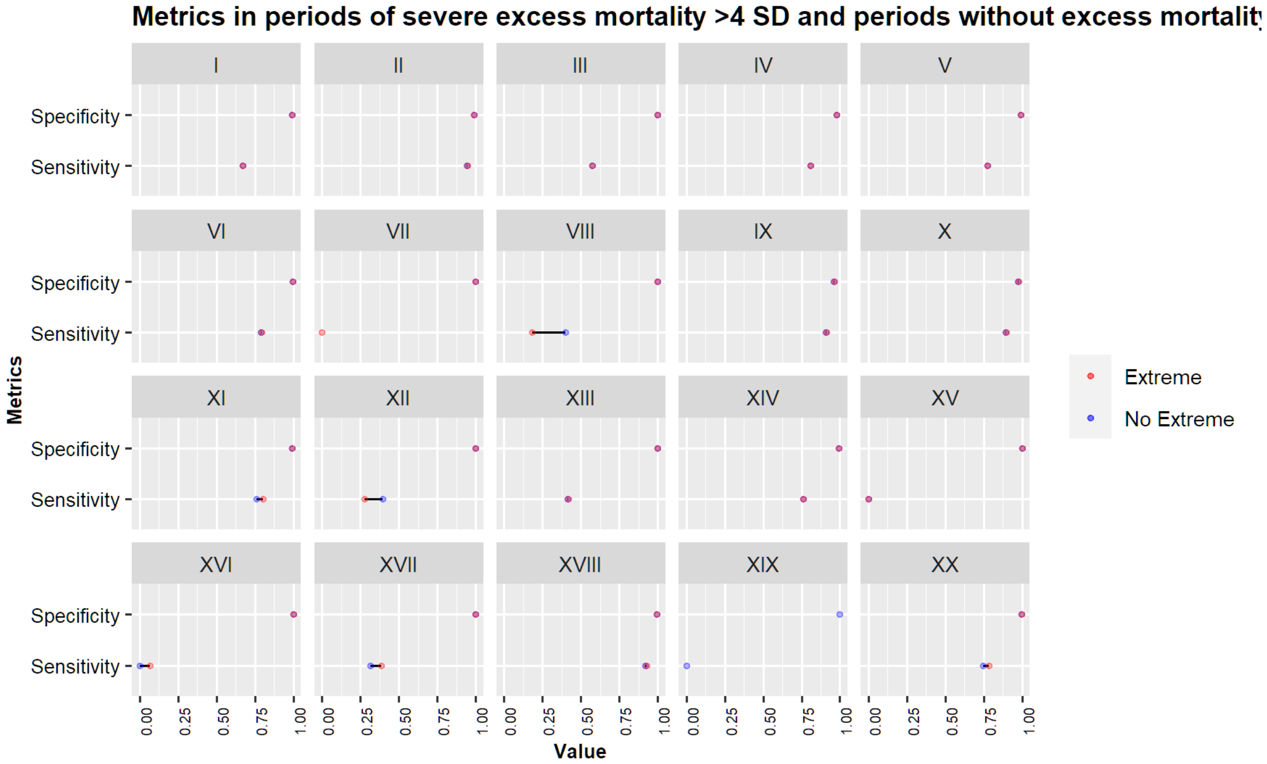

Supplementary Figure 18 - Comparison between performance metrics of AUTOCOD during periods of severe excess mortality (>4 SDs) and periods without excess mortality

Caption: I - Certain infectious and parasitic diseases; II - Neoplasms; III - Diseases of the blood and blood-forming organs and certain disorders involving the immune system; IV - Endocrine, nutritional and metabolic diseases; V - Mental and behavioural disorders; VI - Diseases of the nervous system; VII - Diseases of the eye and adnexa; VIII - Diseases of the ear and mastoid process; IX - Diseases of the circulatory system; X - Diseases of the respiratory system; XI - Diseases of the digestive system; XII - Diseases of the skin and subcutaneous tissue; XIII - Diseases of the musculoskeletal system and connective tissue; XIV - Diseases of the genitourinary system; XV - Pregnancy, childbirth and the puerperium; XVI - Certain conditions originating in the perinatal period; XVII - Congenital malformations, deformations and chromosomal abnormalities; XVIII - Symptoms, signs and abnormal clinical and laboratory findings, not elsewhere specified; XIX - Injury, poisoning and certain other consequences of external causes; XX - External causes of morbidity and mortality

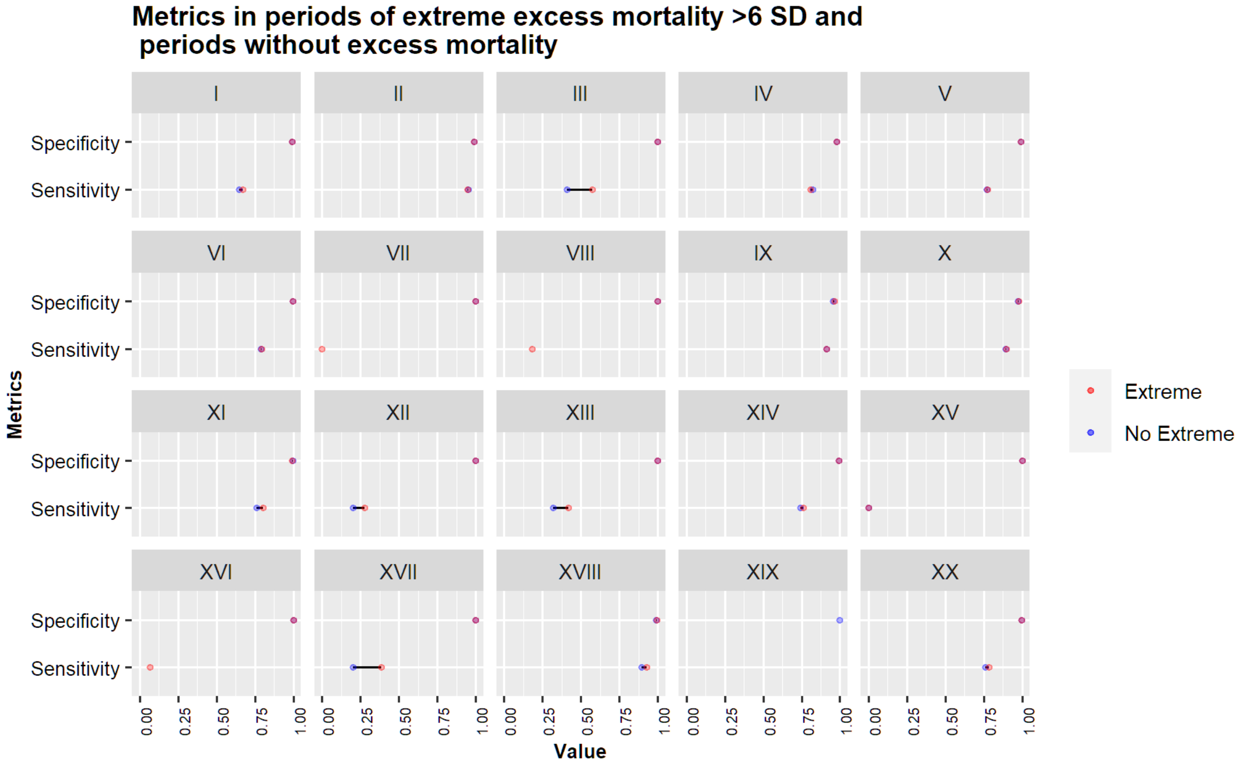

Supplementary Figure 19 - Comparison between performance metrics of AUTOCOD during periods of extreme excess mortality (>6 SDs) and periods without excess mortality

Caption: I - Certain infectious and parasitic diseases; II - Neoplasms; III - Diseases of the blood and blood-forming organs and certain disorders involving the immune system; IV - Endocrine, nutritional and metabolic diseases; V - Mental and behavioural disorders; VI - Diseases of the nervous system; VII - Diseases of the eye and adnexa; VIII - Diseases of the ear and mastoid process; IX - Diseases of the circulatory system; X - Diseases of the respiratory system; XI - Diseases of the digestive system; XII - Diseases of the skin and subcutaneous tissue; XIII - Diseases of the musculoskeletal system and connective tissue; XIV - Diseases of the genitourinary system; XV - Pregnancy, childbirth and the puerperium; XVI - Certain conditions originating in the perinatal period; XVII - Congenital malformations, deformations and chromosomal abnormalities; XVIII - Symptoms, signs and abnormal clinical and laboratory findings, not elsewhere specified; XIX - Injury, poisoning and certain other consequences of external causes; XX - External causes of morbidity and mortality

ICD-10 block analysis

Performance metrics – global overview

Supplementary Table 33 - Performance Metrics of AUTOCOD, by block, for all the periods analysed

	C00-C97				D00-D09				D10-D36				D37-D48				I05-I09			
	No Excess.	Excess Mort.	Severe >4 SD	Extreme >6 SD	No Excess.	Excess Mort.	Severe >4 SD	Extreme >6 SD	No Excess.	Excess Mort.	Severe >4 SD	Extreme >6 SD	No Excess.	Excess Mort.	Severe >4 SD	Extreme >6 SD	No Excess.	Excess Mort.	Severe >4 SD	Extreme >6 SD
Sen	0.98	0.98	0.97	0.97	0.00	NA	NA	NA	0.70	0.69	0.72	0.80	0.74	0.73	0.74	0.77	0.41	0.49	0.63	0.67
Spe	0.99	0.99	0.99	0.99	1.00	1.00	1.00	1.00	1.00	1.00	1.00	1.00	1.00	1.00	1.00	1.00	1.00	1.00	1.00	1.00
PPV	0.99	0.99	0.99	0.99	NaN	NA	NA	NA	0.76	0.84	0.93	1.00	0.83	0.82	0.76	0.54	0.51	0.53	0.57	0.50
NPV	0.99	0.99	0.99	0.99	1.00	NA	NA	NA	1.00	1.00	1.00	1.00	1.00	1.00	1.00	1.00	1.00	1.00	1.00	1.00
F1	0.99	0.98	0.98	0.98	NA	NA	NA	NA	0.73	0.76	0.81	0.89	0.78	0.77	0.75	0.63	0.45	0.51	0.60	0.57
Pre	0.40	0.34	0.30	0.29	0.00	0.00	0.00	0.00	0.00	0.00	0.00	0.00	0.01	0.01	0.01	0.00	0.00	0.00	0.00	0.00
DR	0.40	0.33	0.30	0.28	0.00	0.00	0.00	0.00	0.00	0.00	0.00	0.00	0.01	0.00	0.00	0.00	0.00	0.00	0.00	0.00
DP	0.40	0.33	0.30	0.28	0.00	0.00	0.00	0.00	0.00	0.00	0.00	0.00	0.01	0.01	0.01	0.01	0.00	0.00	0.00	0.00
BA	0.99	0.99	0.98	0.98	0.50	NA	NA	NA	0.85	0.84	0.86	0.90	0.87	0.86	0.87	0.88	0.70	0.75	0.82	0.83

	I10-I15				I20-I25				I26-I28				I30-I52				I60-I69			
	No Excess.	Excess Mort.	Severe >4 SD	Extreme >6 SD	No Excess.	Excess Mort.	Severe >4 SD	Extreme >6 SD	No Excess.	Excess Mort.	Severe >4 SD	Extreme >6 SD	No Excess.	Excess Mort.	Severe >4 SD	Extreme >6 SD	No Excess.	Excess Mort.	Severe >4 SD	Extreme >6 SD
Sen	0.85	0.86	0.87	0.87	0.93	0.92	0.92	0.92	0.80	0.79	0.77	0.80	0.91	0.92	0.92	0.92	0.94	0.94	0.93	0.93
Spe	0.99	0.99	0.99	0.99	0.99	0.99	0.99	0.99	1.00	1.00	1.00	1.00	0.99	0.99	0.98	0.99	0.99	0.99	0.99	0.99
PPV	0.76	0.76	0.78	0.78	0.94	0.93	0.95	0.93	0.81	0.82	0.80	0.78	0.90	0.90	0.89	0.90	0.95	0.95	0.96	0.96
NPV	1.00	1.00	1.00	1.00	0.99	0.99	0.99	0.99	1.00	1.00	1.00	1.00	0.99	0.99	0.99	0.99	0.99	0.99	0.99	0.99
F1	0.80	0.81	0.82	0.82	0.93	0.93	0.94	0.93	0.80	0.80	0.78	0.79	0.90	0.91	0.91	0.91	0.95	0.95	0.95	0.94
Pre	0.03	0.04	0.04	0.03	0.09	0.10	0.11	0.12	0.01	0.01	0.01	0.01	0.11	0.12	0.12	0.13	0.15	0.15	0.16	0.15
DR	0.03	0.03	0.03	0.03	0.09	0.09	0.10	0.11	0.01	0.01	0.01	0.01	0.10	0.11	0.11	0.12	0.14	0.14	0.15	0.14
DP	0.04	0.04	0.04	0.04	0.09	0.10	0.10	0.11	0.01	0.01	0.01	0.01	0.11	0.13	0.13	0.13	0.15	0.15	0.15	0.15
BA	0.92	0.92	0.93	0.93	0.96	0.96	0.96	0.96	0.90	0.89	0.88	0.90	0.95	0.95	0.95	0.95	0.97	0.97	0.96	0.96

	I70-I79				I80-I89				I95-I99				J00-J06				J09-J18			
	No Excess.	Excess Mort.	Severe >4 SD	Extreme >6 SD	No Excess.	Excess Mort.	Severe >4 SD	Extreme >6 SD	No Excess.	Excess Mort.	Severe >4 SD	Extreme >6 SD	No Excess.	Excess Mort.	Severe >4 SD	Extreme >6 SD	No Excess.	Excess Mort.	Severe >4 SD	Extreme >6 SD
Sen	0.82	0.82	0.85	0.84	0.55	0.54	0.58	0.50	0.08	0.00	0.00	NA	0.41	0.07	0.00	0.00	0.94	0.93	0.93	0.93
Spe	1.00	1.00	1.00	1.00	1.00	1.00	1.00	1.00	1.00	1.00	1.00	1.00	1.00	1.00	1.00	1.00	0.99	0.99	0.99	0.99
PPV	0.89	0.88	0.88	0.91	0.75	0.76	0.83	1.00	0.20	NaN	NA	NA	0.70	0.33	0.00	NA	0.93	0.94	0.94	0.95
NPV	1.00	1.00	1.00	1.00	1.00	1.00	1.00	1.00	1.00	1.00	1.00	NA	1.00	1.00	1.00	1.00	1.00	0.99	0.99	0.99
F1	0.85	0.85	0.86	0.87	0.64	0.63	0.68	0.67	0.11	NA	NA	NA	0.52	0.12	NA	NA	0.94	0.94	0.93	0.94
Pre	0.02	0.02	0.02	0.02	0.00	0.00	0.00	0.00	0.00	0.00	0.00	0.00	0.00	0.00	0.00	0.00	0.07	0.10	0.11	0.12
DR	0.02	0.02	0.02	0.02	0.00	0.00	0.00	0.00	0.00	0.00	0.00	0.00	0.00	0.00	0.00	0.00	0.07	0.09	0.10	0.11
DP	0.02	0.02	0.02	0.02	0.00	0.00	0.00	0.00	0.00	0.00	0.00	0.00	0.00	0.00	0.00	0.00	0.08	0.10	0.11	0.11
BA	0.91	0.91	0.92	0.92	0.78	0.77	0.79	0.75	0.54	0.50	0.50	NA	0.71	0.54	0.50	0.50	0.97	0.96	0.96	0.96

	J20-J22				J30-J39				J40-J47				J60-J70				J80-J84			
	No Excess.	Excess Mort.	Severe >4 SD	Extreme >6 SD	No Excess.	Excess Mort.	Severe >4 SD	Extreme >6 SD	No Excess.	Excess Mort.	Severe >4 SD	Extreme >6 SD	No Excess.	Excess Mort.	Severe >4 SD	Extreme >6 SD	No Excess.	Excess Mort.	Severe >4 SD	Extreme >6 SD
Sen	0.83	0.83	0.85	0.90	0.45	0.17	0.25	NA	0.89	0.89	0.90	0.89	0.87	0.84	0.84	0.82	0.82	0.81	0.79	0.88
Spe	1.00	1.00	1.00	1.00	1.00	1.00	1.00	1.00	0.99	0.99	0.99	0.99	1.00	1.00	1.00	1.00	1.00	1.00	1.00	1.00
PPV	0.87	0.86	0.89	0.93	0.53	0.25	0.50	NA	0.83	0.83	0.83	0.82	0.91	0.93	0.91	0.94	0.89	0.85	0.83	0.78
NPV	1.00	1.00	1.00	1.00	1.00	1.00	1.00	NA	1.00	1.00	0.99	0.99	1.00	1.00	1.00	1.00	1.00	1.00	1.00	1.00
F1	0.85	0.85	0.87	0.91	0.49	0.20	0.33	NA	0.86	0.86	0.86	0.86	0.89	0.88	0.88	0.87	0.85	0.83	0.81	0.82
Pre	0.01	0.01	0.01	0.02	0.00	0.00	0.00	0.00	0.04	0.05	0.05	0.06	0.01	0.01	0.01	0.01	0.01	0.01	0.01	0.01
DR	0.01	0.01	0.01	0.01	0.00	0.00	0.00	0.00	0.03	0.04	0.05	0.05	0.01	0.01	0.01	0.01	0.01	0.01	0.01	0.01
DP	0.01	0.01	0.01	0.01	0.00	0.00	0.00	0.00	0.04	0.05	0.06	0.06	0.01	0.01	0.01	0.01	0.01	0.01	0.01	0.01
BA	0.92	0.92	0.92	0.95	0.73	0.58	0.63	NA	0.94	0.94	0.95	0.94	0.93	0.92	0.92	0.91	0.91	0.91	0.89	0.94

	J85-J86				J90-J94				J95-J99			
	No Excess.	Excess Mort.	Severe >4 SD	Extreme >6 SD	No Excess.	Excess Mort.	Severe >4 SD	Extreme >6 SD	No Excess.	Excess Mort.	Severe >4 SD	Extreme >6 SD
Sen	0.31	0.35	0.08	0.00	0.66	0.69	0.71	0.50	0.92	0.92	0.92	0.91
Spe	1.00	1.00	1.00	1.00	1.00	1.00	1.00	1.00	1.00	0.99	0.99	0.99
PPV	0.83	0.90	0.50	NA	0.63	0.59	0.71	0.67	0.85	0.84	0.85	0.83
NPV	1.00	1.00	1.00	1.00	1.00	1.00	1.00	1.00	1.00	1.00	1.00	1.00
F1	0.46	0.51	0.14	NA	0.65	0.64	0.71	0.57	0.88	0.88	0.88	0.87
Pre	0.00	0.00	0.00	0.00	0.00	0.00	0.00	0.00	0.03	0.03	0.04	0.04
DR	0.00	0.00	0.00	0.00	0.00	0.00	0.00	0.00	0.02	0.03	0.03	0.03
DP	0.00	0.00	0.00	0.00	0.00	0.00	0.00	0.00	0.03	0.04	0.04	0.04
BA	0.66	0.68	0.54	0.50	0.83	0.84	0.85	0.75	0.96	0.96	0.96	0.95

Caption: Sen – Sensitivity; Spe – Specificity; PPV – Positive Predictive Value; NPB – Negative Predictive Value; F1 – F1-score; Pre – Prevalence; DR – Detection Rate; DP – Detection Prevalence; BA – Balanced Accuracy; C00-C97 - Malignant neoplasms; D00-D09 - In situ neoplasms; D10-D36 - Benign neoplasms; D37-D48 - Neoplasms of uncertain or unknown behaviour; I05-I09 - Chronic rheumatic heart diseases; I10-I15 - Hypertensive diseases; I20-I25 - Ischaemic heart diseases; I26-I28 - Pulmonary heart disease and diseases of pulmonary circulation; I30-I52 - Other forms of heart disease; I60-I69 - Cerebrovascular diseases; I70-I79 - Diseases of arteries, arterioles and capillaries; I80-I89 - Diseases of veins, lymphatic vessels and lymph nodes, not elsewhere classified; I95-I99 - Other and unspecified disorders of the circulatory system; J00-J06 - Acute upper respiratory infections; J09-J18 - Influenza and pneumonia; J20-J22 - Other acute lower respiratory infections; J30-J39 - Other diseases of upper respiratory tract; J40-J47 - Chronic lower respiratory diseases; J60-J70 - Lung diseases due to external agents; J80-J84 - Other respiratory diseases principally

affecting the interstitium; J85-J86 - Suppurative and necrotic conditions of lower respiratory tract; J90-J94 - Other diseases of pleura; J95-J99 - Other diseases of the respiratory system

Supplementary Table 34 - Weighted-averages for performance metrics for different periods, for ICD-10's block classification of AUTOCOD

Block	Period	Sensitivity	Specificity	Pos Pred Value	Neg Pred Value	F1	Prevalence	Detection Rate	Detection Prevalence	Balanced Accuracy
**Weighted-average**	No Excess Mort.	0.94	0.99	0.93	0.99	0.93	0.20	0.20	0.20	0.96
	Excess Mort.	0.94	0.99	0.94	0.99	0.94	0.21	0.21	0.21	0.97
	Severe +4SD	0.93	0.99	0.93	0.99	0.93	0.17	0.17	0.17	0.96
	Extreme +6SD	0.93	0.99	0.93	0.99	0.93	0.16	0.15	0.16	0.96

All periods

Supplementary Table 35 - Distribution of death certificates in the dataset, according to year of death and to ICD-10 block (descendent order, 50 most common blocks), for all periods

Chapter	Block	Block Description	N	%
**II**	**C00-C97**	Malignant neoplasms	84 031	25.46
**IX**	**I60-I69**	Cerebrovascular diseases	34 595	10.48
**IX**	**I30-I52**	Other forms of heart disease	26 016	7.88
**IX**	**I20-I25**	Ischaemic heart diseases	21 153	6.41
**X**	**J09-J18**	Influenza and pneumonia	18 191	5.51
**IV**	**E10-E14**	Diabetes mellitus	12 731	3.86
**V**	**F00-F09**	Organic, including symptomatic, mental disorders	12 150	3.68
**X**	**J40-J47**	Chronic lower respiratory diseases	8 953	2.71
**XX**	**V01-X59**	Accidents	8 509	2.58
**IX**	**I10-I15**	Hypertensive diseases	7 611	2.31
**X**	**J95-J99**	Other diseases of the respiratory system	6 445	1.95
**XVIII**	**R50-R69**	General symptoms and signs	6 022	1.82
**XVIII**	**R00-R09**	Symptoms and signs involving the circulatory and respiratory systems	5 799	1.76
**VI**	**G30-G32**	Other degenerative diseases of the nervous system	5 428	1.64
**IX**	**I70-I79**	Diseases of arteries, arterioles and capillaries	4 794	1.45
**XI**	**K70-K77**	Diseases of liver	4 574	1.39
**XIV**	**N30-N39**	Other diseases of the urinary system	4 461	1.35
**XVIII**	**R95-R99**	Ill-defined and unknown cause of mortality	4 385	1.33
**XI**	**K55-K64**	Other diseases of intestines	4 218	1.28
**XIV**	**N17-N19**	Acute kidney failure and chronic kidney disease	3 998	1.21
**I**	**A30-A49**	Other bacterial diseases	3 066	0.93
**VI**	**G20-G26**	Extrapyramidal and movement disorders	2 862	0.87
**XX**	**X60-X84**	Intentional self-harm	2 808	0.85
**XI**	**K80-K87**	Disorders of gallbladder, biliary tract and pancreas	2 345	0.71
**X**	**J60-J70**	Lung diseases due to external agents	2 340	0.71
**IX**	**I26-I28**	Pulmonary heart disease and diseases of pulmonary circulation	2 314	0.70
**IV**	**E70-E90**	Metabolic disorders	2 302	0.70
**X**	**J20-J22**	Other acute lower respiratory infections	2 102	0.64
**XX**	**Y10-Y34**	Event of undetermined intent	1 819	0.55
**X**	**J80-J84**	Other respiratory diseases principally affecting the interstitium	1 636	0.50
**II**	**D37-D48**	Neoplasms of uncertain or unknown behaviour	1 486	0.45
**VI**	**G40-G47**	Episodic and paroxysmal disorders	1 105	0.33
**XI**	**K90-K93**	Other diseases of the digestive system	1 049	0.32
**XIV**	**N10-N16**	Renal tubulo-interstitial diseases	1 032	0.31
**I**	**B20-B24**	Human immunodeficiency virus [HIV] disease	961	0.29
**VI**	**G10-G14**	Systemic atrophies primarily affecting the central nervous system	942	0.29
**XI**	**K20-K31**	Diseases of oesophagus, stomach and duodenum	921	0.28
**IV**	**E65-E68**	Obesity and other hyperalimentation	758	0.23
**I**	**A00-A09**	Intestinal infectious diseases	682	0.21
**NA**	**D60-D64**	Aplastic and other anaemias	644	0.20
**XI**	**K50-K52**	Noninfective enteritis and colitis	634	0.19
**XI**	**K65-K67**	Diseases of peritoneum	605	0.18
**IX**	**I05-I09**	Chronic rheumatic heart diseases	490	0.15
**XI**	**K40-K46**	Hernia	477	0.14
**XX**	**Y40-Y84**	Complications of medical and surgical care	475	0.14
**XIII**	**M00-M25**	Arthropathies	434	0.13
**IX**	**I80-I89**	Diseases of veins, lymphatic vessels and lymph nodes, not elsewhere classified	427	0.13
**VI**	**G90-G99**	Other disorders of the nervous system	398	0.12
**XIII**	**M30-M36**	Systemic connective tissue disorders	359	0.11
**I**	**A15-A19**	Tuberculosis	345	0.10

Supplementary Table 36 - Distribution of death certificates in the dataset, according to ICD-10 block (descendent order, for the three most common chapters – II, IX and X), for all periods

Chapter	Block	Block Description	N	%
**II**	C00-C97	Malignant neoplasms	84 031	37.60
**IX**	I60-I69	Cerebrovascular diseases	34 595	15.48
**IX**	I30-I52	Other forms of heart disease	26 016	11.64
**IX**	I20-I25	Ischaemic heart diseases	21 153	9.47
**X**	J09-J18	Influenza and pneumonia	18 191	8.14
**X**	J40-J47	Chronic lower respiratory diseases	8 953	4.01
**IX**	I10-I15	Hypertensive diseases	7 611	3.41
**X**	J95-J99	Other diseases of the respiratory system	6 445	2.88
**IX**	I70-I79	Diseases of arteries, arterioles and capillaries	4 794	2.15
**X**	J60-J70	Lung diseases due to external agents	2 340	1.05
**IX**	I26-I28	Pulmonary heart disease and diseases of pulmonary circulation	2 314	1.04
**X**	J20-J22	Other acute lower respiratory infections	2 102	0.94
**X**	J80-J84	Other respiratory diseases principally affecting the interstitium	1 636	0.73
**II**	D37-D48	Neoplasms of uncertain or unknown behaviour	1 486	0.66
**IX**	I05-I09	Chronic rheumatic heart diseases	490	0.22
**IX**	I80-I89	Diseases of veins, lymphatic vessels and lymph nodes, not elsewhere classified	427	0.19
**II**	D10-D36	Benign neoplasms	311	0.14
**X**	J90-J94	Other diseases of pleura	221	0.10
**X**	J85-J86	Suppurative and necrotic conditions of lower respiratory tract	215	0.10
**X**	J30-J39	Other diseases of upper respiratory tract	53	0.02
**X**	J00-J06	Acute upper respiratory infections	46	0.02
**IX**	I95-I99	Other and unspecified disorders of the circulatory system	20	0.01
**II**	D00-D09	In situ neoplasms	9	0.00

Supplementary Table 37 - Distribution of death certificates in the dataset, according to ICD-10 block (descendent order, for the three most common chapters – II, IX and X), for all periods

Year of Death	Chapter	Block	Block Description	N	%
2018	II	C00-C97	Malignant neoplasms	27 710	12.40
2016	II	C00-C97	Malignant neoplasms	27 374	12.25
2017	II	C00-C97	Malignant neoplasms	27 286	12.21
2016	IX	I60-I69	Cerebrovascular diseases	11 722	5.25
2017	IX	I60-I69	Cerebrovascular diseases	11 096	4.97
2018	IX	I60-I69	Cerebrovascular diseases	11 019	4.93
2018	IX	I30-I52	Other forms of heart disease	8 564	3.83
2016	IX	I30-I52	Other forms of heart disease	8 509	3.81
2017	IX	I30-I52	Other forms of heart disease	8 276	3.70
2016	IX	I20-I25	Ischaemic heart diseases	7 359	3.29
2017	IX	I20-I25	Ischaemic heart diseases	6 772	3.03
2018	IX	I20-I25	Ischaemic heart diseases	6 513	2.91
2016	X	J09-J18	Influenza and pneumonia	6 129	2.74
2018	X	J09-J18	Influenza and pneumonia	5 915	2.65
2017	X	J09-J18	Influenza and pneumonia	5 686	2.54
2016	X	J40-J47	Chronic lower respiratory diseases	3 006	1.35
2018	X	J40-J47	Chronic lower respiratory diseases	2 977	1.33
2017	X	J40-J47	Chronic lower respiratory diseases	2 762	1.24
2018	IX	I10-I15	Hypertensive diseases	2 631	1.18
2016	IX	I10-I15	Hypertensive diseases	2 430	1.09
2017	IX	I10-I15	Hypertensive diseases	2 348	1.05
2018	X	J95-J99	Other diseases of the respiratory system	2 148	0.96
2017	X	J95-J99	Other diseases of the respiratory system	2 097	0.94
2016	X	J95-J99	Other diseases of the respiratory system	2 037	0.91
2019	II	C00-C97	Malignant neoplasms	1 661	0.74
2016	IX	I70-I79	Diseases of arteries, arterioles and capillaries	1 610	0.72
2018	IX	I70-I79	Diseases of arteries, arterioles and capillaries	1 558	0.70
2017	IX	I70-I79	Diseases of arteries, arterioles and capillaries	1 543	0.69
2018	X	J60-J70	Lung diseases due to external agents	830	0.37
2016	X	I26-I28	Pulmonary heart disease and diseases of pulmonary circulation	829	0.37
2016	X	J20-J22	Other acute lower respiratory infections	786	0.35
2017	X	I26-I28	Pulmonary heart disease and diseases of pulmonary circulation	783	0.35
2019	IX	I60-I69	Cerebrovascular diseases	758	0.34
2016	X	J60-J70	Lung diseases due to external agents	733	0.33
2017	X	J60-J70	Lung diseases due to external agents	728	0.33
2017	X	J20-J22	Other acute lower respiratory infections	705	0.32
2019	IX	I30-I52	Other forms of heart disease	667	0.30
2018	X	I26-I28	Pulmonary heart disease and diseases of pulmonary circulation	661	0.30
2016	X	J80-J84	Other respiratory diseases principally affecting the interstitium	590	0.26
2018	X	J20-J22	Other acute lower respiratory infections	587	0.26
2016	II	D37-D48	Neoplasms of uncertain or unknown behaviour	510	0.23
2019	IX	I20-I25	Ischaemic heart diseases	509	0.23
2018	X	J80-J84	Other respiratory diseases principally affecting the interstitium	504	0.23

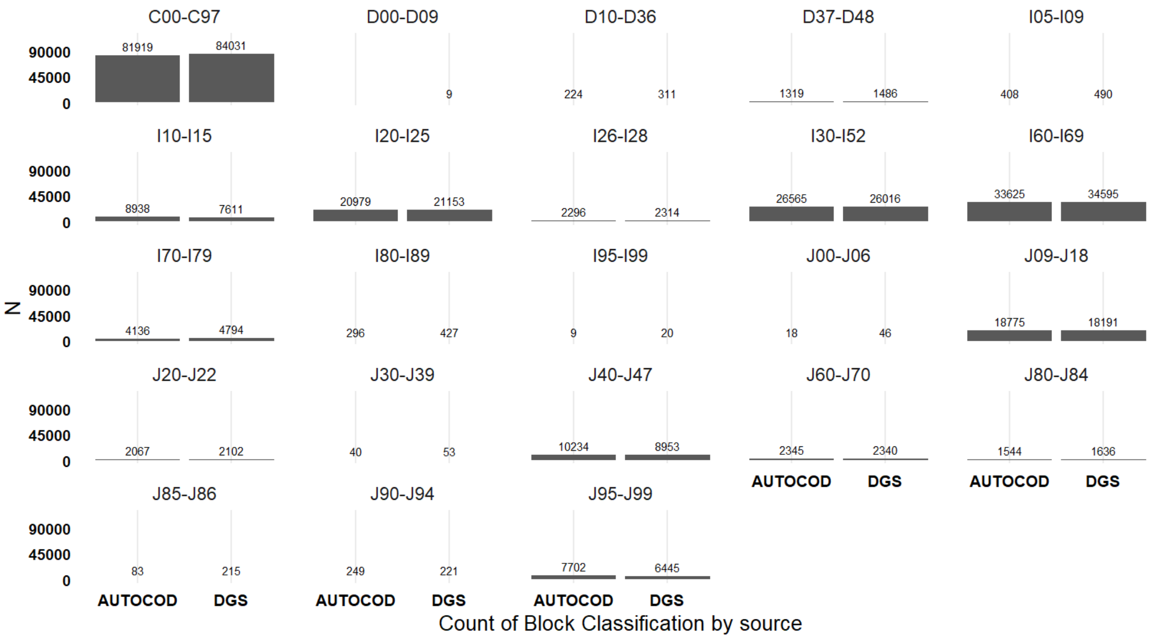

Supplementary Figure 20 - Count of death certificates classified by ICD-10 block either by human coders (DGS) or by AUTOCOD, in all periods

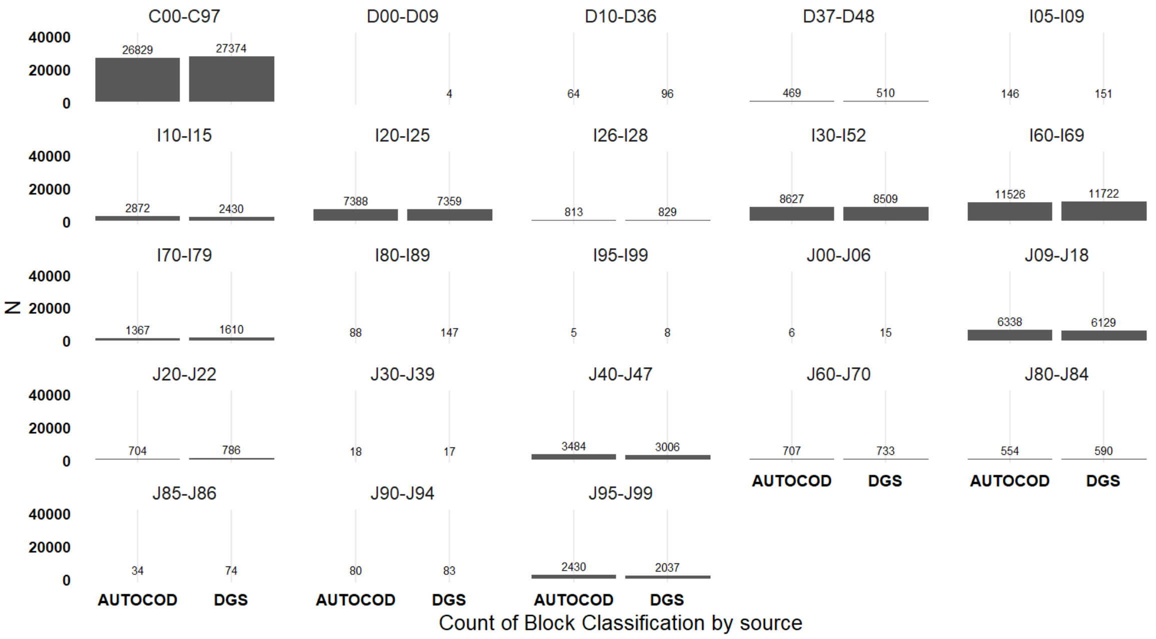

Supplementary Figure 21 - Count of death certificates classified by ICD-10 block either by human coders (DGS) or by AUTOCOD, in 2016

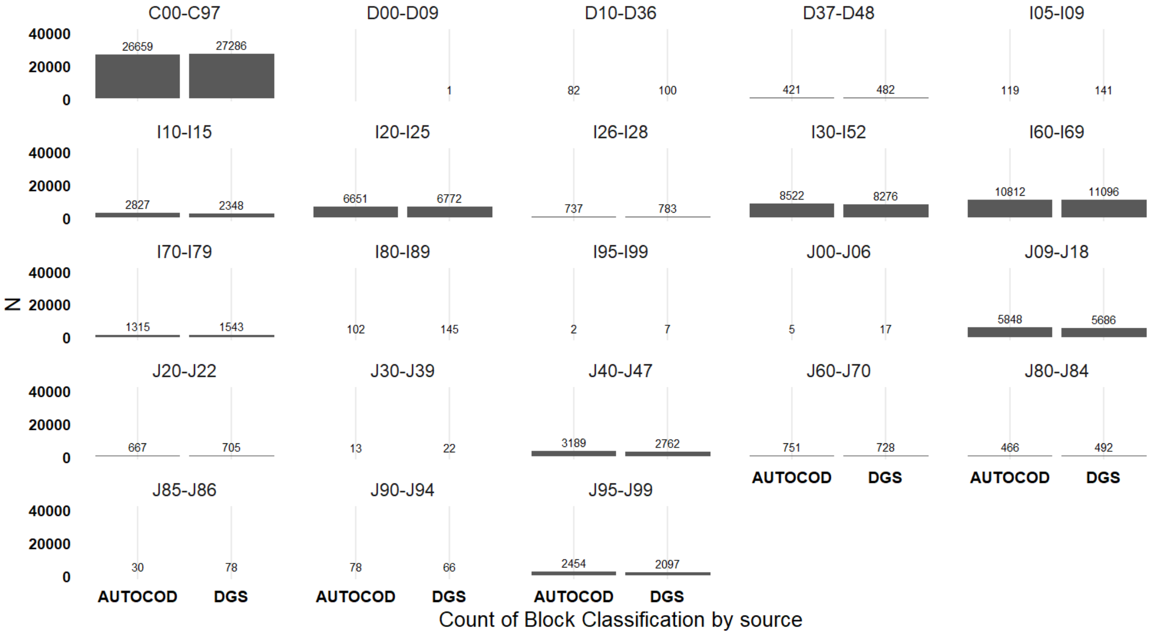

Supplementary Figure 22 - Count of death certificates classified by ICD-10 block either by human coders (DGS) or by AUTOCOD, in 2017

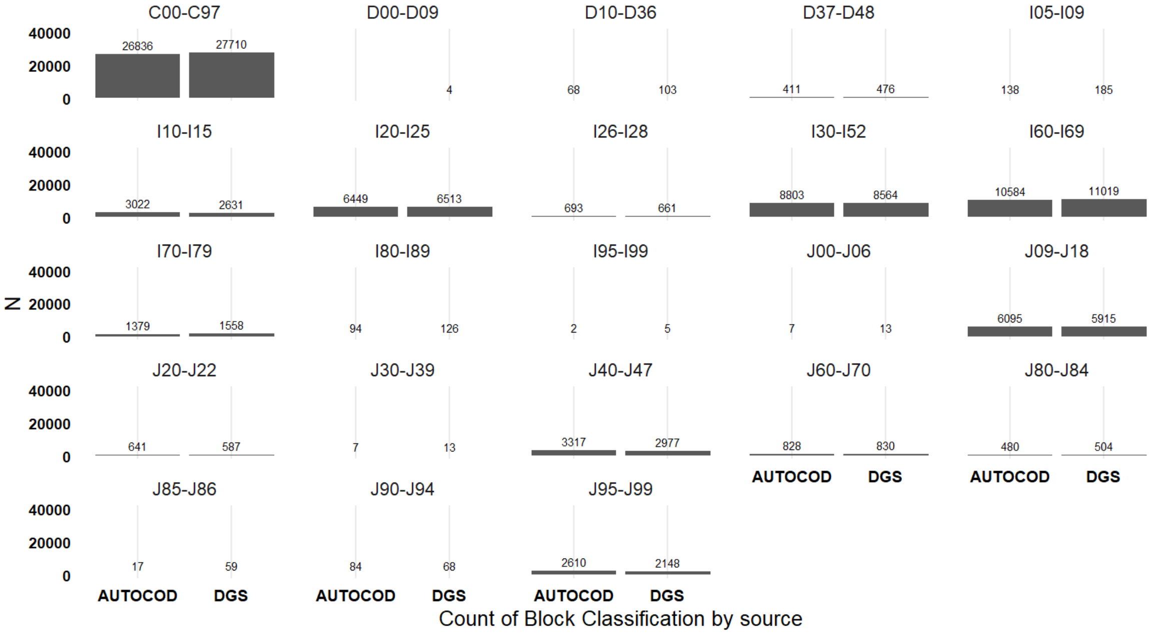

Supplementary Figure 23 - Count of death certificates classified by ICD-10 block either by human coders (DGS) or by AUTOCOD, in 2018

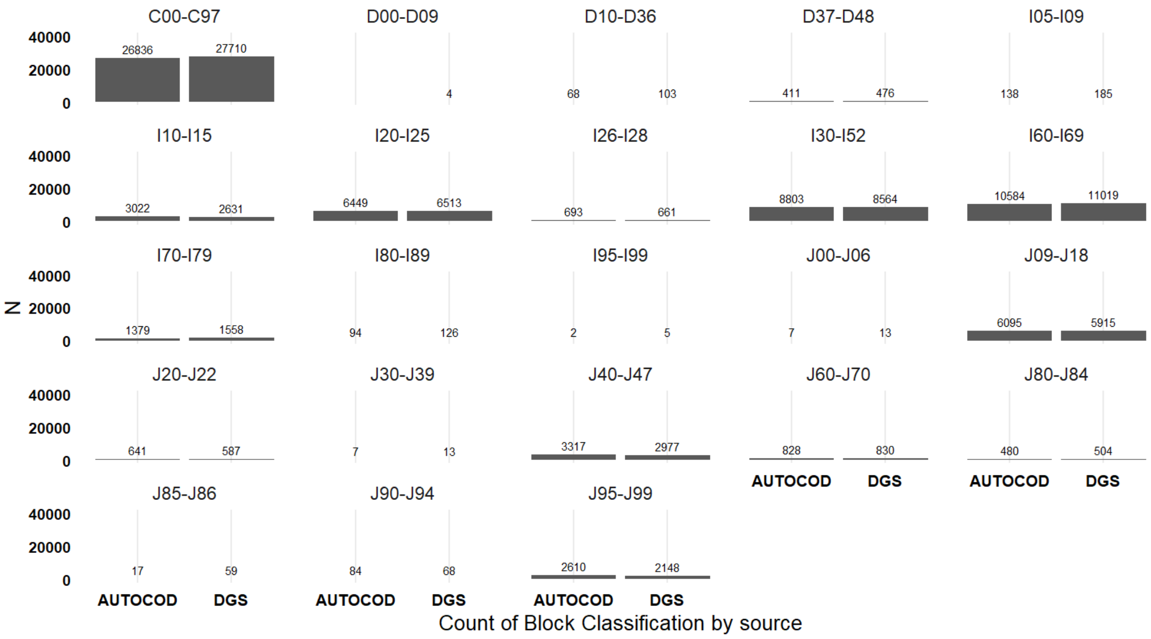

Supplementary Figure 24 - Count of death certificates classified by ICD-10 block either by human coders (DGS) or by AUTOCOD, in 2019

Supplementary Table 38 - Confusion matrix for ICD-10 block classification, all periods

	C00-C97	D00-D09	D10-D36	D37-D48	I05-I09	I10-I15	I20-I25	I26-I28	I30-I52	I60-I69	I70-I79	I80-I89	I95-I99	J00-J06	J09-J18	J20-J22	J30-J39	J40-J47	J60-J70	J80-J84	J85-J86	J90-J94	J95-J99
C00-C97	79 941	3	29	181	3	44	67	15	110	112	23	17	0	1	63	15	1	42	17	12	1	4	17
D00-D09	0	0	0	0	0	0	0	0	0	0	0	0	0	0	0	0	0	0	0	0	0	0	0
D10-D36	23	0	162	3	0	0	0	0	1	8	0	0	0	0	9	0	0	1	0	1	0	0	1
D37-D48	151	0	0	983	2	3	2	0	5	11	1	0	0	0	11	0	1	9	0	4	0	1	8
I05-I09	5	0	0	0	191	9	15	0	135	8	2	1	0	0	2	0	0	2	1	0	0	0	1
I10-I15	104	0	5	8	12	5 830	368	83	301	470	227	13	0	0	55	1	0	89	29	24	0	4	54
I20-I25	105	1	4	10	18	94	18 661	72	504	203	82	18	2	0	43	9	1	75	3	9	2	4	31
I26-I28	50	0	1	5	8	29	36	1 718	148	14	21	16	0	0	8	0	0	28	2	14	0	5	11
I30-I52	201	0	0	14	198	461	532	121	22 399	313	250	21	3	0	78	19	0	181	13	30	11	19	85
I60-I69	191	0	17	30	8	127	177	23	413	30 518	94	14	2	2	193	131	5	106	43	15	8	5	83
I70-I79	70	0	0	2	1	76	54	16	59	77	3 363	28	6	0	10	2	0	2	1	0	2	0	8
I80-I89	14	0	1	0	0	2	3	9	8	10	5	177	1	0	2	0	0	0	0	0	1	2	1
I95-I99	0	0	0	0	0	0	0	1	0	1	2	0	1	0	0	0	0	0	0	0	0	0	0
J00-J06	1	0	0	0	0	0	0	0	0	1	0	0	0	8	0	0	0	0	0	0	0	0	3
J09-J18	289	0	6	30	3	38	101	32	108	215	25	1	1	2	16 062	11	1	134	40	30	43	9	49
J20-J22	32	0	0	1	1	3	3	0	12	52	5	0	0	1	12	1 660	0	103	2	1	1	0	21
J30-J39	0	0	0	1	1	0	0	0	1	5	0	0	0	0	0	0	10	3	0	0	0	0	0
J40-J47	233	0	1	52	0	58	80	36	177	186	7	4	0	3	372	99	3	7 633	64	71	42	5	94
J60-J70	27	1	0	3	0	7	2	1	10	53	3	0	0	0	36	0	2	8	1 842	5	0	1	6
J80-J84	26	0	1	1	0	8	16	10	33	1	0	2	0	0	24	4	1	14	25	1 245	1	0	9
J85-J86	5	0	0	0	0	0	0	2	2	0	0	0	0	0	1	0	0	0	0	1	60	0	0
J90-J94	21	0	0	3	0	1	1	0	25	3	0	1	0	0	12	5	0	0	4	1	0	126	0
J95-J99	164	0	5	5	0	39	56	21	139	134	14	10	1	14	119	42	1	128	55	54	13	3	5 508

Caption: C00-C97 - Malignant neoplasms; D00-D09 - In situ neoplasms; D10-D36 - Benign neoplasms; D37-D48 - Neoplasms of uncertain or unknown behaviour; I05-I09 - Chronic rheumatic heart diseases; I10-I15 - Hypertensive diseases; I20-I25 - Ischaemic heart diseases; I26-I28 - Pulmonary heart disease and diseases of pulmonary circulation; I30-I52 - Other forms of heart disease; I60-I69 - Cerebrovascular diseases; I70-I79 - Diseases of arteries, arterioles and capillaries; I80-I89 - Diseases of veins, lymphatic vessels and lymph nodes, not elsewhere classified; I95-I99 - Other and unspecified disorders of the circulatory system; J00-J06 - Acute upper respiratory infections; J09-J18 - Influenza and pneumonia; J20-J22 - Other acute lower respiratory infections; J30-J39 - Other diseases of upper respiratory tract; J40-J47 - Chronic lower respiratory diseases; J60-J70 - Lung diseases due to external agents; J80-J84 - Other respiratory diseases principally affecting the interstitium; J85-J86 - Suppurative and necrotic conditions of lower respiratory tract; J90-J94 - Other diseases of pleura; J95-J99 - Other diseases of the respiratory system

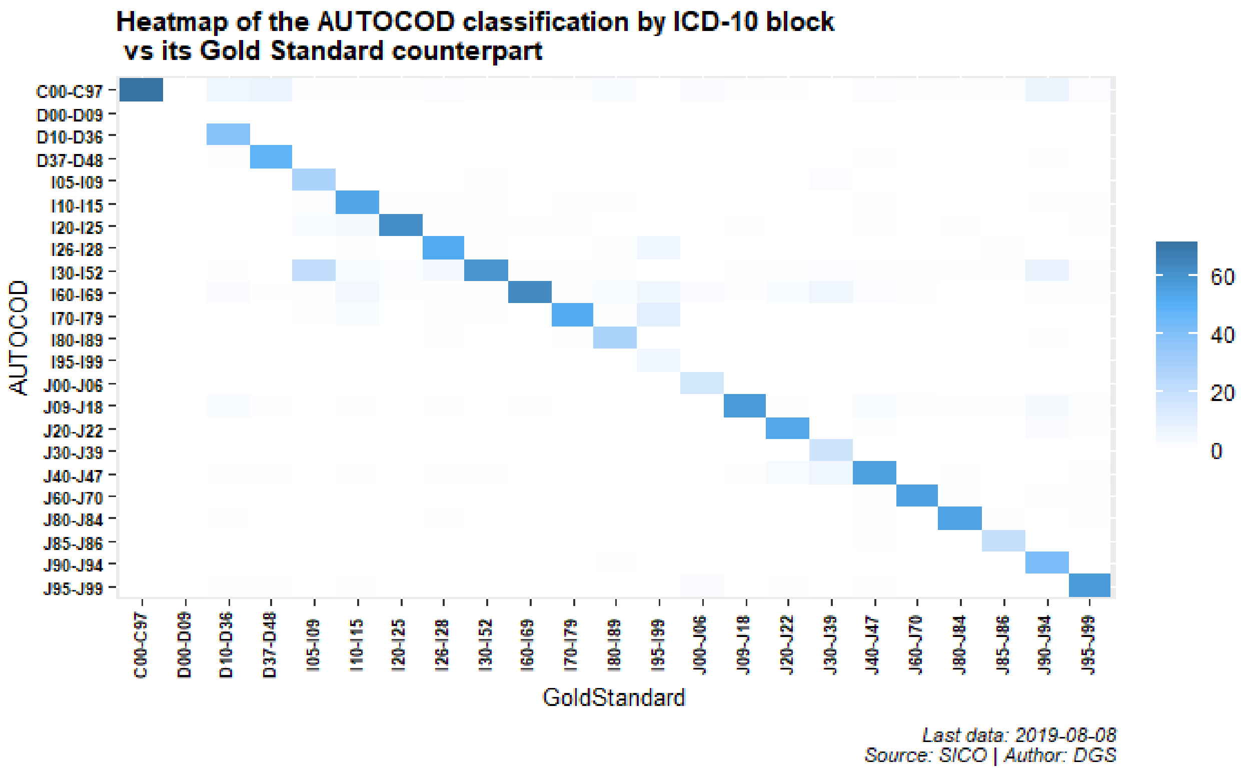

Supplementary Figure 25 - Heatmap of the AUTOCOD classification by ICD-10 block vs its Gold Standard counterpart. Values on the diagonal represent correct classifications, during all period

Caption: C00-C97 - Malignant neoplasms; D00-D09 - In situ neoplasms; D10-D36 - Benign neoplasms; D37-D48 - Neoplasms of uncertain or unknown behaviour; I05-I09 - Chronic rheumatic heart diseases; I10-I15 - Hypertensive diseases; I20-I25 - Ischaemic heart diseases; I26-I28 - Pulmonary heart disease and diseases of pulmonary circulation; I30-I52 - Other forms of heart disease; I60-I69 - Cerebrovascular diseases; I70-I79 - Diseases of arteries, arterioles and capillaries; I80-I89 - Diseases of veins, lymphatic vessels and lymph nodes, not elsewhere classified; I95-I99 - Other and unspecified disorders of the circulatory system; J00-J06 - Acute upper respiratory infections; J09-J18 - Influenza and pneumonia; J20-J22 - Other acute lower respiratory infections; J30-J39 - Other diseases of upper respiratory tract; J40-J47 - Chronic lower respiratory diseases; J60-J70 - Lung diseases due to external agents; J80-J84 - Other respiratory diseases principally affecting the interstitium; J85-J86 - Suppurative and necrotic conditions of lower respiratory tract; J90-J94 - Other diseases of pleura; J95-J99 - Other diseases of the respiratory system

Supplementary Table 39 - Overall performance metrics of AUTOCOD when classifying by ICD-10 block, all periods

**Accuracy**	0.93
**Kappa**	0.92
**AccuracyLower**	0.93
**AccuracyUpper**	0.94
**AccuracyNull**	0.39
**AccuracyPValue**	0.00

Supplementary Table 40 - Performance Metrics of AUTOCOD by ICD-10 block, all periods

Block	Sensitivity	Specificity	Pos Pred Value	Neg Pred Value	F1	Prevalence	Detection Rate	Detection Prevalence	Balanced Accuracy
**C00-C97**	0.98	0.99	0.99	0.99	0.99	0.39	0.38	0.38	0.99
**D00-D09**	0.00	1.00	NaN	1.00	NA	0.00	0.00	0.00	0.50
**D10-D36**	0.70	1.00	0.78	1.00	0.74	0.00	0.00	0.00	0.85
**D37-D48**	0.74	1.00	0.83	1.00	0.78	0.01	0.01	0.01	0.87
**I05-I09**	0.43	1.00	0.51	1.00	0.47	0.00	0.00	0.00	0.71
**I10-I15**	0.85	0.99	0.76	1.00	0.80	0.03	0.03	0.04	0.92
**I20-I25**	0.93	0.99	0.94	0.99	0.93	0.10	0.09	0.09	0.96
**I26-I28**	0.80	1.00	0.81	1.00	0.80	0.01	0.01	0.01	0.90
**I30-I52**	0.91	0.99	0.90	0.99	0.90	0.12	0.11	0.12	0.95
**I60-I69**	0.94	0.99	0.95	0.99	0.95	0.15	0.14	0.15	0.97
**I70-I79**	0.82	1.00	0.89	1.00	0.85	0.02	0.02	0.02	0.91
**I80-I89**	0.55	1.00	0.75	1.00	0.63	0.00	0.00	0.00	0.77
**I95-I99**	0.06	1.00	0.20	1.00	0.09	0.00	0.00	0.00	0.53
**J00-J06**	0.26	1.00	0.62	1.00	0.36	0.00	0.00	0.00	0.63
**J09-J18**	0.94	0.99	0.93	1.00	0.94	0.08	0.08	0.08	0.97
**J20-J22**	0.83	1.00	0.87	1.00	0.85	0.01	0.01	0.01	0.92
**J30-J39**	0.39	1.00	0.48	1.00	0.43	0.00	0.00	0.00	0.69
**J40-J47**	0.89	0.99	0.83	1.00	0.86	0.04	0.04	0.04	0.94
**J60-J70**	0.86	1.00	0.92	1.00	0.89	0.01	0.01	0.01	0.93
**J80-J84**	0.82	1.00	0.88	1.00	0.85	0.01	0.01	0.01	0.91
**J85-J86**	0.32	1.00	0.85	1.00	0.47	0.00	0.00	0.00	0.66
**J90-J94**	0.67	1.00	0.62	1.00	0.65	0.00	0.00	0.00	0.84
**J95-J99**	0.92	1.00	0.84	1.00	0.88	0.03	0.03	0.03	0.96

Caption: C00-C97 - Malignant neoplasms; D00-D09 - In situ neoplasms; D10-D36 - Benign neoplasms; D37-D48 - Neoplasms of uncertain or unknown behaviour; I05-I09 - Chronic rheumatic heart diseases; I10-I15 - Hypertensive diseases; I20-I25 - Ischaemic heart diseases; I26-I28 - Pulmonary heart disease and diseases of pulmonary circulation; I30-I52 - Other forms of heart disease; I60-I69 - Cerebrovascular diseases; I70-I79 - Diseases of arteries, arterioles and capillaries; I80-I89 - Diseases of veins, lymphatic vessels and lymph nodes, not elsewhere classified; I95-I99 - Other and unspecified disorders of the circulatory system; J00-J06 - Acute upper respiratory infections; J09-J18 - Influenza and pneumonia; J20-J22 - Other acute lower respiratory infections; J30-J39 - Other diseases of upper respiratory tract; J40-J47 - Chronic lower respiratory diseases; J60-J70 - Lung diseases due to external agents; J80-J84 - Other respiratory diseases principally affecting the interstitium; J85-J86 - Suppurative and necrotic conditions of lower respiratory tract; J90-J94 - Other diseases of pleura; J95-J99 - Other diseases of the respiratory system

Periods of excess mortality

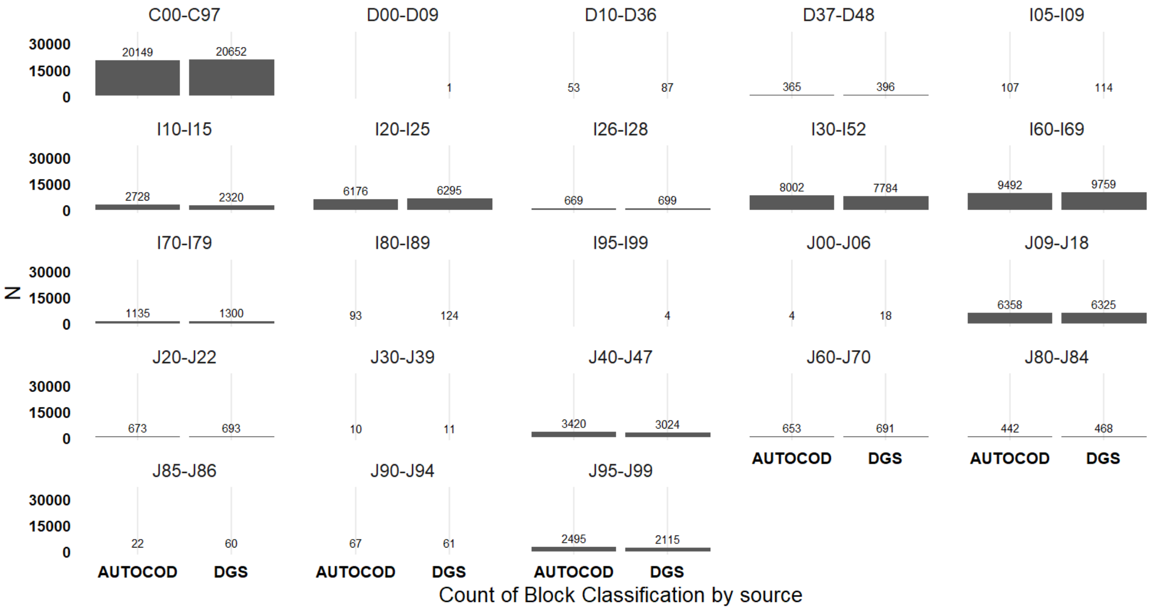

Supplementary Figure 26 - Count of death certificates classified by ICD-10 block either by human coders (DGS) or by AUTOCOD, in periods of excess mortality

Supplementary Table 41 - Confusion matrix for ICD-10 block classification, excess mortality periods

		Human Coders (GOLD-STANDARD)																						
		C00-C97	D00-D09	D10-D36	D37-D48	I05-I09	I10-I15	I20-I25	I26-I28	I30-I52	I60-I69	I70-I79	I80-I89	I95-I99	J00-J06	J09-J18	J20-J22	J30-J39	J40-J47	J60-J70	J80-J84	J85-J86	J90-J94	J95-J99
AUTOCOD	C00-C97	#####	0	9	53	0	12	27	5	35	41	9	6	0	0	26	5	1	13	6	1	0	1	4
	D00-D09	0	0	0	0	0	0	0	0	0	0	0	0	0	0	0	0	0	0	0	0	0	0	0
	D10-D36	6	0	42	0	0	0	0	0	0	1	0	0	0	0	1	0	0	0	0	0	0	0	0
	D37-D48	47	0	0	267	1	1	0	0	1	4	0	0	0	0	3	0	0	0	0	0	0	0	3
	I05-I09	2	0	0	0	52	4	2	0	32	1	0	0	0	0	2	0	0	2	1	0	0	0	1
	I10-I15	34	0	1	1	4	1,775	117	27	88	135	70	6	0	0	18	1	0	28	7	4	0	1	18
	I20-I25	28	0	0	3	4	28	5,493	22	144	68	23	5	1	0	18	3	1	31	1	1	2	0	6
	I26-I28	12	0	0	0	1	9	12	513	41	4	5	5	0	0	0	0	0	10	2	5	0	1	5
	I30-I52	52	0	0	4	41	132	161	39	6,761	104	56	10	0	0	26	8	0	73	5	10	3	7	32
	I60-I69	47	0	4	7	1	32	56	3	90	8,602	29	3	1	1	68	38	1	27	13	5	2	3	18
	I70-I79	23	0	0	0	1	24	14	6	17	22	908	8	2	0	2	1	0	0	1	0	0	0	2
	I80-I89	3	0	1	0	0	0	3	5	2	1	1	56	0	0	1	0	0	0	0	0	1	0	0
	I95-I99	0	0	0	0	0	0	0	0	0	0	0	0	0	0	0	0	0	0	0	0	0	0	0
	J00-J06	0	0	0	0	0	0	0	0	0	0	0	0	0	1	0	0	0	0	0	0	0	0	2
	J09-J18	89	0	2	10	1	11	34	12	37	64	5	0	0	1	5,506	5	0	43	14	7	11	2	13
	J20-J22	14	0	0	1	0	3	2	0	5	9	1	0	0	0	5	546	0	40	1	0	0	0	7
	J30-J39	0	0	0	1	0	0	0	0	0	2	0	0	0	0	0	0	1	0	0	0	0	0	0
	J40-J47	66	0	0	17	0	22	33	9	58	39	0	2	0	2	149	35	1	2,594	16	23	9	0	35
	J60-J70	4	0	0	0	0	1	2	0	1	17	0	0	0	0	11	0	0	1	530	2	0	0	2
	J80-J84	5	0	0	0	0	3	3	5	16	0	0	0	0	0	9	0	1	2	11	349	0	0	5
	J85-J86	2	0	0	0	0	0	0	0	0	0	0	0	0	0	0	0	0	0	0	0	17	0	0
	J90-J94	6	0	0	1	0	0	1	0	8	0	0	0	0	0	5	0	0	0	1	1	0	33	0
	J95-J99	49	0	2	2	0	15	25	5	44	41	7	3	0	9	47	15	0	37	21	22	3	0	1,790

Caption: C00-C97 - Malignant neoplasms; D00-D09 - In situ neoplasms; D10-D36 - Benign neoplasms; D37-D48 - Neoplasms of uncertain or unknown behaviour; I05-I09 - Chronic rheumatic heart diseases; I10-I15 - Hypertensive diseases; I20-I25 - Ischaemic heart diseases; I26-I28 - Pulmonary heart disease and diseases of pulmonary circulation; I30-I52 - Other forms of heart disease; I60-I69 - Cerebrovascular diseases; I70-I79 - Diseases of arteries, arterioles and capillaries; I80-I89 - Diseases of veins, lymphatic vessels and lymph nodes, not elsewhere classified; I95-I99 - Other and unspecified disorders of the circulatory system; J00-J06 - Acute upper respiratory infections; J09-J18 - Influenza and pneumonia; J20-J22 - Other acute lower respiratory infections; J30-J39 - Other diseases of upper respiratory tract; J40-J47 - Chronic lower respiratory diseases; J60-J70 - Lung diseases due to external agents; J80-J84 - Other respiratory diseases principally affecting the interstitium; J85-J86 - Suppurative and necrotic conditions of lower respiratory tract; J90-J94 - Other diseases of pleura; J95-J99 - Other diseases of the respiratory system

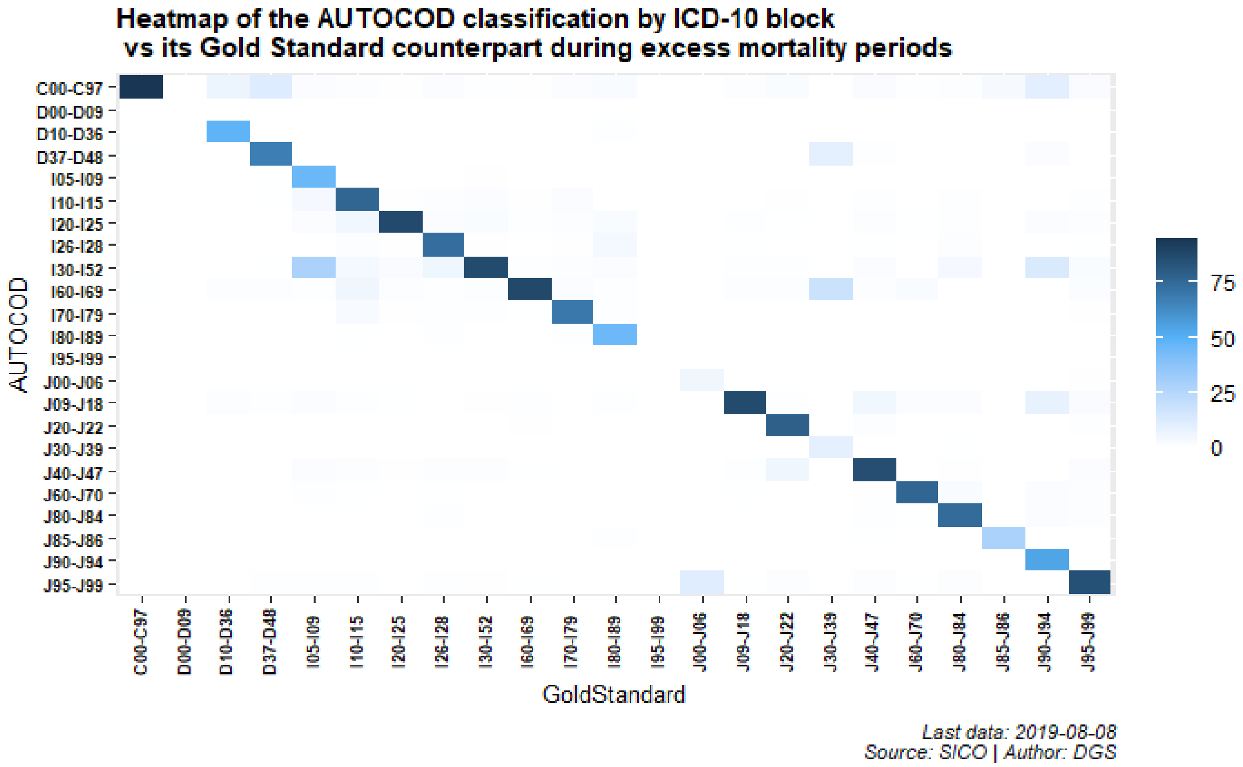

Supplementary Figure 27 - Heatmap of the AUTOCOD classification by ICD-10 block vs its Gold Standard counterpart. Values on the diagonal represent correct classifications, during excess mortality periods

Caption: C00-C97 - Malignant neoplasms; D00-D09 - In situ neoplasms; D10-D36 - Benign neoplasms; D37-D48 - Neoplasms of uncertain or unknown behaviour; I05-I09 - Chronic rheumatic heart diseases; I10-I15 - Hypertensive diseases; I20-I25 - Ischaemic heart diseases; I26-I28 - Pulmonary heart disease and diseases of pulmonary circulation; I30-I52 - Other forms of heart disease; I60-I69 - Cerebrovascular diseases; I70-I79 - Diseases of arteries, arterioles and capillaries; I80-I89 - Diseases of veins, lymphatic vessels and lymph nodes, not elsewhere classified; I95-I99 - Other and unspecified disorders of the circulatory system; J00-J06 - Acute upper respiratory infections; J09-J18 - Influenza and pneumonia; J20-J22 - Other acute lower respiratory infections; J30-J39 - Other diseases of upper respiratory tract; J40-J47 - Chronic lower respiratory diseases; J60-J70 - Lung diseases due to external agents; J80-J84 - Other respiratory diseases principally affecting the interstitium; J85-J86 - Suppurative and necrotic conditions of lower respiratory tract; J90-J94 - Other diseases of pleura; J95-J99 - Other diseases of the respiratory system

Supplementary Table 42 - Overall performance metrics of AUTOCOD when classifying by ICD-10 block, excess mortality periods

**Accuracy**	0.93
**Kappa**	0.91
**AccuracyLower**	0.93
**AccuracyUpper**	0.93
**AccuracyNull**	0.34
**AccuracyPValue**	0.00

Supplementary Table 43 - Performance Metrics of AUTOCOD by ICD-10 block, excess mortality periods

	C00-C97	D00-D09	D10-D36	D37-D48	I05-I09	I10-I15	I20-I25	I26-I28	I30-I52	I60-I69	I70-I79	I80-I89	I95-I99	J00-J06	J09-J18	J20-J22	J30-J39	J40-J47	J60-J70	J80-J84	J85-J86	J90-J94	J95-J99
**Sensitivity**	0.98	NA	0.69	0.73	0.49	0.86	0.92	0.79	0.92	0.94	0.82	0.54	0.00	0.07	0.93	0.83	0.17	0.89	0.84	0.81	0.35	0.69	0.92
**Specificity**	0.99	1.00	1.00	1.00	1.00	0.99	0.99	1.00	0.99	0.99	1.00	1.00	1.00	1.00	0.99	1.00	1.00	0.99	1.00	1.00	1.00	1.00	0.99
**Pos Pred Value**	0.99	NA	0.84	0.82	0.53	0.76	0.93	0.82	0.90	0.95	0.88	0.76	NaN	0.33	0.94	0.86	0.25	0.83	0.93	0.85	0.90	0.59	0.84
**Neg Pred Value**	0.99	NA	1.00	1.00	1.00	1.00	0.99	1.00	0.99	0.99	1.00	1.00	1.00	1.00	0.99	1.00	1.00	1.00	1.00	1.00	1.00	1.00	1.00
**F1**	0.98	NA	0.76	0.77	0.51	0.81	0.93	0.80	0.91	0.95	0.85	0.63	NA	0.12	0.94	0.85	0.20	0.86	0.88	0.83	0.51	0.64	0.88
**Prevalence**	0.34	0.00	0.00	0.01	0.00	0.04	0.10	0.01	0.12	0.15	0.02	0.00	0.00	0.00	0.10	0.01	0.00	0.05	0.01	0.01	0.00	0.00	0.03
**Detection Rate**	0.33	0.00	0.00	0.00	0.00	0.03	0.09	0.01	0.11	0.14	0.02	0.00	0.00	0.00	0.09	0.01	0.00	0.04	0.01	0.01	0.00	0.00	0.03
**Detection Prevalence**	0.33	0.00	0.00	0.01	0.00	0.04	0.10	0.01	0.13	0.15	0.02	0.00	0.00	0.00	0.10	0.01	0.00	0.05	0.01	0.01	0.00	0.00	0.04
**Balanced Accuracy**	0.99	NA	0.84	0.86	0.75	0.92	0.96	0.89	0.95	0.97	0.91	0.77	0.50	0.54	0.96	0.92	0.58	0.94	0.92	0.91	0.68	0.84	0.96

Caption: C00-C97 - Malignant neoplasms; D00-D09 - In situ neoplasms; D10-D36 - Benign neoplasms; D37-D48 - Neoplasms of uncertain or unknown behaviour; I05-I09 - Chronic rheumatic heart diseases; I10-I15 - Hypertensive diseases; I20-I25 - Ischaemic heart diseases; I26-I28 - Pulmonary heart disease and diseases of pulmonary circulation; I30-I52 - Other forms of heart disease; I60-I69 - Cerebrovascular diseases; I70-I79 - Diseases of arteries, arterioles and capillaries; I80-I89 - Diseases of veins, lymphatic vessels and lymph nodes, not elsewhere classified; I95-I99 - Other and unspecified disorders of the circulatory system; J00-J06 - Acute upper respiratory infections; J09-J18 - Influenza and pneumonia; J20-J22 - Other acute lower respiratory infections; J30-J39 - Other diseases of upper respiratory tract; J40-J47 - Chronic lower respiratory diseases; J60-J70 - Lung diseases due to external agents; J80-J84 - Other respiratory diseases principally affecting the interstitium; J85-J86 - Suppurative and necrotic conditions of lower respiratory tract; J90-J94 - Other diseases of pleura; J95-J99 - Other diseases of the respiratory system

Periods without excess mortality

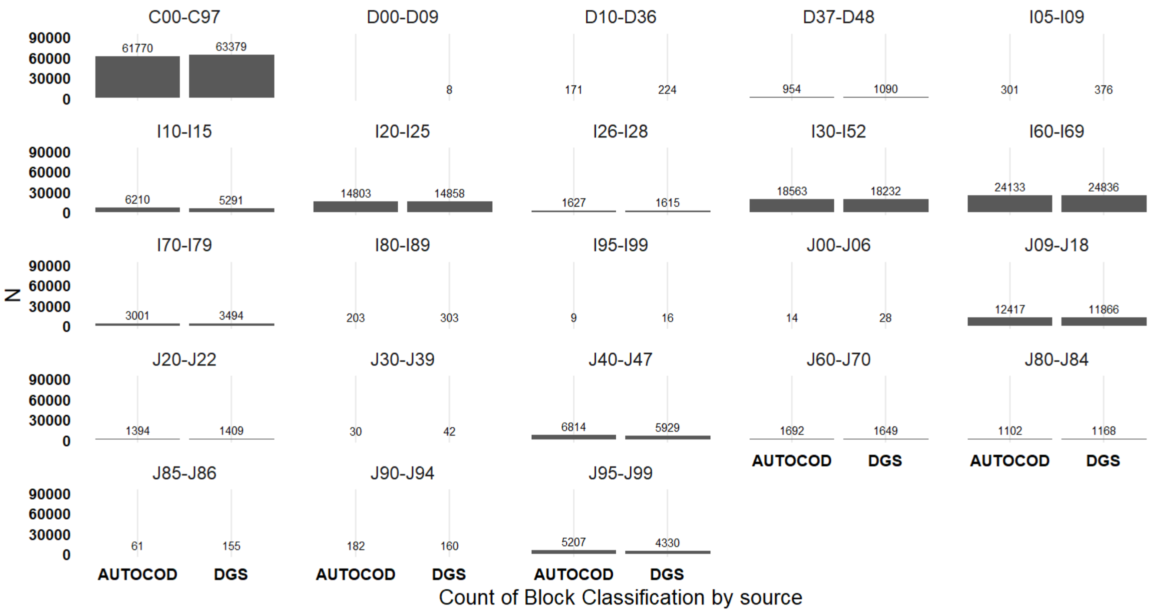

Supplementary Figure 28 - Count of death certificates classified by ICD-10 block either by human coders (DGS) or by AUTOCOD, in periods without excess mortality

Supplementary Table 44 - Confusion matrix for ICD-10 block classification, periods without excess mortality

		Human Coders (GOLD-STANDARD)																						
		C00-C97	D00-D09	D10-D36	D37-D48	I05-I09	I10-I15	I20-I25	I26-I28	I30-I52	I60-I69	I70-I79	I80-I89	I95-I99	J00-J06	J09-J18	J20-J22	J30-J39	J40-J47	J60-J70	J80-J84	J85-J86	J90-J94	J95-J99
AUTOCOD	C00-C97	#####	3	20	128	3	32	40	10	75	71	14	11	0	1	37	10	0	29	11	11	1	3	13
	D00-D09	0	0	0	0	0	0	0	0	0	0	0	0	0	0	0	0	0	0	0	0	0	0	0
	D10-D36	17	0	120	3	0	0	0	0	1	7	0	0	0	0	8	0	0	1	0	1	0	0	1
	D37-D48	104	0	0	716	1	2	2	0	4	7	1	0	0	0	8	0	1	9	0	4	0	1	5
	I05-I09	3	0	0	0	139	5	13	0	103	7	2	1	0	0	0	0	0	0	0	0	0	0	0
	I10-I15	70	0	4	7	8	4,055	251	56	213	335	157	7	0	0	37	0	0	61	22	20	0	3	36
	I20-I25	77	1	4	7	14	66	13,168	50	360	135	59	13	1	0	25	6	0	44	2	8	0	4	25
	I26-I28	38	0	1	5	7	20	24	1,205	107	10	16	11	0	0	8	0	0	18	0	9	0	4	6
	I30-I52	149	0	0	10	157	329	371	82	15,638	209	194	11	3	0	52	11	0	108	8	20	8	12	53
	I60-I69	144	0	13	23	7	95	121	20	323	21,916	65	11	1	1	125	93	4	79	30	10	6	2	65
	I70-I79	47	0	0	2	0	52	40	10	42	55	2,455	20	4	0	8	1	0	2	0	0	2	0	6
	I80-I89	11	0	0	0	0	2	0	4	6	9	4	121	1	0	1	0	0	0	0	0	0	2	1
	I95-I99	0	0	0	0	0	0	0	1	0	1	2	0	1	0	0	0	0	0	0	0	0	0	0
	J00-J06	1	0	0	0	0	0	0	0	0	1	0	0	0	7	0	0	0	0	0	0	0	0	1
	J09-J18	200	0	4	20	2	27	67	20	71	151	20	1	1	1	10,556	6	1	91	26	23	32	7	36
	J20-J22	18	0	0	0	1	0	1	0	7	43	4	0	0	1	7	1,114	0	63	1	1	1	0	14
	J30-J39	0	0	0	0	1	0	0	0	1	3	0	0	0	0	0	0	9	3	0	0	0	0	0
	J40-J47	167	0	1	35	0	36	47	27	119	147	7	2	0	1	223	64	2	5,039	48	48	33	5	59
	J60-J70	23	1	0	3	0	6	0	1	9	36	3	0	0	0	25	0	2	7	1,312	3	0	1	4
	J80-J84	21	0	1	1	0	5	13	5	17	1	0	2	0	0	15	4	0	12	14	896	1	0	4
	J85-J86	3	0	0	0	0	0	0	2	2	0	0	0	0	0	1	0	0	0	0	1	43	0	0
	J90-J94	15	0	0	2	0	1	0	0	17	3	0	1	0	0	7	5	0	0	3	0	0	93	0
	J95-J99	115	0	3	3	0	24	31	16	95	93	7	7	1	5	72	27	1	91	34	32	10	3	3,718

Caption: C00-C97 - Malignant neoplasms; D00-D09 - In situ neoplasms; D10-D36 - Benign neoplasms; D37-D48 - Neoplasms of uncertain or unknown behaviour; I05-I09 - Chronic rheumatic heart diseases; I10-I15 - Hypertensive diseases; I20-I25 - Ischaemic heart diseases; I26-I28 - Pulmonary heart disease and diseases of pulmonary circulation; I30-I52 - Other forms of heart disease; I60-I69 - Cerebrovascular diseases; I70-I79 - Diseases of arteries, arterioles and capillaries; I80-I89 - Diseases of veins, lymphatic vessels and lymph nodes, not elsewhere classified; I95-I99 - Other and unspecified disorders of the circulatory system; J00-J06 - Acute upper respiratory infections; J09-J18 - Influenza and pneumonia; J20-J22 - Other acute lower respiratory infections; J30-J39 - Other diseases of upper respiratory tract; J40-J47 - Chronic lower respiratory diseases; J60-J70 - Lung diseases due to external agents; J80-J84 - Other respiratory diseases principally affecting the interstitium; J85-J86 - Suppurative and necrotic conditions of lower respiratory tract; J90-J94 - Other diseases of pleura; J95-J99 - Other diseases of the respiratory system

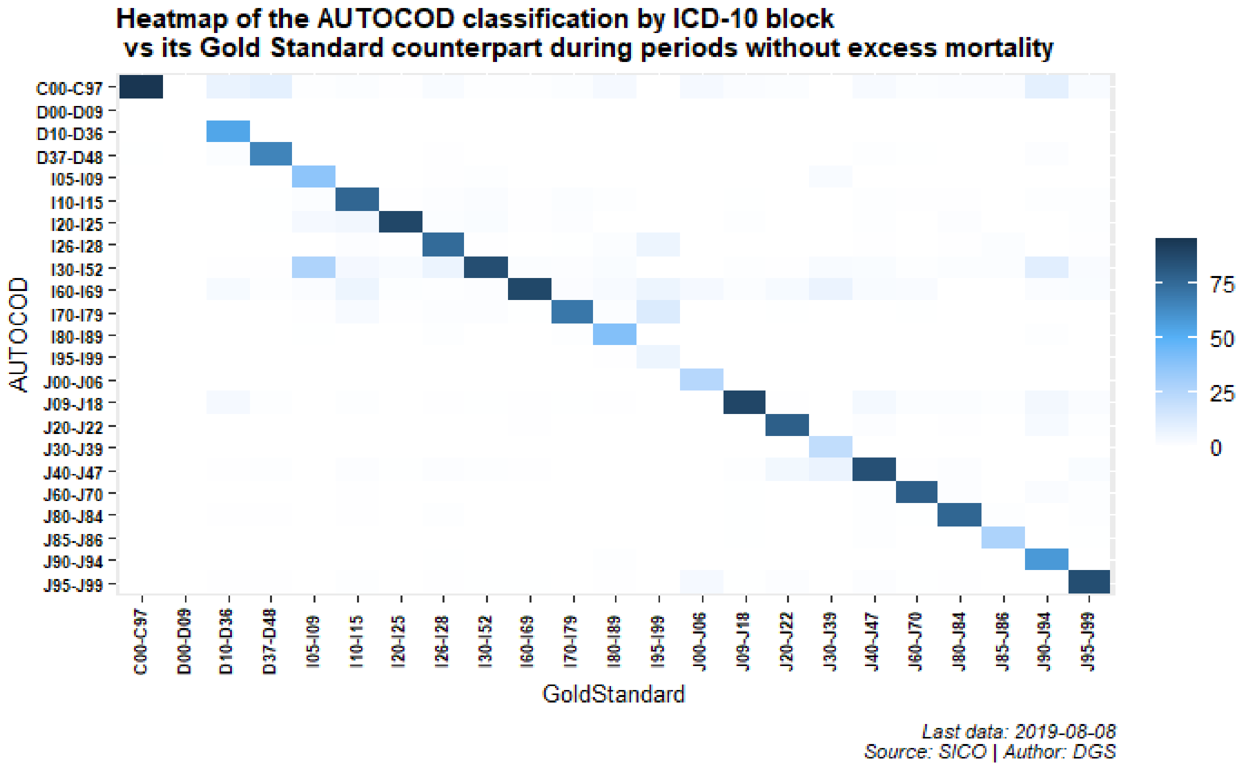

Supplementary Figure 29 - Heatmap of the AUTOCOD classification by ICD-10 block vs its Gold Standard counterpart. Values on the diagonal represent correct classifications, during periods without excess mortality

Caption: C00-C97 - Malignant neoplasms; D00-D09 - In situ neoplasms; D10-D36 - Benign neoplasms; D37-D48 - Neoplasms of uncertain or unknown behaviour; I05-I09 - Chronic rheumatic heart diseases; I10-I15 - Hypertensive diseases; I20-I25 - Ischaemic heart diseases; I26-I28 - Pulmonary heart disease and diseases of pulmonary circulation; I30-I52 - Other forms of heart disease; I60-I69 - Cerebrovascular diseases; I70-I79 - Diseases of arteries, arterioles and capillaries; I80-I89 - Diseases of veins, lymphatic vessels and lymph nodes, not elsewhere classified; I95-I99 - Other and unspecified disorders of the circulatory system; J00-J06 - Acute upper respiratory infections; J09-J18 - Influenza and pneumonia; J20-J22 - Other acute lower respiratory infections; J30-J39 - Other diseases of upper respiratory tract; J40-J47 - Chronic lower respiratory diseases; J60-J70 - Lung diseases due to external agents; J80-J84 - Other respiratory diseases principally affecting the interstitium; J85-J86 - Suppurative and necrotic conditions of lower respiratory tract; J90-J94 - Other diseases of pleura; J95-J99 - Other diseases of the respiratory system

Supplementary Table 45 - Overall performance metrics of AUTOCOD when classifying by ICD-10 block, periods without excess mortality

**Accuracy**	0.94
**Kappa**	0.92
**AccuracyLower**	0.94
**AccuracyUpper**	0.94
**AccuracyNull**	0.40
**AccuracyPValue**	0.00

Supplementary Table 46 - Performance Metrics of AUTOCOD by ICD-10 block, periods without excess mortality

	C00-C97	D00-D09	D10-D36	D37-D48	I05-I09	I10-I15	I20-I25	I26-I28	I30-I52	I60-I69	I70-I79	I80-I89	I95-I99	J00-J06	J09-J18	J20-J22	J30-J39	J40-J47	J60-J70	J80-J84	J85-J86	J90-J94	J95-J99
**Sensitivity**	0.98	0.00	0.70	0.74	0.41	0.85	0.93	0.80	0.91	0.94	0.82	0.55	0.08	0.41	0.94	0.83	0.45	0.89	0.87	0.82	0.31	0.66	0.92
**Specificity**	0.99	1.00	1.00	1.00	1.00	0.99	0.99	1.00	0.99	0.99	1.00	1.00	1.00	1.00	0.99	1.00	1.00	0.99	1.00	1.00	1.00	1.00	1.00
**Pos Pred Value**	0.99	NaN	0.76	0.83	0.51	0.76	0.94	0.81	0.90	0.95	0.89	0.75	0.20	0.70	0.93	0.87	0.53	0.83	0.91	0.89	0.83	0.63	0.85
**Neg Pred Value**	0.99	1.00	1.00	1.00	1.00	1.00	0.99	1.00	0.99	0.99	1.00	1.00	1.00	1.00	1.00	1.00	1.00	1.00	1.00	1.00	1.00	1.00	1.00
**F1**	0.99	NA	0.73	0.78	0.45	0.80	0.93	0.80	0.90	0.95	0.85	0.64	0.11	0.52	0.94	0.85	0.49	0.86	0.89	0.85	0.46	0.65	0.88
**Prevalence**	0.40	0.00	0.00	0.01	0.00	0.03	0.09	0.01	0.11	0.15	0.02	0.00	0.00	0.00	0.07	0.01	0.00	0.04	0.01	0.01	0.00	0.00	0.03
**Detection Rate**	0.40	0.00	0.00	0.01	0.00	0.03	0.09	0.01	0.10	0.14	0.02	0.00	0.00	0.00	0.07	0.01	0.00	0.03	0.01	0.01	0.00	0.00	0.02
**Detection Prevalence**	0.40	0.00	0.00	0.01	0.00	0.04	0.09	0.01	0.11	0.15	0.02	0.00	0.00	0.00	0.08	0.01	0.00	0.04	0.01	0.01	0.00	0.00	0.03
**Balanced Accuracy**	0.99	0.50	0.85	0.87	0.70	0.92	0.96	0.90	0.95	0.97	0.91	0.78	0.54	0.71	0.97	0.92	0.73	0.94	0.93	0.91	0.66	0.83	0.96

Periods with severe excess mortality (>4 SDs)

Supplementary Table 47 - Confusion matrix for ICD-10 block classification, severe excess mortality periods (>4 SDs)

		Human Coders (GOLD-STANDARD)																						
		**C00-C97**	**D00-D09**	**D10-D36**	**D37-D48**	**I05-I09**	**I10-I15**	**I20-I25**	**I26-I28**	**I30-I52**	**I60-I69**	**I70-I79**	**I80-I89**	**I95-I99**	**J00-J06**	**J09-J18**	**J20-J22**	**J30-J39**	**J40-J47**	**J60-J70**	**J80-J84**	**J85-J86**	**J90-J94**	**J95-J99**
**AUTOCOD**	**C00-C97**	5,614	0	3	13	0	4	11	1	14	16	3	2	0	0	6	1	0	1	1	1	0	1	3
	**D00-D09**	0	0	0	0	0	0	0	0	0	0	0	0	0	0	0	0	0	0	0	0	0	0	0
	**D10-D36**	0	0	13	0	0	0	0	0	0	1	0	0	0	0	0	0	0	0	0	0	0	0	0
	**D37-D48**	19	0	0	84	1	1	0	0	0	2	0	0	0	0	0	0	0	0	0	0	0	0	3
	**I05-I09**	1	0	0	0	24	3	0	0	12	1	0	0	0	0	0	0	0	1	0	0	0	0	0
	**I10-I15**	8	0	0	1	1	617	35	8	30	45	21	2	0	0	7	0	0	11	3	2	0	0	5
	**I20-I25**	10	0	0	0	0	6	1,831	9	30	18	5	0	1	0	5	1	1	6	1	0	1	0	0
	**I26-I28**	5	0	0	0	0	4	5	157	10	2	2	1	0	0	0	0	0	5	0	3	0	1	2
	**I30-I52**	24	0	0	2	12	41	50	15	2,172	38	17	4	0	0	11	4	0	28	1	5	0	2	7
	**I60-I69**	11	0	0	3	0	10	19	1	23	2,767	6	0	0	0	23	10	1	5	5	2	0	0	7
	**I70-I79**	11	0	0	0	0	4	4	2	5	10	308	4	2	0	0	0	0	0	0	0	0	0	1
	**I80-I89**	1	0	0	0	0	0	0	2	1	0	0	19	0	0	0	0	0	0	0	0	0	0	0
	**I95-I99**	0	0	0	0	0	0	0	0	0	0	0	0	0	0	0	0	0	0	0	0	0	0	0
	**J00-J06**	0	0	0	0	0	0	0	0	0	0	0	0	0	0	0	0	0	0	0	0	0	0	1
	**J09-J18**	28	0	1	3	0	6	13	2	11	24	1	0	0	1	1,954	1	0	19	4	1	8	1	4
	**J20-J22**	1	0	0	0	0	1	0	0	2	3	0	0	0	0	2	202	0	13	0	0	0	0	4
	**J30-J39**	0	0	0	0	0	0	0	0	0	1	0	0	0	0	0	0	1	0	0	0	0	0	0
	**J40-J47**	18	0	0	7	0	8	12	2	21	17	0	0	0	2	56	14	1	924	8	7	2	0	14
	**J60-J70**	0	0	0	0	0	1	0	0	1	9	0	0	0	0	4	0	0	0	164	0	0	0	1
	**J80-J84**	2	0	0	0	0	0	1	1	5	0	0	0	0	0	6	0	0	1	4	109	0	0	2
	**J85-J86**	1	0	0	0	0	0	0	0	0	0	0	0	0	0	0	0	0	0	0	0	1	0	0
	**J90-J94**	0	0	0	0	0	0	0	0	2	0	0	0	0	0	3	0	0	0	0	0	0	12	0
	**J95-J99**	14	0	1	0	0	4	10	4	12	12	1	1	0	3	23	6	0	12	4	8	0	0	630

Caption: C00-C97 - Malignant neoplasms; D00-D09 - In situ neoplasms; D10-D36 - Benign neoplasms; D37-D48 - Neoplasms of uncertain or unknown behaviour; I05-I09 - Chronic rheumatic heart diseases; I10-I15 - Hypertensive diseases; I20-I25 - Ischaemic heart diseases; I26-I28 - Pulmonary heart disease and diseases of pulmonary circulation; I30-I52 - Other forms of heart disease; I60-I69 - Cerebrovascular diseases; I70-I79 - Diseases of arteries, arterioles and capillaries; I80-I89 - Diseases of veins, lymphatic vessels and lymph nodes, not elsewhere classified; I95-I99 - Other and unspecified disorders of the circulatory system; J00-J06 - Acute upper respiratory infections; J09-J18 - Influenza and pneumonia; J20-J22 - Other acute lower respiratory infections; J30-J39 - Other diseases of upper respiratory tract; J40-J47 - Chronic lower respiratory diseases; J60-J70 - Lung diseases due to external agents; J80-J84 - Other respiratory diseases principally affecting the interstitium; J85-J86 - Suppurative and necrotic conditions of lower respiratory tract; J90-J94 - Other diseases of pleura; J95-J99 - Other diseases of the respiratory system

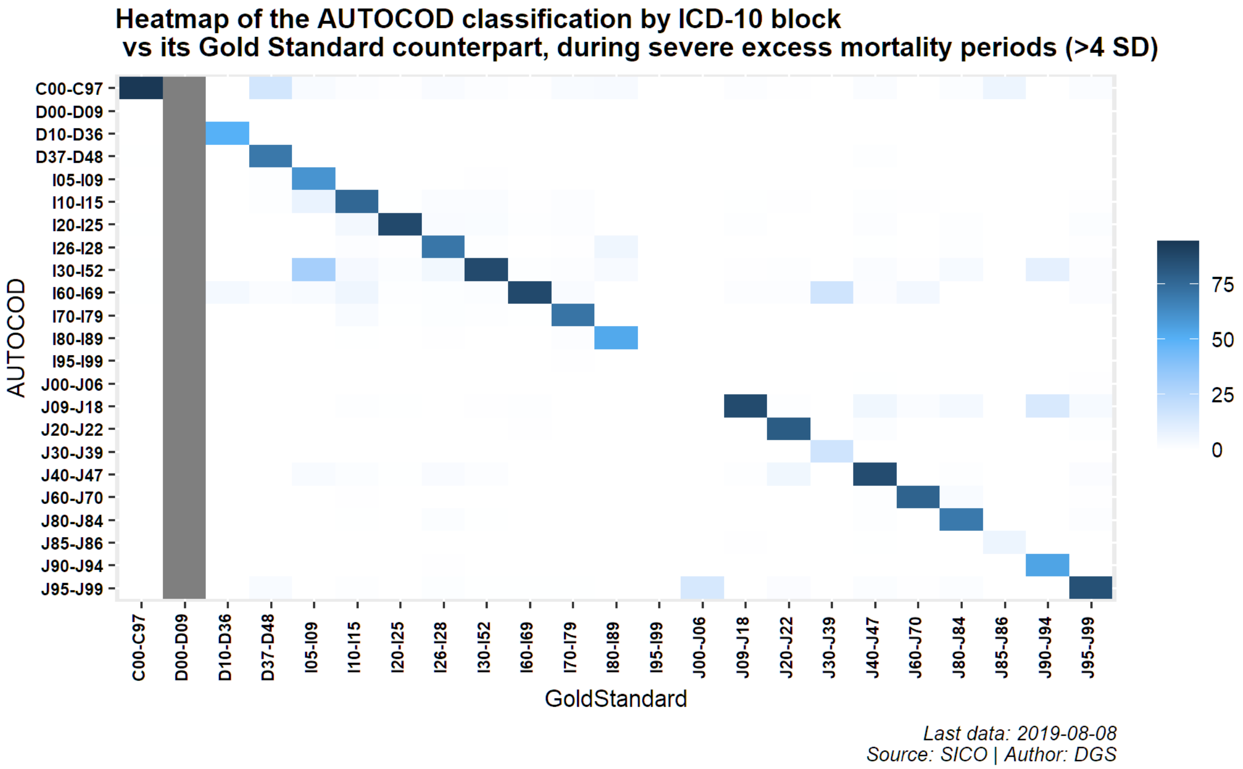

Supplementary Figure 30 - Heatmap of the AUTOCOD classification by ICD-10 block vs its Gold Standard counterpart. Values on the diagonal represent correct classifications

Supplementary Table 48 - Overall performance metrics of AUTOCOD when classifying by ICD-10 block, severe excess mortality periods (>4 SDs)

**Accuracy**	0.93
**Kappa**	0.91
**AccuracyLower**	0.92
**AccuracyUpper**	0.93
**AccuracyNull**	0.30
**AccuracyPValue**	0.00

Supplementary Table 49 - Performance Metrics of AUTOCOD by ICD-10 block, severe excess mortality periods (>4 SDs)

	C00-C97	D00-D09	D10-D36	D37-D48	I05-I09	I10-I15	I20-I25	I26-I28	I30-I52	I60-I69	I70-I79	I80-I89	I95-I99	J00-J06	J09-J18	J20-J22	J30-J39	J40-J47	J60-J70	J80-J84	J85-J86	J90-J94	J95-J99
**Sensitivity**	0.97	NA	0.72	0.74	0.63	0.87	0.92	0.77	0.92	0.93	0.85	0.58	0.00	0.00	0.93	0.85	0.25	0.90	0.84	0.79	0.08	0.71	0.92
**Specificity**	0.99	1.00	1.00	1.00	1.00	0.99	0.99	1.00	0.98	0.99	1.00	1.00	1.00	1.00	0.99	1.00	1.00	0.99	1.00	1.00	1.00	1.00	0.99
**Pos Pred Value**	0.99	NA	0.93	0.76	0.57	0.78	0.95	0.80	0.89	0.96	0.88	0.83	NA	0.00	0.94	0.89	0.50	0.83	0.91	0.83	0.50	0.71	0.85
**Neg Pred Value**	0.99	NA	1.00	1.00	1.00	1.00	0.99	1.00	0.99	0.99	1.00	1.00	1.00	1.00	0.99	1.00	1.00	0.99	1.00	1.00	1.00	1.00	1.00
**F1**	0.98	NA	0.81	0.75	0.60	0.82	0.94	0.78	0.91	0.95	0.86	0.68	NA	NA	0.93	0.87	0.33	0.86	0.88	0.81	0.14	0.71	0.88
**Prevalence**	0.30	0.00	0.00	0.01	0.00	0.04	0.11	0.01	0.12	0.16	0.02	0.00	0.00	0.00	0.11	0.01	0.00	0.05	0.01	0.01	0.00	0.00	0.04
**Detection Rate**	0.30	0.00	0.00	0.00	0.00	0.03	0.10	0.01	0.11	0.15	0.02	0.00	0.00	0.00	0.10	0.01	0.00	0.05	0.01	0.01	0.00	0.00	0.03
**Detection Prevalence**	0.30	0.00	0.00	0.01	0.00	0.04	0.10	0.01	0.13	0.15	0.02	0.00	0.00	0.00	0.11	0.01	0.00	0.06	0.01	0.01	0.00	0.00	0.04
**Balanced Accuracy**	0.98	NA	0.86	0.87	0.82	0.93	0.96	0.88	0.95	0.96	0.92	0.79	0.50	0.50	0.96	0.92	0.63	0.95	0.92	0.89	0.54	0.85	0.96

Caption: C00-C97 - Malignant neoplasms; D00-D09 - In situ neoplasms; D10-D36 - Benign neoplasms; D37-D48 - Neoplasms of uncertain or unknown behaviour; I05-I09 - Chronic rheumatic heart diseases; I10-I15 - Hypertensive diseases; I20-I25 - Ischaemic heart diseases; I26-I28 - Pulmonary heart disease and diseases of pulmonary circulation; I30-I52 - Other forms of heart disease; I60-I69 - Cerebrovascular diseases; I70-I79 - Diseases of arteries, arterioles and capillaries; I80-I89 - Diseases of veins, lymphatic vessels and lymph nodes, not elsewhere classified; I95-I99 - Other and unspecified disorders of the circulatory system; J00-J06 - Acute upper respiratory infections; J09-J18 - Influenza and pneumonia; J20-J22 - Other acute lower respiratory infections; J30-J39 - Other diseases of upper respiratory tract; J40-J47 - Chronic lower respiratory diseases; J60-J70 - Lung diseases due to external agents; J80-J84 - Other respiratory diseases principally affecting the interstitium; J85-J86 - Suppurative and necrotic conditions of lower respiratory tract; J90-J94 - Other diseases of pleura; J95-J99 - Other diseases of the respiratory system

Periods with extreme excess mortality (>6 SDs)

Supplementary Table 50 - Confusion matrix for ICD-10 block classification, extreme excess mortality periods (>6 SDs)

		**Human Coders (GOLD-STANDARD)**																						
		**C00-C97**	**D00-D09**	**D10-D36**	**D37-D48**	**I05-I09**	**I10-I15**	**I20-I25**	**I26-I28**	**I30-I52**	**I60-I69**	**I70-I79**	**I80-I89**	**I95-I99**	**J00-J06**	**J09-J18**	**J20-J22**	**J30-J39**	**J40-J47**	**J60-J70**	**J80-J84**	**J85-J86**	**J90-J94**	**J95-J99**
	**C00-C97**	1,085	0	0	1	0	1	1	0	4	4	0	0	0	0	2	0	0	0	1	1	0	1	1
	**D00-D09**	0	0	0	0	0	0	0	0	0	0	0	0	0	0	0	0	0	0	0	0	0	0	0
	**D10-D36**	0	0	4	0	0	0	0	0	0	0	0	0	0	0	0	0	0	0	0	0	0	0	0
	**D37-D48**	9	0	0	13	0	0	0	0	0	1	0	0	0	0	0	0	0	0	0	0	0	0	1
	**I05-I09**	0	0	0	0	2	0	0	0	2	0	0	0	0	0	0	0	0	0	0	0	0	0	0
	**I10-I15**	2	0	0	1	0	116	6	1	7	8	3	0	0	0	4	0	0	0	1	0	0	0	0
	**I20-I25**	3	0	0	0	0	3	412	3	9	5	1	0	0	0	0	1	0	4	0	0	0	0	0
	**I26-I28**	2	0	0	0	0	1	2	32	2	1	1	0	0	0	0	0	0	0	0	0	0	0	0
	**I30-I52**	2	0	0	0	1	6	9	4	458	8	7	1	0	0	3	0	0	5	0	0	0	1	4
	**I60-I69**	1	0	0	0	0	2	8	0	5	544	1	0	0	0	2	1	0	3	0	0	0	0	1
	**I70-I79**	2	0	0	0	0	1	1	0	1	2	72	0	0	0	0	0	0	0	0	0	0	0	0
**AUTOCOD**	**I80-I89**	0	0	0	0	0	0	0	0	0	0	0	2	0	0	0	0	0	0	0	0	0	0	0
	**I95-I99**	0	0	0	0	0	0	0	0	0	0	0	0	0	0	0	0	0	0	0	0	0	0	0
	**J00-J06**	0	0	0	0	0	0	0	0	0	0	0	0	0	0	0	0	0	0	0	0	0	0	0
	**J09-J18**	6	0	0	1	0	0	2	0	2	7	0	0	0	0	419	0	0	3	0	0	0	0	1
	**J20-J22**	0	0	0	0	0	0	0	0	1	1	0	0	0	0	0	51	0	2	0	0	0	0	0
	**J30-J39**	0	0	0	0	0	0	0	0	0	0	0	0	0	0	0	0	0	0	0	0	0	0	0
	**J40-J47**	4	0	0	1	0	2	5	0	6	2	0	0	0	0	11	3	0	197	3	0	1	0	5
	**J60-J70**	0	0	0	0	0	0	0	0	0	2	0	0	0	0	0	0	0	0	31	0	0	0	0
	**J80-J84**	1	0	0	0	0	0	0	0	0	0	0	0	0	0	4	0	0	0	1	21	0	0	0
	**J85-J86**	0	0	0	0	0	0	0	0	0	0	0	0	0	0	0	0	0	0	0	0	0	0	0
	**J90-J94**	0	0	0	0	0	0	0	0	1	0	0	0	0	0	0	0	0	0	0	0	0	2	0
	**J95-J99**	0	0	1	0	0	1	1	0	2	3	1	1	0	1	5	1	0	7	1	2	0	0	133

Caption: C00-C97 - Malignant neoplasms; D00-D09 - In situ neoplasms; D10-D36 - Benign neoplasms; D37-D48 - Neoplasms of uncertain or unknown behaviour; I05-I09 - Chronic rheumatic heart diseases; I10-I15 - Hypertensive diseases; I20-I25 - Ischaemic heart diseases; I26-I28 - Pulmonary heart disease and diseases of pulmonary circulation; I30-I52 - Other forms of heart disease; I60-I69 - Cerebrovascular diseases; I70-I79 - Diseases of arteries, arterioles and capillaries; I80-I89 - Diseases of veins, lymphatic vessels and lymph nodes, not elsewhere classified; I95-I99 - Other and unspecified disorders of the circulatory system; J00-J06 - Acute upper respiratory infections; J09-J18 - Influenza and pneumonia; J20-J22 - Other acute lower respiratory infections; J30-J39 - Other diseases of upper respiratory tract; J40-J47 - Chronic lower respiratory diseases; J60-J70 - Lung diseases due to external agents; J80-J84 - Other respiratory diseases principally affecting the interstitium; J85-J86 - Suppurative and necrotic conditions of lower respiratory tract; J90-J94 - Other diseases of pleura; J95-J99 - Other diseases of the respiratory system

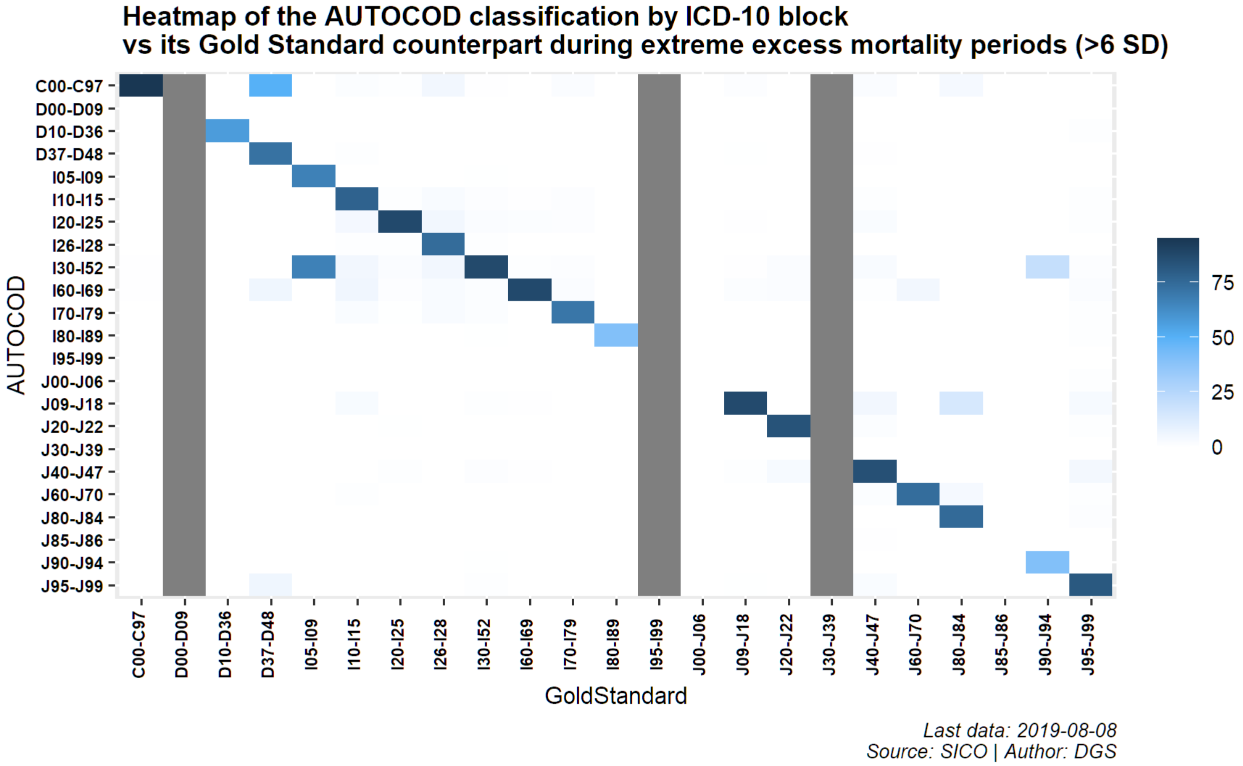

Supplementary Figure 31 - Heatmap of the AUTOCOD classification by ICD-10 block vs its Gold Standard counterpart. Values on the diagonal represent correct classifications, during extreme excess mortality periods (>6 SDs)

Caption: C00-C97 - Malignant neoplasms; D00-D09 - In situ neoplasms; D10-D36 - Benign neoplasms; D37-D48 - Neoplasms of uncertain or unknown behaviour; I05-I09 - Chronic rheumatic heart diseases; I10-I15 - Hypertensive diseases; I20-I25 - Ischaemic heart diseases; I26-I28 - Pulmonary heart disease and diseases of pulmonary circulation; I30-I52 - Other forms of heart disease; I60-I69 - Cerebrovascular diseases; I70-I79 - Diseases of arteries, arterioles and capillaries; I80-I89 - Diseases of veins, lymphatic vessels and lymph nodes, not elsewhere classified; I95-I99 - Other and unspecified disorders of the circulatory system; J00-J06 - Acute upper respiratory infections; J09-J18 - Influenza and pneumonia; J20-J22 - Other acute lower respiratory infections; J30-J39 - Other diseases of upper respiratory tract; J40-J47 - Chronic lower respiratory diseases; J60-J70 - Lung diseases due to external agents; J80-J84 - Other respiratory diseases principally affecting the interstitium; J85-J86 - Suppurative and necrotic conditions of lower respiratory tract; J90-J94 - Other diseases of pleura; J95-J99 - Other diseases of the respiratory system

Supplementary Table 51 - - Overall performance metrics of AUTOCOD when classifying by ICD-10 block, extreme excess mortality periods (>6 SDs)

**Accuracy**	0.93
**Kappa**	0.91
**AccuracyLower**	0.92
**AccuracyUpper**	0.93
**AccuracyNull**	0.29
**AccuracyPValue**	0.00

Supplementary Table 52 - Performance Metrics of AUTOCOD by ICD-10 block, extreme excess mortality periods (>6 SDs)

	Chapter II - Neoplasms				Chapter IX - Diseases of the circulatory system									Chapter X - Diseases of the respiratory system									
	C00-C97	D00-D09	D10-D36	D37-D48	I05-I09	I10-I15	I20-I25	I26-I28	I30-I52	I60-I69	I70-I79	I80-I89	I95-I99	J00-J06	J09-J18	J20-J22	J30-J39	J40-J47	J60-J70	J80-J84	J85-J86	J90-J94	J95-J99
**Sensitivity**	0.97	NA	0.80	0.77	0.67	0.87	0.92	0.80	0.92	0.93	0.84	0.50	NA	0.00	0.93	0.90	NA	0.89	0.82	0.88	0.00	0.50	0.91
**Specificity**	0.99	1.00	1.00	1.00	1.00	0.99	0.99	1.00	0.99	0.99	1.00	1.00	1.00	1.00	0.99	1.00	1.00	0.99	1.00	1.00	1.00	1.00	0.99
**Pos Pred Value**	0.99	NA	1.00	0.54	0.50	0.78	0.93	0.78	0.90	0.96	0.91	1.00	NA	NA	0.95	0.93	NA	0.82	0.94	0.78	NA	0.67	0.83
**Neg Pred Value**	0.99	NA	1.00	1.00	1.00	1.00	0.99	1.00	0.99	0.99	1.00	1.00	NA	1.00	0.99	1.00	NA	0.99	1.00	1.00	1.00	1.00	1.00
**F1**	0.98	NA	0.89	0.63	0.57	0.82	0.93	0.79	0.91	0.94	0.87	0.67	NA	NA	0.94	0.91	NA	0.86	0.87	0.82	NA	0.57	0.87
**Prevalence**	0.29	0.00	0.00	0.00	0.00	0.03	0.12	0.01	0.13	0.15	0.02	0.00	0.00	0.00	0.12	0.02	0.00	0.06	0.01	0.01	0.00	0.00	0.04
**Detection Rate**	0.28	0.00	0.00	0.00	0.00	0.03	0.11	0.01	0.12	0.14	0.02	0.00	0.00	0.00	0.11	0.01	0.00	0.05	0.01	0.01	0.00	0.00	0.03
**Detection Prevalence**	0.28	0.00	0.00	0.01	0.00	0.04	0.11	0.01	0.13	0.15	0.02	0.00	0.00	0.00	0.11	0.01	0.00	0.06	0.01	0.01	0.00	0.00	0.04
**Balanced Accuracy**	0.98	NA	0.90	0.88	0.83	0.93	0.96	0.90	0.95	0.96	0.92	0.75	NA	0.50	0.96	0.95	NA	0.94	0.91	0.94	0.50	0.75	0.95

Caption: C00-C97 - Malignant neoplasms; D00-D09 - In situ neoplasms; D10-D36 - Benign neoplasms; D37-D48 - Neoplasms of uncertain or unknown behaviour; I05-I09 - Chronic rheumatic heart diseases; I10-I15 - Hypertensive diseases; I20-I25 - Ischaemic heart diseases; I26-I28 - Pulmonary heart disease and diseases of pulmonary circulation; I30-I52 - Other forms of heart disease; I60-I69 - Cerebrovascular diseases; I70-I79 - Diseases of arteries, arterioles and capillaries; I80-I89 - Diseases of veins, lymphatic vessels and lymph nodes, not elsewhere classified; I95-I99 - Other and unspecified disorders of the circulatory system; J00-J06 - Acute upper respiratory infections; J09-J18 - Influenza and pneumonia; J20-J22 - Other acute lower respiratory infections; J30-J39 - Other diseases of upper respiratory tract; J40-J47 - Chronic lower respiratory diseases; J60-J70 - Lung diseases due to external agents; J80-J84 - Other respiratory diseases principally affecting the interstitium; J85-J86 - Suppurative and necrotic conditions of lower respiratory tract; J90-J94 - Other diseases of pleura; J95-J99 - Other diseases of the respiratory system

Difference between excess mortality periods and periods without excess mortality

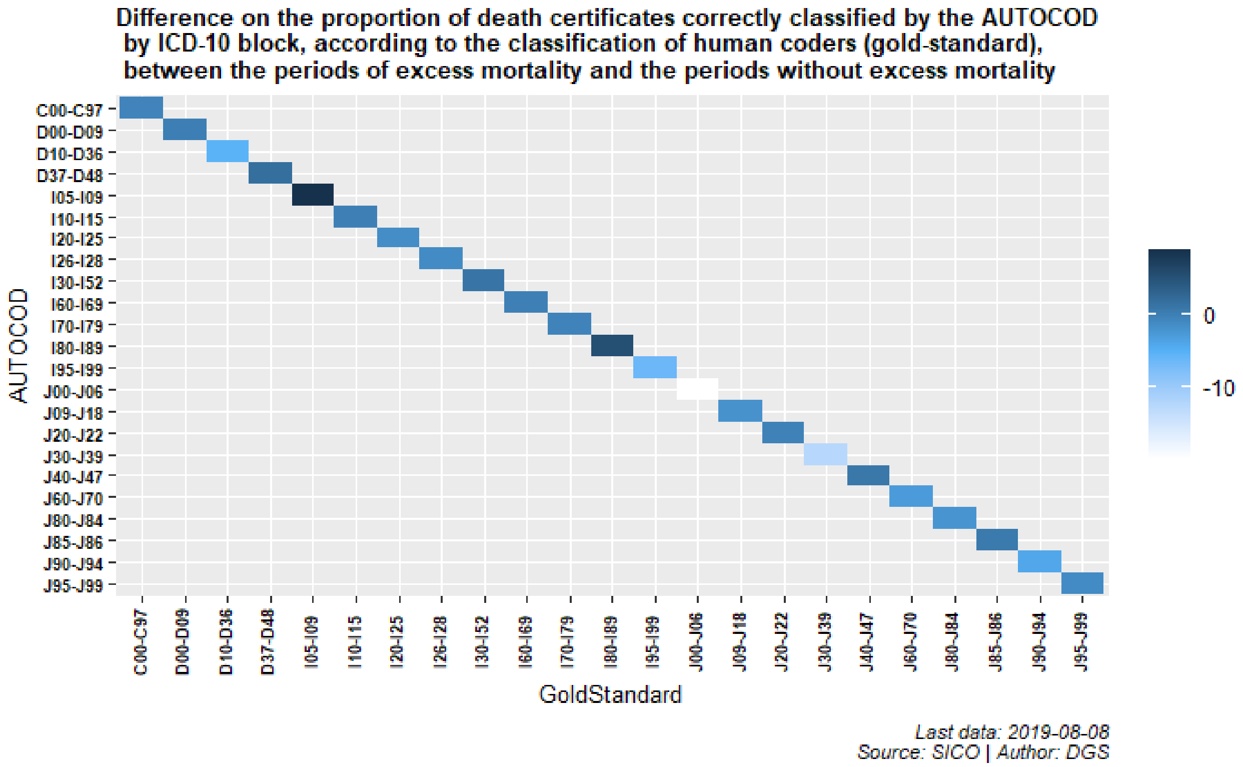

Supplementary Figure 32 - Difference on the proportion of death certificates correctly classified by the AUTOCOD by ICD-10 block, according to the classification of human coders (gold-standard), between the periods of excess mortality and the periods without excess mortality

Caption: C00-C97 - Malignant neoplasms; D00-D09 - In situ neoplasms; D10-D36 - Benign neoplasms; D37-D48 - Neoplasms of uncertain or unknown behaviour; I05-I09 - Chronic rheumatic heart diseases; I10-I15 - Hypertensive diseases; I20-I25 - Ischaemic heart diseases; I26-I28 - Pulmonary heart disease and diseases of pulmonary circulation; I30-I52 - Other forms of heart disease; I60-I69 - Cerebrovascular diseases; I70-I79 - Diseases of arteries, arterioles and capillaries; I80-I89 - Diseases of veins, lymphatic vessels and lymph nodes, not elsewhere classified; I95-I99 - Other and unspecified disorders of the circulatory system; J00-J06 - Acute upper respiratory infections; J09-J18 - Influenza and pneumonia; J20-J22 - Other acute lower respiratory infections; J30-J39 - Other diseases of upper respiratory tract; J40-J47 - Chronic lower respiratory diseases; J60-J70 - Lung diseases due to external agents; J80-J84 - Other respiratory diseases principally affecting the interstitium; J85-J86 - Suppurative and necrotic conditions of lower respiratory tract; J90-J94 - Other diseases of pleura; J95-J99 - Other diseases of the respiratory system

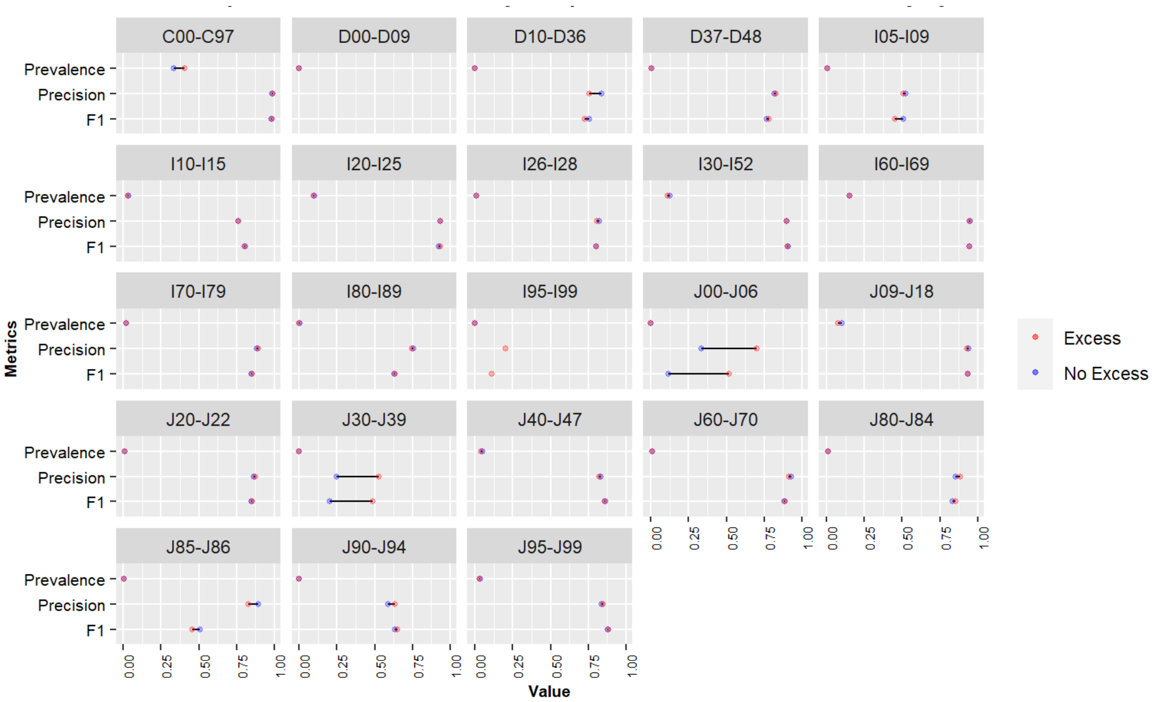

Supplementary Figure 33 - Comparison between other performance metrics of AUTOCOD during periods of excess mortality and periods without excess mortality, by ICD-10 block

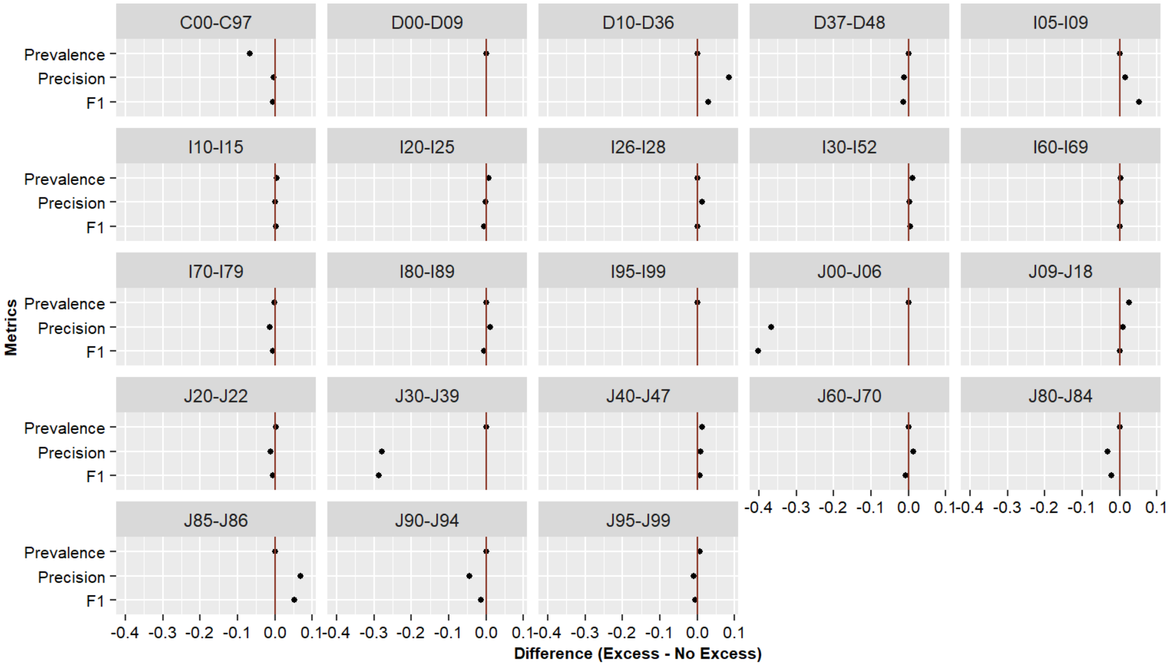

Supplementary Figure 34 - Difference on other performance metrics of AUTOCOD, by block, between periods of Excess Mortality and periods without excess mortality

Caption: C00-C97 - Malignant neoplasms; D00-D09 - In situ neoplasms; D10-D36 - Benign neoplasms; D37-D48 - Neoplasms of uncertain or unknown behaviour; I05-I09 - Chronic rheumatic heart diseases; I10-I15 - Hypertensive diseases; I20-I25 - Ischaemic heart diseases; I26-I28 - Pulmonary heart disease and diseases of pulmonary circulation; I30-I52 - Other forms of heart disease; I60-I69 - Cerebrovascular diseases; I70-I79 - Diseases of arteries, arterioles and capillaries; I80-I89 - Diseases of veins, lymphatic vessels and lymph nodes, not elsewhere classified; I95-I99 - Other and unspecified disorders of the circulatory system; J00-J06 - Acute upper respiratory infections; J09-J18 - Influenza and pneumonia; J20-J22 - Other acute lower respiratory infections; J30-J39 - Other diseases of upper respiratory tract; J40-J47 - Chronic lower respiratory diseases; J60-J70 - Lung diseases due to external agents; J80-J84 - Other respiratory diseases principally affecting the interstitium; J85-J86 - Suppurative and necrotic conditions of lower respiratory tract; J90-J94 - Other diseases of pleura; J95-J99 - Other diseases of the respiratory system

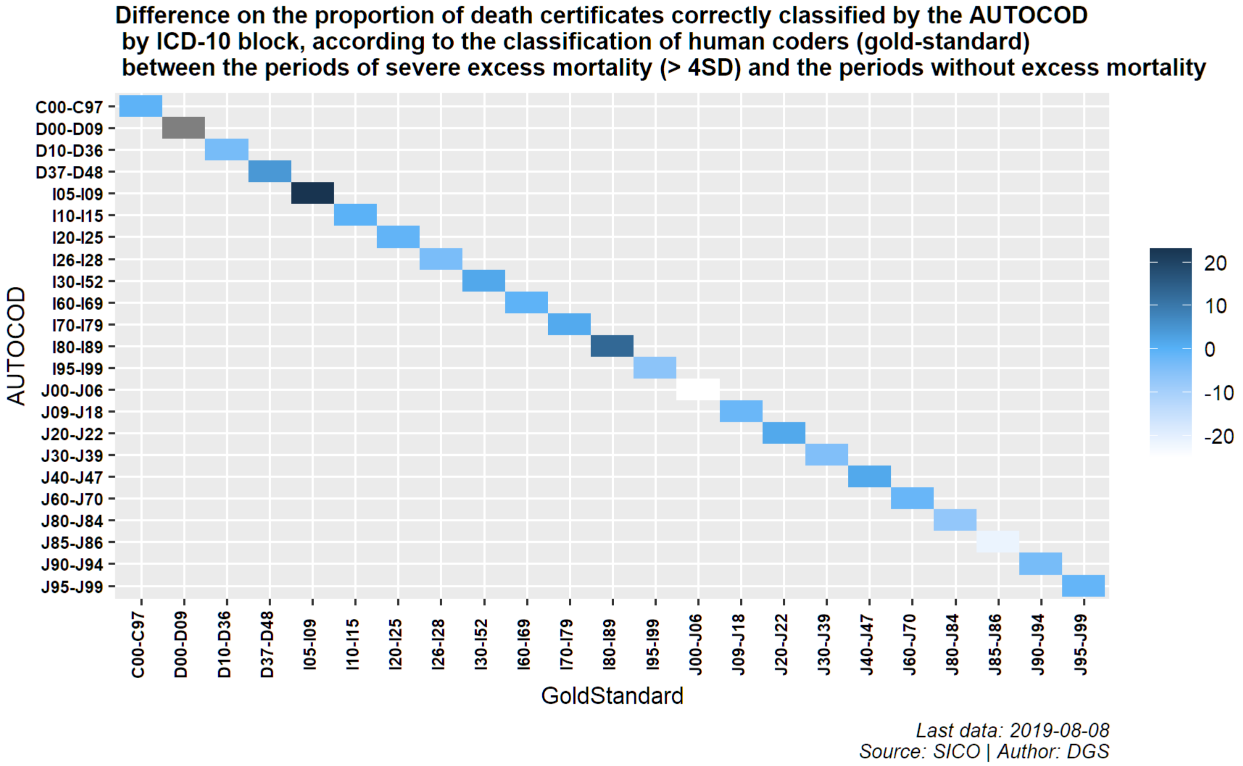

Supplementary Figure 35 - Difference on the proportion of death certificates correctly classified by the AUTOCOD by ICD-10 block, according to the classification of human coders (gold-standard), between the periods of extreme excess mortality (>4 SDs) and the periods without excess mortality

Caption: C00-C97 - Malignant neoplasms; D00-D09 - In situ neoplasms; D10-D36 - Benign neoplasms; D37-D48 - Neoplasms of uncertain or unknown behaviour; I05-I09 - Chronic rheumatic heart diseases; I10-I15 - Hypertensive diseases; I20-I25 - Ischaemic heart diseases; I26-I28 - Pulmonary heart disease and diseases of pulmonary circulation; I30-I52 - Other forms of heart disease; I60-I69 - Cerebrovascular diseases; I70-I79 - Diseases of arteries, arterioles and capillaries; I80-I89 - Diseases of veins, lymphatic vessels and lymph nodes, not elsewhere classified; I95-I99 - Other and unspecified disorders of the circulatory system; J00-J06 - Acute upper respiratory infections; J09-J18 - Influenza and pneumonia; J20-J22 - Other acute lower respiratory infections; J30-J39 - Other diseases of upper respiratory tract; J40-J47 - Chronic lower respiratory diseases; J60-J70 - Lung diseases due to external agents; J80-J84 - Other respiratory diseases principally affecting the interstitium; J85-J86 - Suppurative and necrotic conditions of lower respiratory tract; J90-J94 - Other diseases of pleura; J95-J99 - Other diseases of the respiratory system

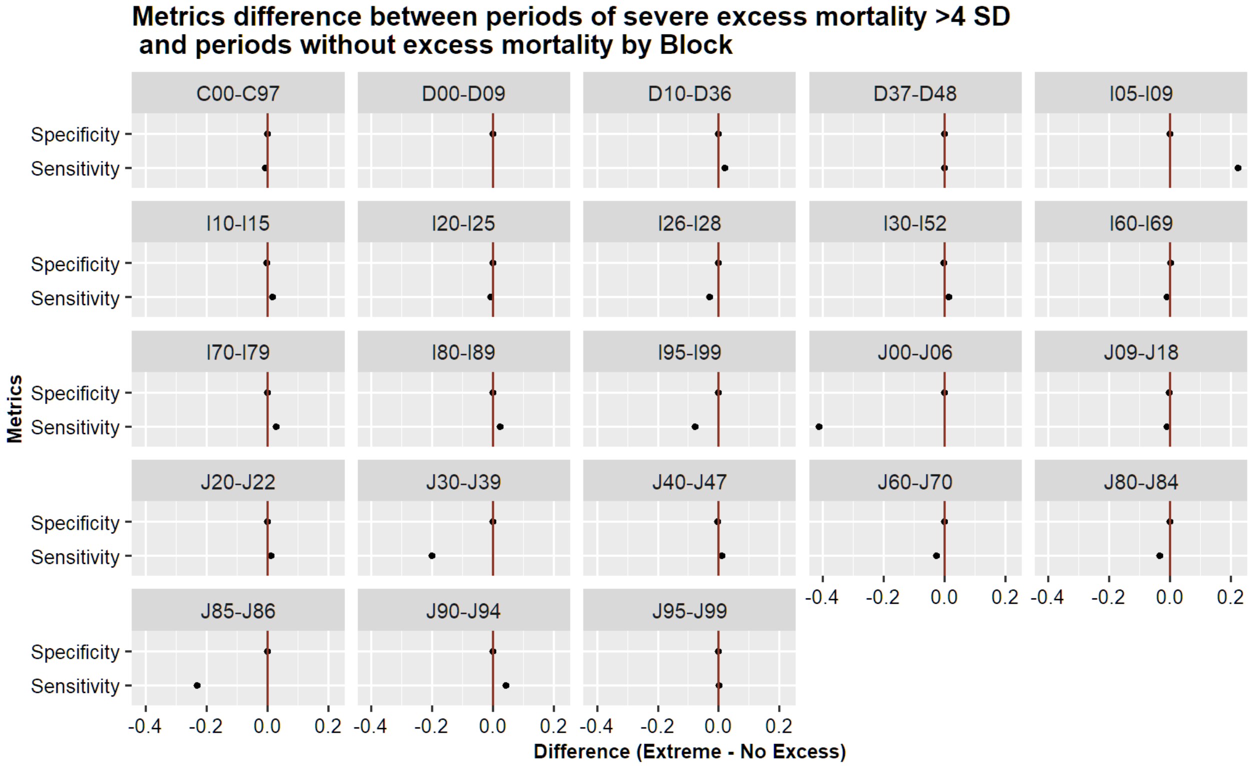

Supplementary Figure 36 - Difference on other performance metrics of AUTOCOD, by block, between periods of severe excess mortality (>4 SDs) and periods without excess mortality

Caption: C00-C97 - Malignant neoplasms; D00-D09 - In situ neoplasms; D10-D36 - Benign neoplasms; D37-D48 - Neoplasms of uncertain or unknown behaviour; I05-I09 - Chronic rheumatic heart diseases; I10-I15 - Hypertensive diseases; I20-I25 - Ischaemic heart diseases; I26-I28 - Pulmonary heart disease and diseases of pulmonary circulation; I30-I52 - Other forms of heart disease; I60-I69 - Cerebrovascular diseases; I70-I79 - Diseases of arteries, arterioles and capillaries; I80-I89 - Diseases of veins, lymphatic vessels and lymph nodes, not elsewhere classified; I95-I99 - Other and unspecified disorders of the circulatory system; J00-J06 - Acute upper respiratory infections; J09-J18 - Influenza and pneumonia; J20-J22 - Other acute lower respiratory infections; J30-J39 - Other diseases of upper respiratory tract; J40-J47 - Chronic lower respiratory diseases; J60-J70 - Lung diseases due to external agents; J80-J84 - Other respiratory diseases principally affecting the interstitium; J85-J86 - Suppurative and necrotic conditions of lower respiratory tract; J90-J94 - Other diseases of pleura; J95-J99 - Other diseases of the respiratory system

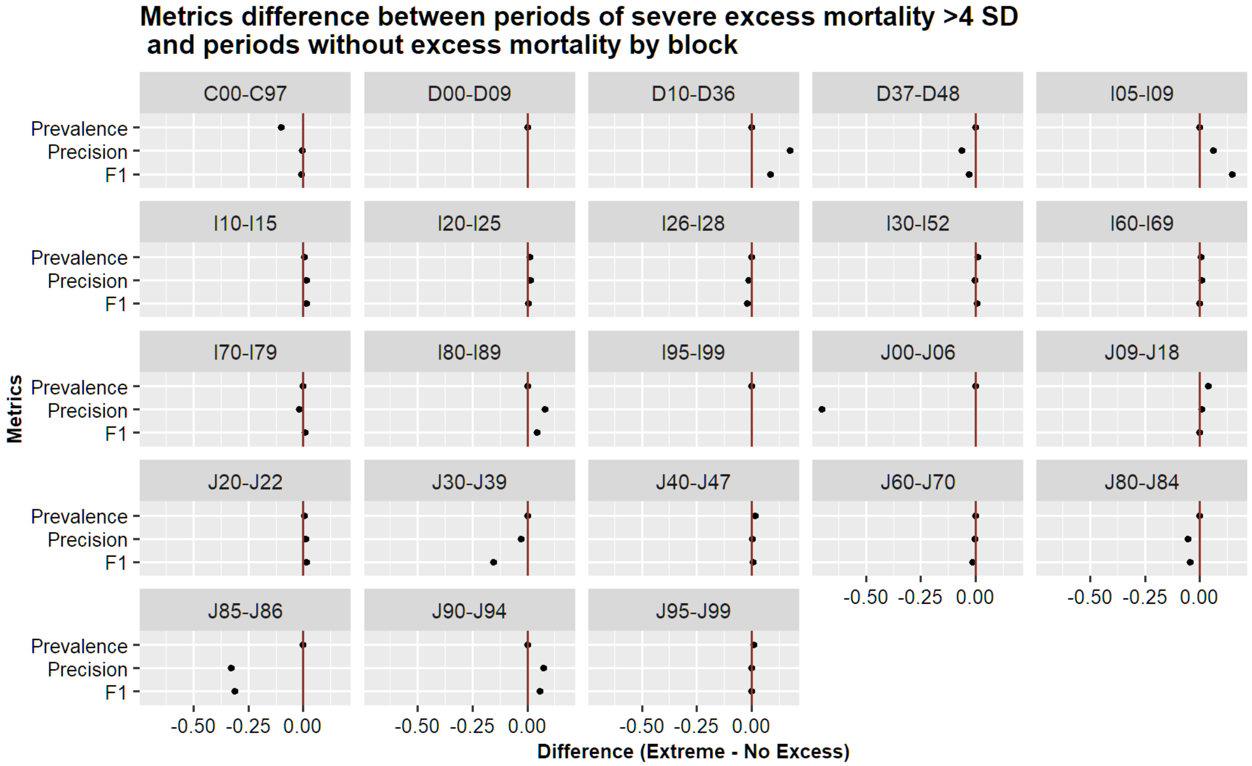

Supplementary Figure 37 - Difference on other performance metrics of AUTOCOD, by block, between periods of severe excess mortality (>4 SDs) and periods without excess mortality

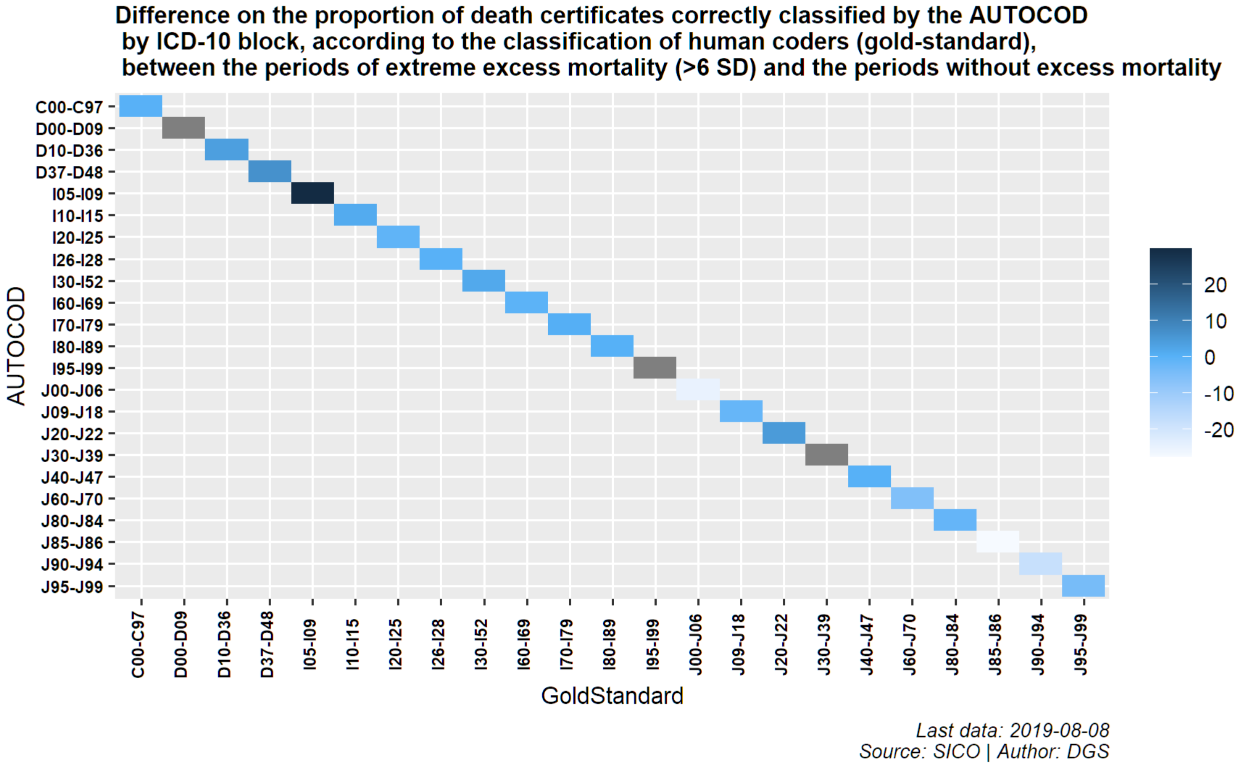

Supplementary Figure 38 - Difference on the proportion of death certificates correctly classified by the AUTOCOD by ICD-10 block, according to the classification of human coders (gold-standard), between the periods of extreme excess mortality (>6 SDs) and the periods without excess mortality

Caption: C00-C97 - Malignant neoplasms; D00-D09 - In situ neoplasms; D10-D36 - Benign neoplasms; D37-D48 - Neoplasms of uncertain or unknown behaviour; I05-I09 - Chronic rheumatic heart diseases; I10-I15 - Hypertensive diseases; I20-I25 - Ischaemic heart diseases; I26-I28 - Pulmonary heart disease and diseases of pulmonary circulation; I30-I52 - Other forms of heart disease; I60-I69 - Cerebrovascular diseases; I70-I79 - Diseases of arteries, arterioles and capillaries; I80-I89 - Diseases of veins, lymphatic vessels and lymph nodes, not elsewhere classified; I95-I99 - Other and unspecified disorders of the circulatory system; J00-J06 - Acute upper respiratory infections; J09-J18 - Influenza and pneumonia; J20-J22 - Other acute lower respiratory infections; J30-J39 - Other diseases of upper respiratory tract; J40-J47 - Chronic lower respiratory diseases; J60-J70 - Lung diseases due to external agents; J80-J84 - Other respiratory diseases principally affecting the interstitium; J85-J86 - Suppurative and necrotic conditions of lower respiratory tract; J90-J94 - Other diseases of pleura; J95-J99 - Other diseases of the respiratory system
